# Phylogenomic Analysis Reveals Extensive Phylogenetic Mosaicism in the Human GPCR Superfamily

**Published:** 2007-09-26

**Authors:** Robin G. Allaby, Mathew Woodwark

**Affiliations:** 1 Warwick HRI, University of Warwick, Wellesbourne, CV35 9EF, UK; 2 Cambridge Antibody Technology Ltd., Milstein Building, Granta Park, Cambridge CB1 6GH, UK

**Keywords:** evolution, GPCRs, mosaicism, phylogenomics

## Abstract

A novel high throughput phylogenomic analysis (HTP) was applied to the rhodopsin G-protein coupled receptor (GPCR) family. Instances of phylogenetic mosaicism between receptors were found to be frequent, often as instances of correlated mosaicism and repeated mosaicism. A null data set was constructed with the same phylogenetic topology as the rhodopsin GPCRs. Comparison of the two data sets revealed that mosaicism was found in GPCRs in a higher frequency than would be expected by homoplasy or the effects of topology alone. Various evolutionary models of differential conservation, recombination and homoplasy are explored which could result in the patterns observed in this analysis. We find that the results are most consistent with frequent recombination events. A complex evolutionary history is illustrated in which it is likely frequent recombination has endowed GPCRs with new functions. The pattern of mosaicism is shown to be informative for functional prediction for orphan receptors. HTP analysis is complementary to conventional phylogenomic analyses revealing mosaicism that would not otherwise have been detectable through conventional phylogenetics.

## Introduction

The G-protein coupled receptors (GPCRs) are a remarkably diverse superfamily of seven-transmembrane proteins that are responsible for intracellular G-protein activation on recognition of an extracellular ligand. A wide array of stimuli are recognised by GPCRs including light, odorants, calcium ions, nucleotides, lipids and peptides. The superfamily can be separated into at least five families that recognise diverse ligands (Bockaert and Pin, 1999; Fredriksson et al. 2003). Fredriksson et al. 2003 define these family groups as the Rhodopsin, Adhesion, Secretin, Glutamate and Frizzled receptor groups. These major divisions of the superfamily show little or no sequence similarity making phylogenetic inference about the origin of the GPCR superfamily problematic. The nature of the functionality of GPCRs makes them attractive targets for drug design; about half of all modern drugs act on GPCRs (Flower, 1999; Howard et al. 2001). Frequently, receptors that have not had their ligands characterised, so called orphan receptors, have their functionality inferred through phylogenetics. Consequently, an understanding of how GPCRs are related is highly desirable in the course of drug design. Each major family of GPCRs is itself made up of a number of groups. The phylogenetic relationships within these groups appear to be relatively clear, but it remains a challenge to ascertain how the different GPCR groups relate to each other within a family (Joost and Methner, 2002). Even within the rhodopsin family sequence conservation is too poor for good multiple sequence alignments across the whole family. To achieve robust phylogenetic analyses it is necessary to separate out the GPCR groups as relatively small but high quality alignments (Sjölander, 2004).

It is evident from the GPCR superfamily as a whole that sequence conservation is low, but a core tertiary structure is maintained. Consequently, any functional sequence conservation that does occur between sequences is likely to be phylogenetically local and possibly differential over the sequence length. It is possible then that sequence similarity high enough between GPCR groups to be identified as homology may only be fragmentary. The fragmentary nature of homology between sequences is exacerbated by the possibility that many GPCRs may be the result of recombination processes (Shields, 2000). This feature echoes findings that the majority of transmembrane and extracellular proteins are thought to be modular (Patthy, 1999), and the human genome appears to have a high number of modular genes (Li et al. 2001). Consequently, a biologically realistic strategy to reconstruct the phylogeny of the GPCRs should begin with the reconstruction of the phylogenies of those sequence fragments within GPCRs that can be identified as being homologous to other sequences.

**Figure 1.**
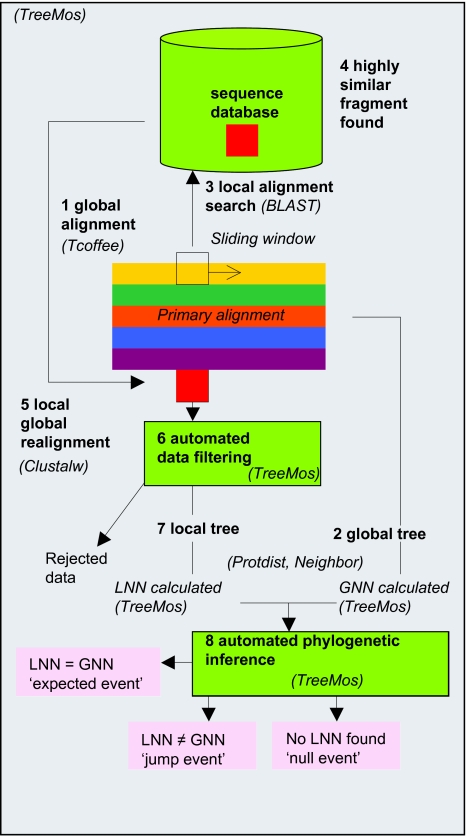
**Schematic overview of HTP analysis.** The analysis begins with a global alignment (1) from which a global tree is calculated (2). A sliding window is then moved down the alignment. The sequence within each window is subject to a BLAST search against a sequence database (3). If a sequence fragment is found which with a very low genetic distance (4), then this sequence fragment is globally aligned to the window (5). The window alignment is screened and filtered for high distances (6), and a local tree calculated based on the remaining data (7). The nearest neighbour to each taxon is then compared between the local tree and the global tree (8). The possible event outcomes of the analysis are shown in pink boxes. The programs employed at each stage of the analysis are shown in brackets.

It was the aim of this study to establish homology and reconstruct the phylogenies of sequence fragments within GPCR sequences across the whole rhodopsin family, and compare those to the phylogenies of the whole sequences within a GPCR group. In this way we hoped to extend the homologous relationships between GPCR groups on a fine scale and identify instances of potential recombination events through changes to expected phylogenetic topologies. In order to be able to identify relationships between the different GPCRs we elected to study sequence data from a single organism, humans. In this way more distant relationships will not be masked by close orthologous relationships. The experimental strategy was to construct phylogenetic trees for each GPCR group alignment, and then search for instances in which fragments of sequences were similar between GPCR groups and could be utilised in phylogenetic reconstruction. However, this approach rapidly leads to a high number of phylogenetic analyses all of which require careful interpretation of results. Consequently, we developed a novel high throughput phylogenomic (HTP) approach in which both phylogenetic tree construction and subsequent interpretation are automated, outlined in Figure 1. The analysis is phylogenomic because it uses all the known rhodopsin type GPCRs in the human genome. The robustness of phylogenetic interpretation is incumbent on underlying assumptions and validity of the input data. While the former is dependent upon the phylogenetic algorithm applied and was not further vetted in this study, the latter was subject to an automated quality control procedure that enabled a minimum quality of data input to be met for automated phylogenetic analyses. The stringent screening of data input and removal of data that is too distant made the novel large-scale automation of phylogenetic interpretation possible. Automated phylogenetic interpretation was limited to the identification of nearest neighbors. This automated approach enabled a powerful and comprehensive analysis of the rhodopsin GPCRs giving a novel perspective of the reticulate relationships across the family.

## Results and Discussion

**Figure 2.**
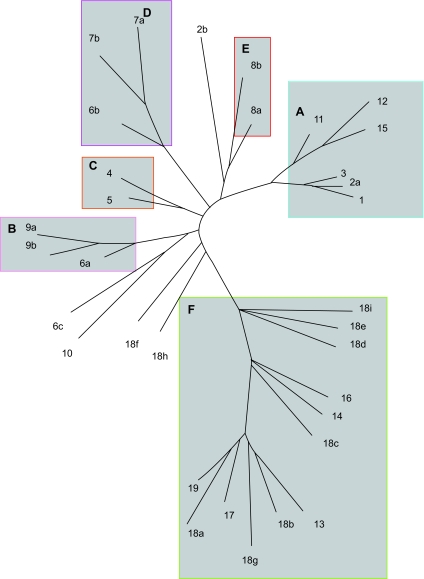
**Phylogeny of rhodopsin GPCR groups.** GPCR phylogeny based on reconstructed ancestors. Six principal clades are resolved within which genetic distances between reconstructed ancestors and extant sequences do not exceed κ. The principal clades are labeled **A**–**F** as follows. **A.** Nucleotide receptor group: includes peptide, nucleotides and lipid receptors. **B.** The endocrine hormone receptor group: includes peptide receptors involved with endocrine hormones. **C.** The opioid receptor group: includes opioid, somatostatin and galanin related peptide receptors. **D.** The neuropeptide receptor group: includes peptide receptors involved with endocrine hormones and neuropeptides. **E.** The mas receptor group: includes nociceptive mas and mas related receptors. **F.** The amine receptor group: includes biogenic amine, rhodopsin, arachodonic and peptide and lipid receptors. The remaining ancestors to groups are not included in major families because the genetic distance between them and all other taxa is aberrantly high.

We assembled 257 sequences of human rhodopsin type GPCRs into 19 alignments based on the groupings of Joost and Methner (2002), termed group 1–group 19. In order to improve alignments to meet our own minimum quality requirement criteria, we further subdivided the 19 alignments into 33 alignments such that each group did not yield a distance between any two taxa greater than the parameter κ (see methods), Table 1 (see also supplementary alignments). Alignment groups that had become subdivided to accommodate κ kept the same group names with a single letter suffix to denote the subdivided group. Consequently, each GPCR alignment contains closely related sequences for which a robust phylogenetic tree could be calculated. To interpret and test the significance of our HTP analysis we required a rhodopsin GPCR tree. A phylogenetic methodology of ancestor reconstruction was used to reconstruct the rhodopsin tree resulting in six major clades being identified, Figure 2 (see supplementary data for the complete treefile). The six major GPCR clades identified in this study represent the distance down the tree from the tips the ancestor reconstruction analysis could be carried out before the distance between ancestors exceeded our value for κ used in the HTP analysis. The topology of the tree is in broad agreement with that of Fredriksson et al. (2003). Both our tree and that of Fredriksson differ from the Joost and Methner (2002) organization by considering group 18 paraphyletic.

### HTP analyses of real GPCRs and the null data set

**Figure 3.**
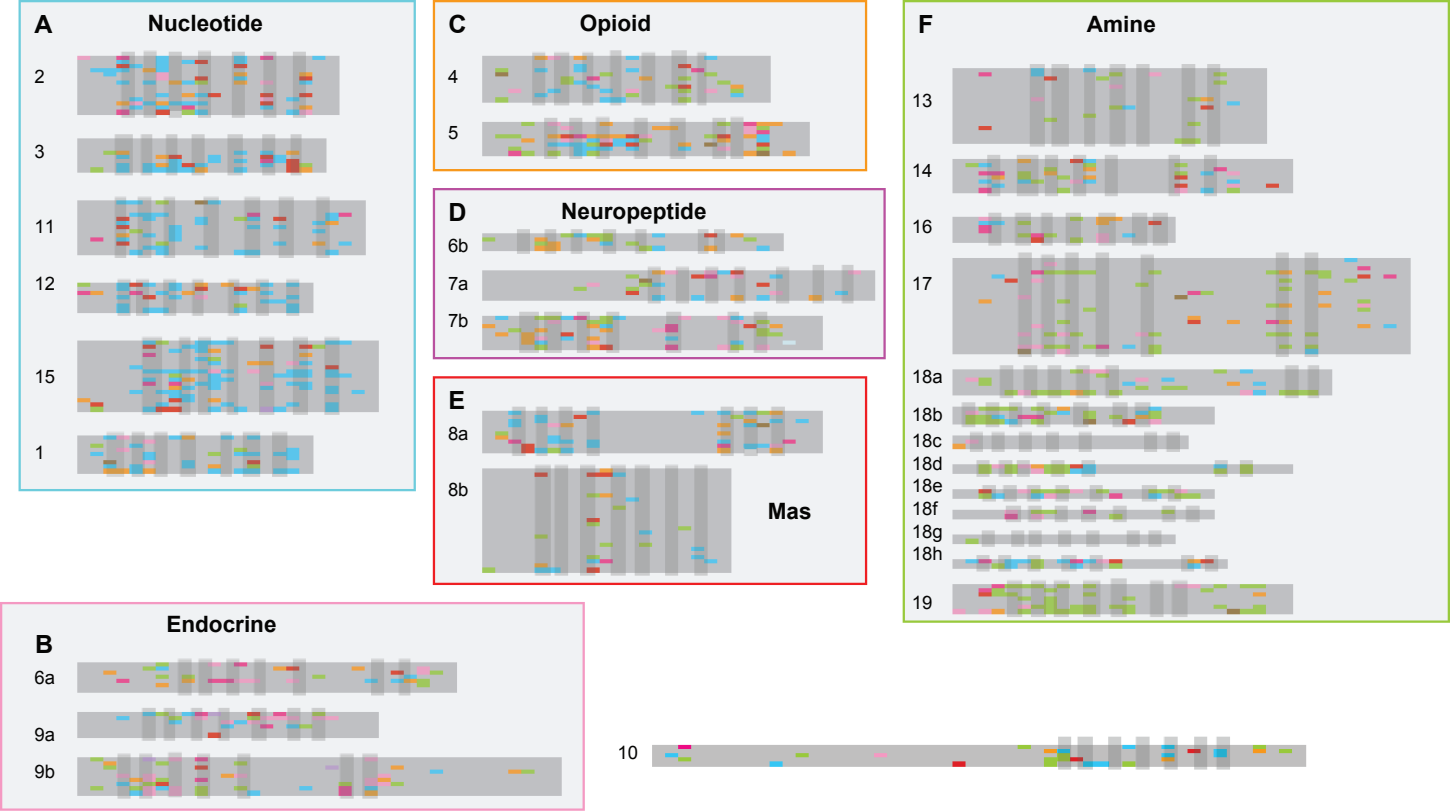
**Mosaic relationships of the rhodopsin GPCR groups.** Instances of phylogenetic mosaicism between GPCR groups are displayed. Colour signifies the major group from which the mosaic fragment derives: blue, nucleotide receptor group (**A**); pink, endocrine receptor group (**B**); orange, opioid receptor group (**C**); purple, neuropeptide receptor group (**D**); red, mas receptor group (**E**); green amine receptor group (**F**). Letters **A**–**F** correspond to major groups in Fig 2. GPCR groups not associated with a major group are colour coded as follows: group 1, white; group 2b pale blue; group 6c, violet; group 10 brown. Transmembrane domains are indicated in grey over the alignments. For details of specific receptor affiliations see Supplementary Figure 1.

**Figure 4.**
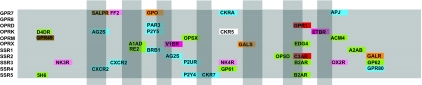
**Mosaicism in opioid receptor group (group 4).** Segmental phylogenetic affiliations resulting from HTP. Segments that have phylogenetic affiliations to receptors in GPCR groups other than group 4 are shown as colour blocks with the affiliated receptor name. Block colour corresponds to major GPCR group, shown in Figure 3. Transmembrane domains are indicated in grey over the alignment. For further details of affiliated fragment lengths see Supplementary Figure 1.

The HTP analysis of GPCRs produced 6041 phylogenetic outcomes representing a maximum of 18,123 phylogenetic analyses. Phylogenetic mosaicism occurred frequently, data indicating 46% of all phylogenetic outcomes found jump events for taxa in all the GPCR groups. Furthermore, 18% of all events were jump events between different GPCR groups, Figure 3, Supplementary Figure 1. As an example, the opioid receptor is shown in more detail in Figure 4.

To help identify the underlying reasons for the phylogenetic mosaicism we compared the real data with a simulated null hypothesis GPCR data set in which no homogenisation through recombination had occurred. The null data set was generated by simulating mutations to a random seeded sequence to represent the evolutionary pattern of divergence illustrated in the phylogenetic tree for GPCRs in Figure 2, producing 257 new sequences. A HTP analysis was carried out on the null data set resulting in 4590 phylogenetic outcomes reflecting a maximum of 13,770 phylogenetic analyses (data not shown). A level of phylogenetic mosaicism was apparent in this second analysis which is similar to that found with the real data set, with 52% percent of all phylogenetic outcomes being jump events, and 16% of all outcomes being jump events between groups. However, only 2% of phylogenetic outcomes were null events, where no neighbour could be identified, considerably less than the 12% observed with the real data. Differences between the two data sets are further highlighted by paired T-tests for the relative frequencies for each of the nineteen possible phylogenetic outcomes that showed a significant difference in all but two cases (Supplementary Table 1). Mosaicism between families, represented by only one outcome type, did not differ significantly between the real and simulated data sets. However this apparent similarity is strongly reduced by a lurking variable regarding the phylogenetic distance of jumps. The phylogenetic distance represented by jump events did differ significantly between the two data sets (Supplementary Table 2). There is a threefold increase in the frequency of jumps over just 1– 4 nodes in the real data, significant at the 0.01 level. Consequently, there are many more jumps between closely related GPCR groups than would be expected from homoplasy. Conversely, results obtained with the real and simulated data sets for jumps of larger phylogenetic distance were similar. This indicates that the resemblance of sequence fragments between distantly related GPCR groups is frequently attributable to homoplasy. It also implies that functional constraint on sequence change is negligible across large distances in the family because differentially conserved sequences in different groups would be expected to lead to a greater number of instances of mosaicism at this level.

### Patterns of mosaicism and possible underlying evolutionary models

Several interesting patterns are discernible in the phylogenetic mosaicism between GPCR groups shown in Figure 3. Firstly, there is a notable tendency for a higher number of mosaic events between groups of the same major groupings, as defined in Figure 2. Secondly, the similarity between fragments associated in an instance of phylogenetic mosaicism can be extremely high. Thirdly, mosaicism appears to be frequently correlated for members of the same family. Fourthly, similar mosaicism is frequently repeated in individual sequences.

**Figure 5.**
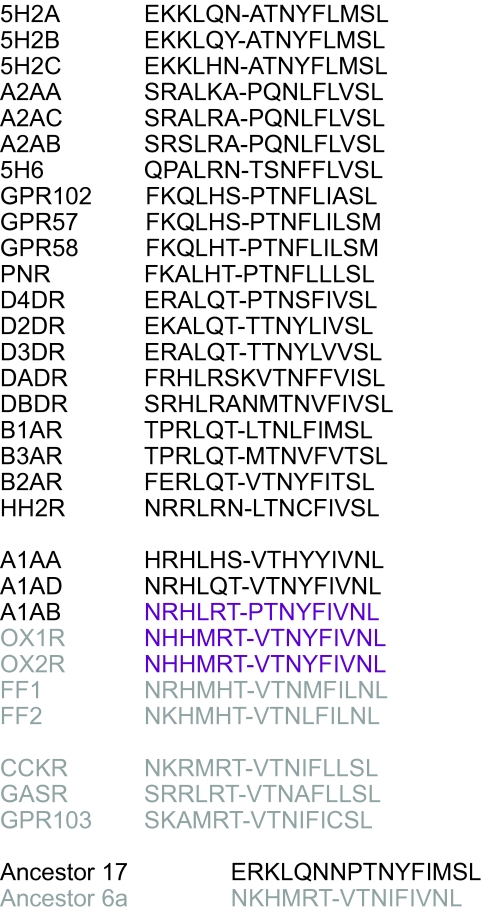
**Amino acid alignment of phylogenetic mosaicism between orexin and adrenergic receptors in the first cytoplasmic domain**. Alignment of the first cytoplasmic domain in the gastrin and the adrenergic receptor groups. Adrenergic group receptors are shown in black, gastrin group receptors in grey. The nearest neighbors identified by the HTP analysis, orexin and alpha-1B adrenergic receptor, have matching residues highlighted in purple bold. The reconstructed ancestral sequence for the adrenergic (ancestor 17) and gastrin (ancestor 6a) receptors are shown at the bottom of the alignment.

The high similarity that can occur between fragments is shown in the example of phylogenetic mosaicism between the gastrin receptor group and the biogenic amine receptors, Figure 5. In this case the HTP analysis detected a region of phylogenetic inconsistency in the first cytosolic region between transmembranes 1 and 2 of the orexin receptors and the alpha-1B adrenergic receptor. This region is generally considered to be among the most conserved in GPCRs, but within both the biogenic amine and gastrin receptors group the fragment is highly variable in sequence composition. The similarity between the subset of sequences from these two distant groups is striking and highly significant statistically—a local alignment search would only expect to find 0.0008 sequences this length and similarity (E value = 0.0008). This level of probability makes homoplasy as an explanation of the sequence similarity in this case highly doubtful. One explanation might be that this segment was strongly conserved in these two disparate lineages from antiquity because of an important function. Such an explanation would predict that both the alpha 1 adrenergic and orexin receptors were placed at a basal position in their respective phylogenetic groups, and one would also expect to the see the conserved sequence in the respective ancestors. The orexin receptor ancestor does indeed have a sequence very similar to that shared by the two disparate lineages, although they do still share a character state to the exclusion of the ancestor. This supports the idea that this particular region of sequence has been under considerable functional constraint in the gastrin receptors. However, the alpha 1 adrenergic receptors are not basal to their phylogenetic group (see GPCR tree, supplementary data), and the reconstructed ancestor for this group does not resemble the sequence shared by the two disparate lineages. The phylogenetic data are incompatible with the idea that the segment could have been conserved in the form seen in the alpha 1 adrenergic receptor lineage. A more likely explanation is that the functionally conserved segment from the orexin receptor or its ancestor was donated to the alpha 1 adrenergic lineage some time after the gene duplication event that produced the alpha 1 and alpha 2 lineages, but before the diversification of the alpha 1 lineages. Immediately after the duplication event that split the alpha 1 and 2 lineages, it is likely that functional constraint would have been relaxed on either one of the lineages while the other continued the original function. Therefore the evolutionary opportunity occurred in which the alpha 1 lineage could receive new sequence through recombination without a costly loss of function to the host organism. It is particularly interesting then that the sequence fragment in question appears to be one that is functionally important implying that this may have been an important mechanism in GPCR evolution to produce GPCRs with new function.

**Figure 6.**
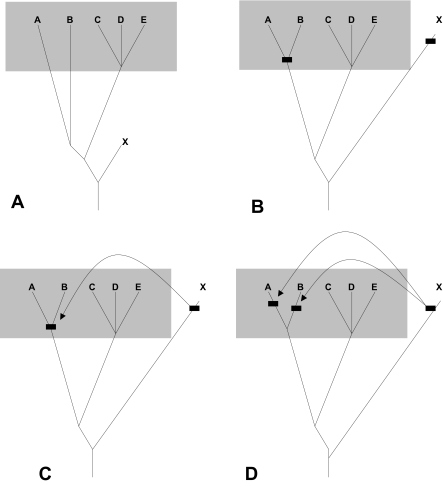
**Four phylogenetic models of correlated mosaicism. A** monophyletic group is represented by taxa **A**–**E**. Taxa **A** and **B** display correlated mosaicism to distantly related taxon **X. A.** Deep branches within the group **A**–**E** result in both **A** and **B** being closest to **X. B**. At some point in the past indicated by boxes on the tree, the ancestor to **A** and **B** becomes so similar to the ancestor of **X** by chance homoplasy that both **A** and **B** appear closest to **X. C. A** recombination event between **X** and the common ancestor to **A** and **B** results in both taxa appearing to be closer to **X** than each other and **C**, **D** and **E. D**. Multiple recombination events between **X** and the **A** and **B** lineages respectively results in both taxa resembling **X** more than each other. Branch lengths are proportional to genetic distance.

**Figure 7.**
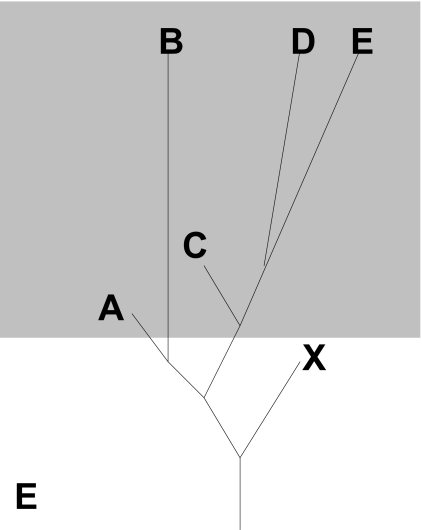
**A fifth model of correlated mosaicism.** Two non nearest-neighbor members (**A** and **C**) of a monophyletic group have short branch lengths and so resemble outgroup **X** more than they do each other, or other members of the group.

Another pattern is that of correlated mosaicism. Correlated mosaicism is the condition in which multiple members of a group have the same or similar instances of mosaicism. Striking examples of this occur for group 17 in which many members of the group display phylogenetic mosaicism relating to the endocrine receptor group, in particular the orexin receptor, but also receptors of group 6a. In several other members of the same group there is phylogenetic mosaicism with somatostatin receptor 3 (group 4) in the latter part of the alignment. Other instances of correlated mosaicism occur throughout the rhodopsin GPCRs, such as a similarity of multiple Mas type receptors fragments to opioid receptor D in the latter half of the alignment; several members of the angiotensin receptor group to the Mas group in the latter part of the alignment. The opioid receptor group in Figure 4 shows correlated mosaicism to the Mas receptors indicating that there is a reciprocal relationship between the Mas type receptors of group 8a and the opioid receptor D. In the same region of the opioid group there is correlated mosaicism between somatostatin receptors three and five, and the beta-2 adrenergic receptor of group 17. A model in which segments of GPCR receptors are differentially conserved in different families could not explain correlated mosaicism. Members within a family would be expected to appear more closely related to each other than outside taxa because they would be derived from a conserved common ancestor. Four phylogenetic models could be forwarded to explain the non-random pattern of correlated mosaicism, outlined in Figure 6. In the first model (Fig. 6A), two or more group members that display mosaicism to an ‘outside’ member from a different group may have had high rates of change in the past resulting in long branches. The result is that the genetic distance between the two group members is actually higher than that between either and the outside member. This explanation requires that the branch leading to the outside member be correspondingly short, which becomes increasingly problematic over very long periods of time, but as observed in the orexin receptor example above has occurred to some extent. This model also requires that the taxa at positions A and B in Figure 6 become very divergent to each other and relative to the family, which is not the case in the examples cited above. Another model requirement is that there is some considerable distance between X and both A and B, which as outline from the example above is not the case. In the second phylogenetic model (Fig. 6B) the ancestor to taxa A and B was very similar to the outsider taxon X due to homoplasy. Again in this model there is a higher rate of change in the lineages of A and B relative to X resulting in both A and B appearing to be more like X than each other. This model is problematic because in order for it to work the homoplasy must have been so extensive as to be an exact match between the lineages leading to A/B and X in order for A and B not to resemble each other more than X. The statistics of the example shown in Figure 4 demonstrate that such extensive homoplasy is unlikely in this data set. In the third phylogenetic model (Fig. 6C) the same pattern of genetic distances as in model 2 could result from a recombination event between the outside member and the common ancestor of the two members displaying correlated mosaicism. This model requires the point of ancestral similarity to be an instance of identity, and so works more credibly than the second model. Alternatively, model 4 shown in Figure 6D attributes each instance of mosaicism to a separate recombination event. This is the only model that does not require a lower rate of change in X from a factor such as functional constraint. This model would be consistent with one which results from the underlying genomic architecture, in which a tendency for recombination between certain regions of receptors of very different groups due to genomic position or processes at the time of recombination. This explanation would predict that the pattern of correlated mosaicism would be due to multiple unrelated events. Alternatively, one may not put the restriction on that the two taxa involved in mosaic correlation are nearest-neighbors. Under this condition, a fifth model also becomes possible, Figure 7. In this case the branches leading to the correlated taxa (A and C) are short representing conservation of sequence. This model appears the most plausible one in which recombination does not occur. However, it requires the taxa concerned to occur at a basal position in the phylogeny, and that branch lengths vary widely within the GPCR group. It is generally not true that branch lengths vary so widely in the GPCR phylogeny, and rarely the case that correlated taxa are basal, as in the case outlined in Figure 5. Of these five phylogenetic models, those that do not involve recombination have serious phylogenetic problems. This leaves the recombination-based models as the most likely explanations for correlated mosaicism.

The final pattern to emerge from Figure 3 is a tendency for some receptor sequences to show repeated mosaicism to the same or similar receptor. For example, neuromedin receptor 1 (group 7b) displays mosaicism repeatedly with somatostatin receptor 4 (group 4). Another example is that of GPR87 of group 12, which shows two instances of mosaicism with neuromedin receptors. In the opioid receptor group, somatostatin receptor three shows repeated mosaicism to tachykinin receptors three and four, Figure 4. Again, such a pattern is not easily explained by homoplasy because it would require several independent instances of the same homoplasy happening, which is improbable. In this case again the model of functional constraint acting in several different places along receptors of different families resulting in an apparent similarity through shared plesiomorphy might be invoked. However, such an explanation predicts the sequences to be placed in a basal phylogenetic position in order for the ‘conserved’ regions to have remained unchanged from the ancestor. This is not the case for the neuromedin receptors, GPR87, tachykinin receptors 3 & 4, or somatostatin receptors 3 & 4. Again, the alternative explanation is one involving recombination in which a single recombination event may have resulted in several segments along the length of the receptor gene sequence being exchanged.

**Figure 8.**
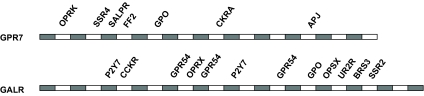
Complete mosaicism of GPR7 and GALR receptors. Segmental affiliations resulting from HTP. Instances of in group mosaicism and between group mosaicism are included. The window partitions of the sequences are represented as bars.

The findings of this study are compatible and build on the findings of Shields (2000). Shields (2000) examined evidence of gene conversion events that were more recent to the events described in this study, after the split of the murine and human lineages. This was achieved through the comparison of paralogs in several different species. Character states which conflicted with the phylogeny by appearing as synapomorphies within a pair of paralogs in a single species were the source of gene conversion evidence. The study convincingly demonstrated frequent recent gene conversion events, including ones between CXCR1 and CXCR2 in humans and between murine CCR2 and CCR5. These examples represent homogenisation events at the most external nodes of the GPCR tree, and as such are beyond the level of detection of this study. The paralogs examined above were already nearest phylogenetic neighbors within a single species, so any gene conversion will not lead to phylogenetic mosaicism within one species set of GPCRs. A prediction from Shield’s study would be that where non-nearest phylogenetic neighbors occur adjacent to each other on a chromosome, then one would expect instances of phylogenetic mosaicism to occur between the two non-neighbors if homogenization processes had acted between the two. There are two clear instances in which non-neighbors are adjacent in their loci without any other GPCR loci nearby for GPR7/opioid in either direction occur at q22.3 and p12. The galanin R and urotensin receptors occur on chromosome 17 at q25.3 near the telomeric region, the next nearest loci being at q25.1. Figure 8 shows all the mosaic jumps for GPR7 and galanin R receptors respectively. In both cases there is a phylogenetic signal indicating a jump to their respective adjacent loci, OPRK in the case of GPR7, and UR2R in the case of GALR. It would appear that the data support the predictions from Shield’s study for adjacent recombination suggesting the process is ongoing.

The approach used in this study enabled us to illustrate that process Shields (2000) observed kappa receptors and galanin/urotensin receptors. GPR7 and the opioid kappa receptor occur together on chromosome 8 at q11.23, the next nearest loci at one tip of GPCR tree appears to have an impressive and deep history within the GPCRs. Both this study and that of Shields (2000) found that homogenization events have been mostly between GPCRs that are close on the phylogenetic tree. However, the more extensive surveying enabled in this study demonstrates that the phylogenetic restriction is not entirely to the exclusion of wider jumps. This wider jumping may be a result of genomic architecture at the time of homogenization.

### The mode of evolution of the rhodopsin GPCRs

The establishment of GPCR phylogeny has been problematic in the past because of the high level of sequence divergence and character conflict associated with low conservation. The four lines of evidence in this phylogenomic analysis (1. high frequency of mosaicism 2. high similarity of mosaicism 3. correlated mosaicism 4. repeated mosaicism) build up a picture of genomic fluidity for rhodopsin GPCRs.

A model of mosaic evolution for GPCRs is congruent with the observation that only a few residues are involved with the allosteric interactions of signal transduction (Shulman et al. 2004). The 7 transmembrane structure is highly conserved in GPCRs, so it is likely that in many cases one could recombine sections of sequence from different GPCRs and maintain the tertiary structure. Consequently, it is biologically plausible that segments of GPCR may have been swapped frequently during the evolution of the superfamily without loss of function, and in some cases resulted in functional modification.

It was noted during this study that some GPCR groups displayed more mosaicism than others. Under the ‘fluid genome’ model of evolution that appears to represent the rhodopsin GPCRs, it may be predicted that groups that have recently expanded are less likely to display instances of mosaicism. In the case of such expansions, less time has elapsed so fewer recombination events are expected relative to other groups. The data presented in Figure 3 would appear to support this prediction in the case of group 8b, the Mas related receptors, which has been identified as a recently expanded group (Dong et al. 2001; Choi et al. 2003). The data also suggest that group 13 may be a relatively young group also.

### Potential of tracking mosaicism for functional prediction

A pharmacologically important question is to what extent does phylogenetic mosaicism affect GPCR function? The emergent model of GPCR evolution discussed above suggests that recombination has occurred in the past without loss of signal transduction function, and possibly provided a means to generate new function, as exemplified in the orexin/alpha 1 adrenergic example in Figure 5. Such swapping of functionally important segments suggests that evidence for function may be present in the patterns of mosaicism. If the ability to track cryptic instances of similarity between GPCR receptors due to phylogenetic mosaicism is useful in the process of assigning function to orphan receptors by guiding the choice of ligands to test, then the data presented in Figure 3 (and in more detail in Supplementary Figure 1) could be a valuable tool to pharmacologists.

The case history of GPR7 supports the prediction that GPCR function may be inferred from mosaicism evidence. Until recently, the natural ligand for GPR7 was unknown. GPR7 is placed within group 4 that includes opioid and somatostatin receptors (Fig. 4), although these molecules are not ligands for the GPR7 receptor. There are instances of mosaicism between GPR7 and fragments of sequence from groups 2a, 3, 5 and 6a (Figure 4). Interestingly, GPR7 has a fragment that represents similarity to SALPR, a receptor known to bind the hormone relaxin (Liu et al. 2003). Recently it has transpired that it is the case that GPR7 binds relaxin (Hsu et al. 2002). The GPR7 receptor also has a fragment that is affiliated with neuropeptide FF receptor 2. The neuropeptide FF receptor 2 occurs in the group 6a, which includes receptors for neuropeptides involved with food intake and the endocrine system. The natural endogenous ligands for GPR7 have been identified as neuropeptides B and W (Tanaka et al. 2003), which are involved with the endocrine system and food intake signals. Consequently, this data could have alerted pharmacological workers to test for the relaxin and neuropeptide ligands involved with the endocrine system in the case of GPR7. The value of being able to track mosaicism is potentially immense in terms of a directed approach to ligand identification. Orthologs can differ in function (Allaby and Woodwark, 2004) so a high global sequence similarity between sequences does not necessarily equate to a functional similarity. This is the case for the GPR7 receptor. An ability to trace local similarities between relatively distant sequences is a powerful tool for suggesting possible alternative functions in the absence of a complete understanding of the specific residues involved in signal transduction and ligand interaction.

### A novel approach for phylogenomics

Phylogenomics is the intersection of evolution and genomics (Eisan and Fraser, 2003). The field of phylogenomics uses gene trees overlaid with experimental data in order to generate hypotheses of possible functions of new genes based on their phylogenetic placement (Eisan, 1998). Considerable effort has been directed at extending the search for homologous sequences of low similarity (Sjölander et al 1996), using a common tertiary structure to help identify homology (Sjölander, 2004). In this study we have developed a complementary alternative approach in which we searched for fragments of high similarity and overlaid the phylogenetic inference of that information with both global trees and experimental data. As a result of analysing only high quality data the analysis, both phylogenetic tree construction and interpretation could become highly automated. The HTP analysis in this study provides a general powerful approach that could be applied to other gene families to track the mosaic origins of sequence fragments against the backdrop of the entire genome. This mosaicism may have an influence on the function of the protein, although the caveat should be noted that sequence similarity does not necessarily equate to functional similarity, orthologs frequently have different functions (Allaby and Woodwark, 2004).

## Methods

### Sequence data and alignments

Sequence data were utilised from Joost and Methner (2002). Groups were aligned using T-Coffee (Notredame et al. 2000). Pairwise distances were calculated using PROTDIST from the PHYLIP package (Felsenstein, 1989). The alignments are available with the program TreeMos (see below).

### HTP analysis

HTP analysis was carried out using the perl program TreeMos written by R.G. Allaby (available on request, currently runs by command line). TreeMos performs HTP analyses on a single alignment, the primary alignment, with respect to a group of alignments utilising the programs NEIGHBOR, PROTDIST, CLUSTALw and BLAST, outlined in Figure 1. The global tree for the primary alignment is calculated using NEIGHBOR of the PHYLIP package (Felsenstein, 1989) (step 1, Fig. 1) from which the global nearest neighbour (GNN) is calculated (step 2, Fig. 1). A GNN is an operational taxonomic unit that may be either a single taxon or a clade of taxa. A sliding window size of 20 amino acids is considered appropriate in this analysis, which although small corresponds to the size of a transmembrane domain. A sliding window of the primary alignment is then analysed as follows. A distance matrix is generated for each window using PROTDIST. A primary aim of the automation is to control data entry into the HTP to reflect the underlying biology realistically. This is achieved by screening the distance matrix for distances over a defined limit (step 6, Fig. 1). Taxa are iteratively removed from the matrix beginning with those with the highest number of values exceeding the parameter κ. The parameter κ is selected to represent a value above which genetic distances are deemed aberrantly high and not considered reliable. A value of κ equivalent to 400 times as many substitutions as would be expected to separate 1% divergent sequences (400 PAMS) was selected for this analysis (Allaby and Woodwark, 2004). The resulting matrix is viable if there are four or more taxa in it, or non-viable if there are less. Each taxon is then subject to a phylogenetic analysis to ascertain whether the local nearest neighbour (LNN) is the same as the GNN (step 8, Fig. 1). An outline of the phylogenetic decision process is given in Supplementary Figure 2. Two further phylogenetic analyses are then carried out. Both begin with a local alignment search (step 3, Fig. 1) using BLAST (Altshcul et al. 1997). In the first analysis, the database searched against is built from the whole of the primary alignment, in the second the whole sequence database is considered. If the local alignment search identifies a sequence fragment that has a genetic distance lower than the highest distance within the matrix for the window, and is less than κ (step 4, Fig. 1), then that sequence fragment is aligned to the window (step 5, Fig. 1) using clustalw (Thompson et al. 1994). The LNN is calculated as previously described through steps 6 and 7 in Figure 1, and an automated phylogenetic inference carried as described in step 8. For each fragment there are three classes of outcome possible. An ‘expected event’ is one in which the GNN and LNN were the same. A ‘jump event’ is one in which the LNN is different to the GNN. Thirdly, a ‘null event’ is one in which no LNN could be assigned due to aberrantly high distances or missing data.

### Ancestor reconstruction and GPCR tree

The GPCR tree was produced by a ‘tips down’ approach in which ancestral sequences were constructed for each of the GPCR groups that in turn could be compared directly (Supplementary Data). In those groups with more than three members a maximum likelihood tree was calculated using PROTML from the PHYLIP package (Felsenstein, 1989). Characters were traced to the root of the tree using the parsimony character trace in Mesquite (Maddison and Maddison, 2003). Sites at which multiple ancestral character states were equally parsimonious, a likelihood calculation was used to choose the most likely ancestor. Ancestor sequences were aligned using T-Coffee (Notredame et al. 2000), sub-grouped to remove aberrant distances, and realigned. Maximum likelihood trees were calculated for the ancestor sequence groups. The process of ancestor reconstruction was reiterated for the ancestor groups, and above process repeated to produce deeper ancestor subgroups. For the purposes of the simulated GPCR set the major ancestor groups, and remaining ancestors were united in a maximum likelihood tree. The GPCR tree topology was then completed by replacing the ancestor sequences sequentially with the maximum likelihood topologies they represented. Finally, the tips of the GPCR tree were replaced with the maximum likelihood topologies of the separate groups.

### Simulated GPCR set

The simulated GPCR set was produced using the PSeq-Gen 1.1 program (Grassly et al. 1997). A 400 amino acid seed sequence was mutated using a Monte Carlo simulation following the GPCR tree topology along the given branches such that 257 new sequences were produced. A Jones Thornton Taylor (1992) model of amino acid substitution was specified in the simulation.

### Statistics

#### Comparison of phylogenetic event outcomes

The differences in frequencies of the phylogenetic event outcomes between the real and simulated GPCR sets were analysed using a two pair T-test. The frequencies of event types were standardised for each group so that the total frequency was 200 (see Supplementary Data). A two pair T test was then applied to each event type, considering the frequency values in corresponding groups between the real and simulated data (Supplementary Table 1).

#### Comparison of phylogenetic distance of jump events

Jump events were categorized for each group by the number of nodes in the GPCR tree in Figure 2 transgressed between the taxon and the LNN. Phylogenetic nodes within groups were not included in the measurement, and only groups with four or more taxa were included. The frequencies of jumps that transgressed 1–4 nodes were then calculated for each GPCR group, and standardized to reflect a total of 5000 phylogenetic events. The differences in standardized frequencies of jump event types between the real and simulated data were analysed using a Mann Whitney test. A value of U of 131 was calculated, and a critical value of U at the 0.01 level is 175, therefore the data sets are significantly different (see Supplementary Table 3).

#### Comparison of frequencies of null events

A Mann Whitney test was also applied to test the significance of frequencies of null events in the real and simulated data sets. Only null events arising from high genetic distances were considered, null events arising from absent data from gaps in alignments were not included. Null events were calculated per group, and then standardized to reflect a total of 5000 phylogenetic outcomes in order to compare the real and simulated data sets. A value of U of 113.5 was calculated, and a critical value of U at the 0.01 level is 175, therefore the data sets are significantly different (see Supplementary Table 4).

## Supplementary Materials

Supplementary alignments

Supplementary Data

Supplementary Figure 1

Supplementary Figure 2

Supplementary Tables

## Figures and Tables

**Table 1..** GPCR groups of the rhodopsin family used in this study. Adapted from Joost and Methner (2002).

**GPCR group**	**Receptor ligand types**
group 1	CC chemokines
group 2a	CXC chemokines
group 2b	duffy
group 3	angiotensin, apelin, bradykinin
group 4	opioids, neuropeptide B, nociceptin, somatostatin
group 5	galanin, melanin-concentrating hormone, kisspeptin, leukotriene-B4, somatostatin, urotensin
group 6a	cholecystokinin, gastrin, neuropeptide FF, orexin
group 6b	gonadotrophin-releasing hormone, vasopressin
group 6c	orphan
group 7a	bombesin, endolethin, gastrin-releasing peptide, neuromedin B
group 7b	growth hormone secretagogue, neuromedin U, neurotensin, motilin, thyrotropin-releasing hormone
group 8a	anaphylatoxin, lipoxin A4, N-formyl peptide
group 8b	mas
group 9a	substance-P, substance K, neuromedin K
group 9b	prolactin-releasing peptide, melatonin, neuropeptide Y, prokineticin
group 10	follitropin, thyrotropin, thyrotropin-releasing hormone, lutropinchoriogonadotropic
group 11	lipids, proprionate, short chain fatty acids, nicotinic acid, ATP
group 12	UDP-glucose, ADP, platelet activating factor
group 13	adrenocorticotropic hormone, cannnabinoid, sphingosine 1-phosphate, lysophosphatidic acid, sphingolipid, melanotropin
group 14	prostaglandin, prostacyclin, thromboxane
group 15	lysophosphatidylcholine, psychosine, thrombin, proteinase
group 16	opsins, peropsin
group 17	5-hydroxytryptamine, adrenergic, dopamine, histamine H2
group 18a	muscarinic acetylocholine, histamine, H2
group 18b	adenosine
groups18c-i	orphan
group 19	5-hydroxytryptamine
