# Supplementary material for: Phylogenomic Analysis Reveals Extensive Phylogenetic Mosaicism in the Human GPCR Superfamily
Source: Evol Bioinform Online. 2007 Sep 26;3:357–70. (PMC2684142)
Supplement: Supplementary alignments [file ebo-03-357-s1.doc]

Group A1

C3X1 -M-----------DQFPESVTENFE-YDDLAEACYIGDIVVFGTVFLSIFYSVIFAIGLVGNLLVVFALTNSKKPKSVTDIYLLNLALSDLLFVATLPFWTHYL-INEKGLHNAMCKFTTAFFFIGFFGSIFFITVISIDRYLAIVL-AANSMNNRTVQHGVTISLGVWAAAILVAAPQFMFTKQKE----NECL----GDYPEVLQEIWPVLRNVETNFLGFLLPLLIMSYCYFRIIQTLFSCKNHKK-AKAIKLILLVVIVFFLFWTPYNVMIFLETLKLYDFFPSCDMRKDLRLALSVTETVAFSHCCLNPLIYAFAGEKFRRYLYHLYGK---CLAVLCGRSVHV-DFSSSESQRSRHGSVLSSN-------FTYHTSDGDALLLL

CKR1 -MET-------PNTTEDYDTTTEFD-YG-DATPCQKVNERAFGAQLLPPLYSLVFVIGLVGNILVVLVLVQYKRLKNMTSIYLLNLAISDLLFLFTLPFWIDYKLKDDWVFGDAMCKILSGFYYTGLYSEIFFIILLTIDRYLAIVH-AVFALRARTVTFGVITSIIIWALAILASMPGLYFSKTQWEFTHHTCS----LHFPHESLREWKLFQALKLNLFGLVLPLLVMIICYTGIIKILLRRPNEKK-SKAVRLIFVIMIIFFLFWTPYNLTILISVFQDFLFTHECEQSRHLDLAVQVTEVIAYTHCCVNPVIYAFVGERFRKYLRQLFHR---RVAVHLVKWLPFLSVDRLERVSST----------------SPSTGEHELSAGF

CKR3 -MTT-------SLDTVETFGTTSYY-DD-VGLLCEKADTRALMAQFVPPLYSLVFTVGLLGNVVVVMILIKYRRLRIMTNIYLLNLAISDLLFLVTLPFWIHYVRGHNWVFGHGMCKLLSGFYHTGLYSEIFFIILLTIDRYLAIVH-AVFALRARTVTFGVITSIVTWGLAVLAALPEFIFYETEELFEETLCS----ALYPEDTVYSWRHFHTLRMTIFCLVLPLLVMAICYTGIIKTLLRCPSKKK-YKAIRLIFVIMAVFFIFWTPYNVAILLSSYQSILFGNDCERSKHLDLVMLVTEVIAYSHCCMNPVIYAFVGERFRKYLRHFFHR---HLLMHLGRYIPFLPSEKLERTSSV----------------SPSTAEPELSIVF

CKR2 MLSTSRSRFIRNTNESGEEVTTFFD-YD-YGAPCHKFDVKQIGAQLLPPLYSLVFIFGFVGNMLVVLILINCKKLKCLTDIYLLNLAISDLLFLITLPLWAHSA-ANEWVFGNAMCKLFTGLYHIGYFGGIFFIILLTIDRYLAIVH-AVFALKARTVTFGVVTSVITWLVAVFASVPGIIFTKCQKEDSVYVCG----PYFPR----GWNNFHTIMRNILGLVLPLLIMVICYSGILKTLLRCRNEKKRHRAVRVIFTIMIVYFLFWTPYNIVILLNTFQEFFGLSNCESTSQLDQATQVTETLGMTHCCINPIIYAFVGEKFRSLFHIALGC---RIAPLQKPVCGGPGVRPGKNVKVT-TQGLLDGRGKGKSIGRAPEASLQDKEGA

CKR5 -MDYQVSSPIY-----------DIN-YY-TSEPCQKINVKQIAARLLPPLYSLVFIFGFVGNMLVILILINCKRLKSMTDIYLLNLAISDLFFLLTVPFWAHYA-AAQWDFGNTMCQLLTGLYFIGFFSGIFFIILLTIDRYLAVVH-AVFALKARTVTFGVVTSVITWVVAVFASLPGIIFTRSQKEGLHYTCS----SHFPYSQYQFWKNFQTLKIVILGLVLPLLVMVICYSGILKTLLRCRNEKKRHRAVRLIFTIMIVYFLFWAPYNIVLLLNTFQEFFGLNNCSSSNRLDQAMQVTETLGMTHCCINPIIYAFVGEKFRNYLLVFFQK---HIAKRFCKCCSIFQQEAPERASSV---------------YTRSTGEQEISVGL

CKR4 ----MNPTDIAD-TTLDESIYSNYYLYESIPKPCTKEGIKAFGELFLPPLYSLVFVFGLLGNSVVVLVLFKYKRLRSMTDVYLLNLAISDLLFVFSLPFWGYYA-ADQWVFGLGLCKMISWMYLVGFYSGIFFVMLMSIDRYLAIVH-AVFSLRARTLTYGVITSLATWSVAVFASLPGFLFSTCYTERNHTYCK----TKYSLNST-TWKVLSSLEINILGLVIPLGIMLFCYSMIIRTLQHCKNEKK-NKAVKMIFAVVVLFLGFWTPYNIVLFLETLVELEVLQDCTFERYLDYAIQATETLAFVHCCLNPIIYFFLGEKFRKYILQLFKTC--RGLFVLCQYCGLLQIYSADTPSSS---------------YTQSTMDHDLHDAL

CKR8 -MDY-------TLDLSVTTVTDYY-YPDIFSSPCDAELIQTNGKLLLAVFYCLLFVFSLLGNSLVILVLVVCKKLRSITDVYLLNLALSDLLFVFSFPFQTYYL-LDQWVFGTVMCKVVSGFYYIGFYSSMFFITLMSVDRYLAVVH-AVYALKVRTIRMGTTLCLAVWLTAIMATIPLLVFYQVASEDGVLQCY----SFYNQQTL-KWKIFTNFKMNILGLLIPFTIFMFCYIKILHQLKRCQNHNK-TKAIRLVLIVVIASLLFWVPFNVVLFLTSLHSMHILDGCSISQQLTYATHVTEIISFTHCCVNPVIYAFVGEKFKKHLSEIFQKSCSQIFNYLGRQMPRE------------SCEKSSSCQQHSSR----SSSVDYIL--

CKRX ----MANYTLAPEDEYDVLIEGELE-SD-EAEQCDKYDAQALSAQLVPSLCSAVFVIGVLDNLLVVLILVKYKGLKRVENIYLLNLAVSNLCFLLTLPFWAHAG-------GDPMCKILIGLYFVGLYSETFFNCLLTVQRYLVFLHKGNFFSARRRVPCGIITSVLAWVTAILATLPEFVVYKPQMEDQKYKCAFSRTPFLPADET-FWKHFLTLKMNISVLVLPLFIFTFLYVQMRKTLRF--REQR-YSLFKLVFAIMVVFLLMWAPYNIAFFLSTFKEHFSLSDCKSSYNLDKSVHITKLIATTHCCINPLLYAFLDGTFSKYLCRCFHL---------------------------RSNTPLQPRGQSAQG--TSREEPDHSTEV

CXC1 -MES-------SGNPE---STTFF-YYDLQSQPCEN-QAWVFATLATTVLYCLVFLLSLVGNSLVLWVLVKYESLESLTNIFILNLCLSDLVFACLLPVWISPY-HWGWVLGDFLCKLLNMIFSISLYSSIFFLTIMTIHRYLSVVS-PLSTLRVPTLRCRVLVTMAVWVASILSSILDTIFHKVLSSGCDY------------SEL-TWYLTSVYQHNLF-FLLSLGIILFCYVEILRTLFRSRSKRR-HRTVKLIFAIVVAYFLSWGPYNFTLFLQTLFRTQIIRSCEAKQQLEYALLICRNLAFSHCCFNPVLYVFVGVKFRTHLKHVLRQ---FWFCRL------QAPS--------PASIPHSPGAFAYEG----------ASFY

GroupA2a

ADMR -----MSVKPSWGPGPSEGVTAVPTSDLGEIHNWTELLDLFNHTLSECHVEL---SQSTKRVVLFALYLA-MFVVGLVENLLVICVNWRG-SGRAGLMNLYILNMAIADLGIVLSLPVWMLE-VTLDYTWLWGSFSCRFTHYFYFVNMYSSIFFLVCLSVDRYVTLTSASPSWQR--YQHRVRRAMCAGIWVLSAIIPLPEVVHIQLVEGPE----PMCLFMAPF-ETYSTWALAVALSTTILGFLLPFPLITVFNVLTACRLRQP-GQPK-----SRRHCLLLCAYVAVFVMCWLPYHVTLLLLTLHGTH-ISLHCHLVHLL--YFFYDVIDCFSMLHCVINPILYNFLSPHFRGRLLNAVVHYLPKDQTKAGTCASSSSCSTQHSIIITKGDSQPAAAAPHPEPSLSFQAHHLLPNTSPISPTQPLTPS

CXCR6b ---------------------------MAEHDYHEDYGFSSFNDSSQEEHQD---FLQFSKVFLPCMYLV-VFVCGLVGNSLVLVISIFY-HKLQSLTDVFLVNLPLADLVFVCTLPFWAYA-GI--HEWVFGQVMCKSLLGIYTINFYTSMLILTCITVDRFIVVVKATKAYNQQAKRMTWGKVTSLLIWVISLLVSLPQIIYGNVFNLDK----LICGY------HDEAISTVVLATQMTLGFFLPLLTMIVCYSVIIKTLLHA-GGFQ-----KHRSLKIIFLVMAVFLLTQMPFNLMKFIRSTHWEYY------AMTSF--HYTIMVTEAIAYLRACLNPVLYAFVSLKFRKNFWKLVKDIGCLPYLGVSHQWKSSEDNSKTFSASHNVEATSMFQL------------------------------

CXCR3b -----MVLEVSDHQVLNDAEVAALLENFSSSYDYGENESDSCCTSPPCPQDF---SLNFDRAFLPALYSL-LFLLGLLGNGAVAAVLLSR-RTALSSTDTFLLHLAVADTLLVLTLPLWAVD-AA--VQWVFGSGLCKVAGALFNINFYAGALLLACISFDRYLNIVHATQLYR--RGPPARVTLTCLAVWGLCLLFALPDFIFLSAHHDERLNAT-HCQYNFP-----QVGRTALRVLQLVAGFLLPLLVMAYCYAHILAVLLVS-RGQR-----RLRAMRLVVVVVVAFALCWTPYHLVVLVDILMDLGALARNCGRESRV--DVAKSVTSGLGYMHCCLNPLLYAFVGVKFRERMWMLLLRLGCPNQRGLQRQPSSSRRDSSWSETSEASYSGL----------------------------------

CXCR4b --------------------MEGISIYTSDNYTEEMGSGDYDSMKEPCFREE---NANFNKIFLPTIYSI-IFLTGIVGNGLVILVMGYQ-KKLRSMTDKYRLHLSVADLLFVITLPFWAVD-AV--ANWYFGNFLCKAVHVIYTVNLYSSVLILAFISLDRYLAIVHATNSQ--RPRKLLAEKVVYVGVWIPALLLTIPDFIFANVSEADD--RY-ICDRFYPN-D---LWVVVFQFQHIMVGLILPGIVILSCYCIIISKLSHS-KGHQ-----KRKALKTTVILILAFFACWLPYYIGISIDSFILLEIIKQGCEFENTV--HKWISITEALAFFHCCLNPILYAFLGAKFKTSAQHALTSVSRGSSLKILSKGKRGGHSSVSTESESSSFHSS----------------------------------

CXCR5b ----------MNYPLTLEMDLENLEDLFWELDRLDNYNDTSLVENHLCPATEGPLMASFKAVFVPVAYSL-IFLLGVIGNVLVLVILERH-RQTRSSTETFLFHLAVADLLLVFILPFAVAE-GS--VGWVLGTFLCKTVIALHKVNFYCSSLLLACIAVDRYLAIVHAVHAYR--HRRLLSIHITCGTIWLVGFLLALPEILFAKVSQGHHNNSLPRCTFSQEN-QAETHAWFTSRFLYHVAGFLLPMLVMGWCYVGVVHRLRQAQRRPQ-----RQKAVRVAILVTSIFFLCWSPYHIVIFLDTLARLKAVDNTCKLNGSL--PVAITMCEFLGLAHCCLNPMLYTFAGVKFRSDLSRLLTKLGCTGPASLCQLFPSWRRSSLSESENATSLTTF----------------------------------

CKR6b ------------MSGESMNFSDVFDSSEDYFVSVNTSYYSVDSEMLLCSLQE---VRQFSRLFVPIAYSL-ICVFGLLGNILVVITFAFY-KKARSMTDVYLLNMAIADILFVLTLPFWAVSHAT--GAWVFSNATCKLLKGIYAINFNCGMLLLTCISMDRYIAIVQATKSFRLRSRTLPRSKIICLVVWGLSVIISSSTFVFNQKYNTQGS--D-VCEPKYQTVSEPIRWKLLMLGLELLFGFFIPLMFMIFCYTFIVKTLVQA-QNSK-----RHKAIRVIIAVVLVFLACQIPHNMVLLVTAANL-GKMNRSCQSEKLI--GYTKTVTEVLAFLHCCLNPVLYAFIGQKFRNYFLKILKDLWCVRRKYKSSGFSCAGRYSENISRQTSETADNDNASSFTM--------------------------

CKR7b MDLGKPMKSVLVVALLVIFQVCLCQDEVTDDYIGDNTTVDYTLFESLCSKKD---VRNFKAWFLPIMYSI-ICFVGLLGNGLVVLTYIYF-KRLKTMTDTYLLNLAVADILFLLTLPFWAYS-AA--KSWVFGVHFCKLIFAIYKMSFFSGMLLLLCISIDRYVAIVQAVSAHRHRARVLLISKLSCVGIWILATVLSIPELLYSDLQRSSSEQAM-RCSLI----TEHVEAFITIQVAQMVIGFLVPLLAMSFCYLVIIRTLLQA-RNFE-----RNKAIKVIIAVVVVFIVFQLPYNGVVLAQTVANFNITSSTCELSKQL--NIAYDVTYSLACVRCCVNPFLYAFIGVKFRNDLFKLFKDLGCLSQEQLRQWSSCRHIRRSSMSVEAETTTTFSP--------------------------------

CKR9b ----------------------MADDYGSESTSSMEDYVNFNFTDFYCEKNN---VRQFASHFLPPLYWL-VFIVGALGNSLVILVYWYC-TRVKTMTDMFLLNLAIADLLFLVTLPFWAIA-AA--DQWKFQTFMCKVVNSMYKMNFYSCVLLIMCISVDRYIAIAQAMRAHTWREKRLLYSKMVCFTIWVLAAALCIPEILYSQIKEESG--IA-ICTMVYPS-DESTKLKSAVLTLKVILGFFLPFVVMACCYTIIIHTLIQA-KKSS-----KHKALKVTITVLTVFVLSQFPYNCILLVQTIDAYAMFISNCAVSTNI--DICFQVTQTIAFFHSCLNPVLYVFVGERFRRDLVKTLKNLGCISQAQWVSFTRREGSLKLSSMLLETTSGALSL--------------------------------

CKRA -----------------MGTEATEQVSWGHYSGDEEDAYSAEPLPELCYKAD---VQAFSRAFQPSVSLT-VAALGLAGNGLVLATHLAARRAARSPTSAHLLQLALADLLLALTLPFAAAG-AL--QGWSLGSATCRTISGLYSASFHAGFLFLACISADRYVAIARALPAG-PRPSTPGRAHLVSVIVWLLSLLLALPALLFSQDGQREG--QR-RCRLIFPE-GLTQTVKGASAVAQVALGFALPLGVMVACYALLGRTLLAA-RGPE-----RRRALRVVVALVAAFVVLQLPYSLALLLDTADLLAARERSCPASKRK--DVALLVTSGLALARCGLNPVLYAFLGLRFRQDLRRLLRGGSSPSGPQPRRGCPRRPRLSSCSAPTETHSLSWDN--------------------------------

CKRB -----------------MALEQNQSTDYYYEENEMNGTYDYSQYELICIKED---VREFAKVFLPVFLTI-VFVIGLAGNSMVVAIYAYY-KKQRTKTDVYILNLAVADLLLLFTLPFWAVN-AV--HGWVLGKIMCKITSALYTLNFVSGMQFLACISIDRYVAVTKVPSQS--GVGK--PCWIICFCVWMAAILLSIPQLVFYTVNDNAR------CIPIFPR-YLGTSMKALIQMLEICIGFVVPFLIMGVCYFITARTLMKM-PNIK-----ISRPLKVLLTVVIVFIVTQLPYNIVKFCRAIDIIYSLITSCNMSKRM--DIAIQVTESIALFHSCLNPILYVFMGASFKNYVMKVAKKYGSWRRQRQSVEEFPFDSEGPTEPTSTFSI-------------------------------------

CML2 ---MDVTSQARGVGLEMYPGTAQPAAPNTTSPELNLSHPLLGTALANGTGELSEHQQYVIGLFLSCLYTIFLFPIGFVGNILILVVNISF-REKMTIPDLYFINLAVADLILVADSLIEVFN-LH--ERYYDIAVLCTFMSLFLQVNMYSSVFFLTWMSFDRYIALARAMRCSLF--RTKHHARLSCGLIWMASVSATLVPFT---AVHLQHTDEACFCFADVRE----------VQWLEVTLGFIVPFAIIGLCYSLIVRVLVRA-HRHRGLRPRRQKALRMILAVVLVFFVCWLPENVFISVHLLQRTQPGAAPCKQSFRHAHPLTGHIVNLAAFSNSCLNPLIYSFLGETFRDKLRLYIEQKTNLPALNRFCHAALKAVIPDSTEQSDVRFSSAV---------------------------------

CXCR1b ------------------MSNITDPQMWDFDDLNFTGMPPADEDYSPCMLE----TETLNKYVVIIAYAL-VFLLSLLGNSLVMLVILYS-RVGRSVTDVYLLNLALADLLFALTLPIWAAS-KV--NGWIFGTFLCKVVSLLKEVNFYSGILLLACISVDRYLAIVHATRTLTQ--KRHL-VKFVCLGCWGLSMNLSLPFFLFRQAYHPNNS--SPVCYEVLGN--DTAKWRMVLRILPHTFGFIVPLFVMLFCYGFTLRTLFKA-HMGQ-----KHRAMRVIFAVVLIFLLCWLPYNLVLLADTLMRTQVIQETCERRNNI--GRALDATEILGFLHSCLNPIIYAFIGQNFRHGFLKILAMHGLVSKEFLARHRVTSYTSSSVNVSSNL---------------------------------------

CXCR2b ---------MEDFNMESDSFEDFWKGEDLSNYSYSSTLPPFLLDAAPCEPE----SLEINKYFVVIIYAL-VFLLSLLGNSLVMLVILYS-RVGRSVTDVYLLNLALADLLFALTLPIWAAS-KV--NGWIFGTFLCKVVSLLKEVNFYSGILLLACISVDRYLAIVHATRTLTQ--KRYL-VKFICLSIWGLSLLLALPVLLFRRTVYSSN--VSPACYEDMGN--NTANWRMLLRILPQSFGFIVPLLIMLFCYGFTLRTLFKA-HMGQ-----KHRAMRVIFAVVLIFLLCWLPYNLVLLADTLMRTQVIQETCERRNHI--DRALDATEILGILHSCLNPLIYAFIGQKFRHGLLKILAIHGLISKDSLPKDSRPSFVGSSSGHTSTTL--------------------------------------

RDC1 --------------MDLHLFDYAEPGNFSDISWPCNSSDCIVVDTVMCPNMP---NKSVLLYTLSFIYIF-IFVIGMIANSVVVWVNIQA-KTTGYDTHCYILNLAIADLWVVLTIPVWVVS-LVQHNQWPMGELTCKVTHLIFSINLFSGIFFLTCMSVDRYLSITYFTNTPSS--RKKMVRRVVCILVWLLAFCVSLPDTYYLKTVTSASNNET-YCRSFYPE-HSIKEWLIGMELVSVVLGFAVPFSIIAVFYFLLARAISAS-SDQE-----KHSSRKIIFSYVVVFLVCWLPYHVAVLLDIFSILHYIPFTCRLEHAL--FTALHVTQCLSLVHCCVNPVLYSFINRNYRYELMKAFIFKYSAKTGLTKLIDASRVSETEYSALEQNAK-------------------------------------

GroupA2b

DUFF_HUMA MGNCLHRAELSPSTENSSQLDFEDVWNSSYGVNDSFPDGDYDANLEAAAPCHSCNLLDDSALPFFILTSVLGILASSTVLFMLFRPLFRWQLCPGWPVLAQLAVGSALFSIVVPVLAPGLGSTRSSALCSLGYCVWYGSAFAQALLLGCHASLGHRLGAGQVPGLTLGLTVGIWGVAALLTLPVTLASGASGGLCTLIYSTELKALQATHTVACLAIFVLLPLGLFGAKGLKKALGMGPGPWMNILWAWFIFWWPHGVVLGLDFLVRSKLLLLSTCLAQQALDLLLNLAEALAILHCVATPLLLALFCHQATRTLLPSLPLPEGWSSHLDTLGSKS

Group A3

AG22_HUMA ------------MKGNSTLATTSKNITSGLHFGLVNISGNNESTLNCSQKPSDKHLDA-IPILYYIIFVIGFLVNIVVV-TLFCCQKGPKKVSSIYIFNLAVADLLLLATLPLWATYYSYRYDWLFGPVMCKVFGSFLTLNMFASIFFITCMSVDRYQSVIYPFLSQR-RNPWQASYIVPLVWCMACLSSLPTFYFRDVRTIE--YLGVNACIMAFPPE----KYAQWSAGIALMKNILGFIIPLIFIATCYFGIRKHLLKTNSYG-KNR---ITRDQVLKMAAAVVLAFIICWLPFHVLTFLDALAWMGVIN-SCEVIAVIDLALPFAILLGFTNSCVNPFLYCFVGNRFQQKLRSVFRVPITWLQGKRESMSCRKSSSLREMETFVS--------------------------

AG2R_HUMA -----------------------------MILNSSTEDGIKRIQDDCPKAGRHNYIFVMIPTLYSIIFVVGIFGNSLVV-IVIYFYMKLKTVASVFLLNLALADLCFLLTLPLWAVYTAMEYRWPFGNYLCKIASASVSFNLYASVFLLTCLSIDRYLAIVHPMKSRLRRTMLVAKVTCIIIWLLAGLASLPAIIHRNVFFIE--NTNITVCAFHYESQ-----NSTLPIGLGLTKNILGFLFPFLIILTSYTLIWKALKKAYEIQ-KNK---PRNDDIFKIIMAIVLFFFFSWIPHQIFTFLDVLIQLGIIR-DCRIADIVDTAMPITICIAYFNNCLNPLFYGFLGKKFKRYFLQLLKYIPPKAKSHSNLSTKMSTLSYRPSDNVSSSTKKPAPCFEVE--------------

AG2S_HUMA -----------------------------MILNSSTEDGIKRIQDDCPKAGRHNYIFVMIPTLYSIIFVVGIFGNSLVV-IVIYFYMKLKTVASVFLLNLALADLCFLLTLPLWAVYTAMEYRWPFGNYLCKIASASVSFNLYASVFLLTCLSIDRYLAIVHPMKSRLRRTMLVAKVTCIIIWLLAGLASLPAIIHRNVFFIE--NTNITVCAFHYES-----RNSTLPIGLGLTKNILGSCFPFLIILTSYTLIWKALKKAYEIQ-KNN---PRNDDIFRIIMAIVLFFFFSWIPHQIFTFLDVLIQQGIIR-DCRIADIVDTAMPITIWIAYFNNCLNPLFYGFLGKKFKKDILQLLKYIPPKAKSHSNLSTKMSTLSYRPSDNVSSSTKKPAPCFEVE--------------

APJ_HUMAN -----------------------------MEEGGDFDNYYGADNQSECEYTDWKSSGALIPAIYMLVFLLGTTGNGLVLWTVFRSSREKRRSADIFIASLAVADLTFVVTLPLWATYTYRDYDWPFGTFFCKLSSYLIFVNMYASVFCLTGLSFDRYLAIVRPVANARLRLRVSGAVATAVLWVLAALLAMPVMVLRTTGDLE--NTTKVQCYMDYSMVATVSSEWAWEVGLGVSSTTVGFVVPFTIMLTCYFFIAQTIAGHFRKERIEG--LRKRRRLLSIIVVLVVTFALCWMPYHLVKTLYMLGSL-LHW-PCDFDLFLMNIFPYCTCISYVNSCLNPFLYAFFDPRFRQACTSMLCCGQSRCAGTSHSSSGEKSASYSSGHSQGPGPNMGKGGEQMHEKSIPYSQETLVVD

BRB1_HUMA --------------------MASSWPPLELQSSNQSQLFPQNATACDNAPEAWDLLHRVLPTFIISICFFGLLGNLFVL-LVFLLPRRQLNVAEIYLANLAASDLVFVLGLPFWAENIWNQFNWPFGALLCRVINGVIKANLFISIFLVVAISQDRYRVLVHPMASGRQQRRRQARVTCVLIWVVGGLLSIPTFLLRSIQAVP--DLNITACILLLPHE-------AWHFARIVELNILGFLLPLAAIVFFNYHILASLRTREEVS-RTRVRGPKDSKTTALILTLVVAFLVCWAPYHFFAFLEFLFQVQAVR-GCFWEDFIDLGLQLANFFAFTNSSLNPVIYVFVGRLFRTKVWELYKQCTPKSLAPISSSHRKEIFQLFWRN------------------------------

BRB2_HUMA MFSPWKISMFLSVREDSVPTTASFSADMLNVTLQGPTLNGTFAQSKCPQVEWLGWLNTIQPPFLWVLFVLATLENIFVL-SVFCLHKSSCTVAEIYLGNLAAADLILACGLPFWAITISNNFDWLFGETLCRVVNAIISMNLYSSICFLMLVSIDRYLALVKTMSMGRMRGVRWAKLYSLVIWGCTLLLSSPMLVFRTMKEYSDEGHNVTACVISYPS-------LIWEVFTNMLLNVVGFLLPLSVITFCTMQIMQVLRNN-EMQKFKE--IQTERRATVLVLVVLLLFIICWLPFQISTFLDTLHRLGILS-SCQDERIIDVITQIASFMAYSNSCLNPLVYVIVGKRFRKKSWEVYQGVCQKGGCRSEPIQMENSMGTLRTSISVERQIHKLQDWAGSRQ------------

GP25_HUMA ------------------MAPTEPWSPSPGSAPWDYSGLDGLEELELCPAGDLPYGYVYIPALYLAAFAVGLLGNAFVV-WLLAGRRGPRRLVDTFVLHLAAADLGFVLTLPLWAAAAAR-RPWPFGDGLCKLSTFALAGTRSAGALLLAGMSVDRYLAVVKLLEARPLRTPRCAVASCCGVWAVALLAGLPSLVYRGLQPLP--GGQDSQCGEEPS--------HAFQ-GLSLLLLLLTFVLPLVVTLFCYCRISRRLRRPPHVGR-------ARRNSLRIIFAIESTFVGSWLPFSALRAVFHLARLGALPLPCPLLLALRWGLTIATCLAFVNSCANPLIYLLLDRSFRARALDGACGRTGRLARRISSASSLSRDDSSVFRCRAQAANTASASW-----------------

GP15_HUMA ------------------------MDPEETSVYLDYYYATSPNSDIRETHSHVPYTSVFLPVFYTAVFLTGVLGNLVLM-GALHFKPGSRRLIDIFIINLAASDFIFLVTLPLWVDKEASLGLWRTGSFLCKGSSYMISVNMHCSVLLLTCMSVDRYLAIVWPVVSRKFRRTDCAYVVCASIWFISCLLGLPTLLSRELTLID--DK--PYCAEK--------KATPIKLIWSLVALIFTFFVPLLSIVTCYCCIARKLCAHYQQSGKHN---KKLKKSIKIIFIVVAAFLVSWLPFNTFKFLAIVSGLRQEHY--LPSAILQLGMEVSGPLAFANSCVNPFIYYIFDSYIRRAIVHCLCPCLKNYDFGSSTETSDSHLTKALSTFIHAEDFARRRKRSVSL-------------

Group A4

GPR7a ---------------MDNASFSEPWPANASG--PDPALSCS-------NASTLAPLPAP-------------LAVAVPVVYAVICAVGLAGNSAVLYVLLRAPRMKTVTNLFILNLAIADELFTLVLPINIADFLLRQWPFGELMCKLIVAIDQYNTFSSLYFLTVMSADRYLVVLATAESRRVAGRTYSAARAVSLAVWGIVTLVVLPFAVFARLD-DEQG-RRQCVLVFPQP-EAFWWRASRLYTLVLGFAIPVSTICVLYTTLLCRLHAMRLDSH---AKALERAKKRVTFLVVAILAVCLLCWTPYHLSTVVALTTDLP-QTPLVIAISYFITSLTYANSCLNPFLYAFLDASFRRNLRQLITCR--------AAA-----------------------------------------------------------------------------------------

GPR8a ---------------MQAAGHPEPLDSRGSFSLPTMGANVSQDNGTGHNATFSEPLPF--------------LYVLLPAVYSGICAVGLTGNTAVILVILRAPKMKTVTNVFILNLAVADGLFTLVLPVNIAEHLLQYWPFGELLCKLVLAVDHYNIFSSIYFLAVMSVDRYLVVLATVRSRHMPWRTYRGAKVASLCVWLGVTVLVLPFFSFAGVYSNELQ-VPSCGLSFPWP-ERVWFKASRVYTLVLGFVLPVCTICVLYTDLLRRLRAVRLRSG---AKALGKARRKVTVLVLVVLAVCLLCWTPFHLASVVALTTDLP-QTPLVISMSYVITSLTYANSCLNPFLYAFLDDNFRKNFRSILRC-----------------------------------------------------------------------------------------------------

OPRD ---------------------MEPAPSAG--AELQPPLFANASDAY-PSACPSAG-ANASGPPGARSASSLALAIAITALYSAVCAVGLLGNVLVMFGIVRYTKMKTATNIYIFNLALADALATSTLPFQSAKYLMETWPFGELLCKAVLSIDYYNMFTSIFTLTMMSVDRYIAVCHPVKA--LDFRTPAKAKLINICIWVLASGVGVPIMVMAVTRPRDGA--VVCMLQFPSP-SWYWDTVTKICVFLFAFVVPILIITVCYGLMLLRLRSVRLLSG---SKEKDRSLRRITRMVLVVVGAFVVCWAPIHIFVIVWTLVDIDRRDPLVVAALHLCIALGYANSSLNPVLYAFLDENFKRCFRQLCRKPCG-------RPDPSSFSRAREATARERVTACTPSDGPGGGAAA---------------------------------------------------------

OPRK ------------MESPIQIFRGEPGPTCAPSACLPPNSSAWFPGWAEPDSNGSAGSEDAQLEP--AHIS-PAIPVIITAVYSVVFVVGLVGNSLVMFVIIRYTKMKTATNIYIFNLALADALVTTTMPFQSTVYLMNSWPFGDVLCKIVISIDYYNMFTSIFTLTMMSVDRYIAVCHPVKA--LDFRTPLKAKIINICIWLLSSSVGISAIVLGGTKVREDVDVIECSLQFPDDDYSWWDLFMKICVFIFAFVIPVLIIIVCYTLMILRLKSVRLLSG---SREKDRNLRRITRLVLVVVAVFVVCWTPIHIFILVEALGSTS-HSTAALSSYYFCIALGYTNSSLNPILYAFLDENFKRCFRDFCFPLKM-------RMERQSTSRVRNTVQDPAYLRDIDGMNKPV-------------------------------------------------------------

OPRM MDSSAAPTNASNCTDALAYSSCSPAPSPGSWVNLSHLDGNLSDPCG-PNRTDLGG-RDSLCPP--TGSPSMITAITIMALYSIVCVVGLFGNFLVMYVIVRYTKMKTATNIYIFNLALADALATSTLPFQSVNYLMGTWPFGTILCKIVISIDYYNMFTSIFTLCTMSVDRYIAVCHPVKA--LDFRTPRNAKIINVCNWILSSAIGLPVMFMATTKYRQGS--IDCTLTFSHP-TWYWENLLKICVFIFAFIMPVLIITVCYGLMILRLKSVRMLSG---SKEKDRNLRRITRMVLVVVAVFIVCWTPIHIYVIIKALVTIP-ETTFQTVSWHFCIALGYTNSCLNPVLYAFLDENFKRCFREFCIPTSS-------NIEQQNSTRIRQNTRDHPSTANTVDRTNHQLENLEAETAPLP-------------------------------------------------

OPRX ----------------MEPLFPAPFWEVIYGSHLQGNLSLLSPNHSLLPPHLLLNASHGAFLP-------LGLKVTIVGLYLAVCVGGLLGNCLVMYVILRHTKMKTATNIYIFNLALADTLVLLTLPFQGTDILLGFWPFGNALCKTVIAIDYYNMFTSTFTLTAMSVDRYVAICHPIRA--LDVRTSSKAQAVNVAIWALASVVGVPVAIMGSAQVEDEE--IECLVEIPTP-QDYWGPVFAICIFLFSFIVPVLVISVCYSLMIRRLRGVRLLSG---SREKDRNLRRITRLVLVVVAVFVGCWTPVQVFVLAQGLGVQP-SSETAVAILRFCTALGYVNSCLNPILYAFLDENFKACFRKFCCASAL-------RRDVQVSDRVRSIAKDVALACKTSETVPRPA------------------------------------------------------------

SSR1 ------------MFPNGTASSPSSSPSPSPGSCGEGGGSRGPGAGAADGMEEPGRNASQNGTL----SEGQGSAILISFIYSVVCLVGLCGNSMVIYVILRYAKMKTATNIYILNLAIADELLMLSVPFLVTSTLLRHWPFGALLCRLVLSVDAVNMFTSIYCLTVLSVDRYVAVVHPIKA--ARYRRPTVAKVVNLGVWVLSLLVILPIVVFSRTAANSDG-TVACNMLMPEP-AQRWLVGFVLYTFLMGFLLPVGAICLCYVLIIAKMRMVALKAG---WQQRKRSERKITLMVMMVVMVFVICWMPFYVVQLVNVFAEQD-D----ATVSQLSVILGYANSCANPILYGFLSDNFKRSFQRILCL-----SWMDNAAEEPVDYYATALKSR--AYSVEDFQPENLESGG--VFRNGTCTSRITTL-----------------------------------------

SSR2 ----------------MDMADEPLNGSHTWLS-IPFDLNGSVVSTNTSNQTEPYYDLTS--------------NAVLTFIYFVVCIIGLCGNTLVIYVILRYAKMKTITNIYILNLAIADELFMLGLPFLAMQVALVHWPFGKAICRVVMTVDGINQFTSIFCLTVMSIDRYLAVVHPIKS--AKWRRPRTAKMITMAVWGVSLLVILPIMIYAGLRSNQWG-RSSCTINWPGE-SGAWYTGFIIYTFILGFLVPLTIICLCYLFIIIKVKSSGIRVG---SSKRKKSEKKVTRMVSIVVAVFIFCWLPFYIFNVSSVSMAIS-PTPALKGMFDFVVVLTYANSCANPILYAFLSDNFKKSFQNVLCLV------KVSGTDDGERSDSKQDKSR------LNETTETQRTLL----NGDLQTSI---------------------------------------------

SSR3 ----------------MDMLHPSSVSTTSEPENASSAWPPDATLGNVSAGPSPAGLAVS--------------GVLIPLVYLVVCVVGLLGNSLVIYVVLRHTASPSVTNVYILNLALADELFMLGLPFLAAQNALSYWPFGSLMCRLVMAVDGINQFTSIFCLTVMSVDRYLAVVHPTRS--ARWRTAPVARTVSAAVWVASAVVVLPVVVFSGVPRGMS----TCHMQWPEP-AAAWRAGFIIYTAALGFFGPLLVICLCYLLIVVKVRSAGRRVWAPSCQRRRRSERRVTRMVVAVVALFVLCWMPFYVLNIVNVVCPLP-EEPAFFGLYFLVVALPYANSCANPILYGFLSYRFKQGFRRVLLRPS-----RRVRSQEPTVGPPEKTEEED----EEEEDGEESREGG----KGKEMNGRVSQITQPGTSGQERPPSRVASKEQQLLPQEASTGEKSSTMRISYL

SSR4 ------------------MSAPSTLP---PGGE-EGLGTAWPSAANASSAPAEAEEAVAGPGD----ARAAG-MVAIQCIYALVCLVGLVGNALVIFVILRYAKMKTATNIYLLNLAVADELFMLSVPFVASSAALRHWPFGSVLCRAVLSVDGLNMFTSVFCLTVLSVDRYVAVVHPLRA--ATYRRPSVAKLINLGVWLASLLVTLPIAIFADTRPARGGQAVACNLQWPHP-A--WSAVFVVYTFLLGFLLPVLAIGLCYLLIVGKMRAVALRAG---WQQRRRSEKKITRLVLMVVVVFVLCWMPFYVVQLLNLVVTSL-D----ATVNHVSLILSYANSCANPILYGFLSDNFRRSFQRVLCLRCCLLEGAGGAEEEPLDYYATALKSKGGAGCMCPPLPCQQEALQPEPGRKRIPLTRTTTF-----------------------------------------

SSR5 ----------------MEPLFPASTPSWNAS---SPGAASGGGDNRTLVGPAPSAGAR---------------AVLVPVLYLLVCAAGLGGNTLVIYVVLRFAKMKTVTNIYILNLAVADVLYMLGLPFLATQNAASFWPFGPVLCRLVMTLDGVNQFTSVFCLTVMSVDRYLAVVHPLSS--ARWRRPRVAKLASAAAWVLSLCMSLPLLVFADVQEGG-----TCNASWPEP-VGLWGAVFIIYTAVLGFFAPLLVICLCYLLIVVKVRAAGVRVG---CVRRR-SERKVTRMVLVVVLVFAGCWLPFFTVNIVNLAVALP-QEPASAGLYFFVVILSYANSCANPVLYGFLSDNFRQSFQKVLCLR------KGSGAKDADATEPRPDRIRQ----QQEATPPAHRAAA----NGLMQTSKL--------------------------------------------

Group A5

GALR_HUMA --------------------------------------------------------MELAVGNLSEGNASCPEPPAPEPGPLFGIGVENF----VTLVVFGLIFALGVLGNSLVITVLARSKP---GKPRSTTNLFILNLSIADLAYLLFCIPFQAT-VYALP-TWVLGAFICKFIHYFFTVSMLVSIFTLAAMSVDRYVAIVHSRRSSSLRVSRN---------------ALLGVGCIWALSIAMASPVAYHQGLFHPRA-SNQTFCWEQWPDP--RHKKAY--VVC-----TFVFGYLLPLLLICFCYAKVLNHLHKKLK-------------NMSKKS-EASKKKTAQTVLVVVVVFGISWLPHHIIHLW---AEFGV----FPLTPA--------SFLFRI----TAHCLAYSNSSVNPIIYAFLSENFRKAYKQVFKCHIRKDSHLSDTKENKSRIDTPPSTNCTHV-------------------------------------------------

GALS_HUMA ----------------------------------------------------------------MNVSGCPGAGNASQAGGGGGWHPEAV----IVPLLFALIFLVGTVGNTLVLAVLLRGGQAV-----STTNLFILNLGVADLCFILCCVPFQAT-IYTLD-GWVFGSLLCKAVHFLIFLTMHASSFTLAAVSLDRYLAIRYPLHSRELRTPRN---------------ALAAIGLIWGLSLLFSGPYLSYYRQS--QL-ANLTVCHPAWSAP--R-RRAM--DIC-----TFVFSYLLPVLVLGLTYARTLRYLWRA--VDPV--AAGSG-------A-RRAKRKVTRMILIVAALFCLCWMPHHALILC---VWFGQ----FPLTRA--------TYALRI----LSHLVSYANSCVNPIVYALVSKHFRKGFRTICAGLLGRAPGRASGRVCAAARGTHSGSVLERESSDLLHMSEAAGALRPCPGASQPCILEPCPGPSWQGPKAGDSILTVDVA

GALT_HUMA ------------------------------------------------------------------------MADAQNISLDSPGSVGAV----AVPVVFALIFLLGTVGNGLVLAVLLQPGPSAWQEPGSTTDLFILNLAVADLCFILCCVPFQAT-IYTLD-AWLFGALVCKAVHLLIYLTMYASSFTLAAVSVDRYLAVRHPLRSRALRTPRN---------------ARAAVGLVWLLAALFSAPYLSYYGTV--RY-GALELCVPAWEDA--R-RRAL--DVA-----TFAAGYLLPVAVVSLAYGRTLRFLWAA--VGPAGAAAAEA-------R-RRATGRAGRAMLAVAALYALCWGPHHALILC---FWYGR----FAFSPA--------TYACRL----ASHCLAYANSCLNPLVYALASRHFRARFRRLWPCGRRRRHRARRALRRVRPASSGPPGCPGDARPSGRLLAGGGQGPEPREGPVHGGEAARGPE------------------

GPR54 -----------------------------------------------MHTVATSGPNASWGAPANASGCPGCGANASDGPVPSPRAVDAW----LVPLFFAALMLLGLVGNSLVIYVICRHKP-----MRTVTNFYIANLAATDVTFLLCCVPFTAL-LYPLP-GWVLGDFMCKFVNYIQQVSVQATCATLTAMSVDRWYVTVFPLRALHRRTPRL---------------ALAVSLSIWVGSAAVSAPVLALHRLS----PGPRAYCSEAFPSR--ALERAF--ALY-----NLLALYLLPLLATCACYAAMLRHLGR-VAVRPAPADSALQGQVLAERA-GAVRAKVSRLVAAVVLLFAACWGPIQLFLVL---QALGPAGSWHPRSYA--------AYALKT----WAHCMSYSNSALNPLLYAFLGSHFRQAFRRVCPCAPRRPRRPRRPGPSDPAAPHAELHRLGSHPAPARAQKPGSSGLAARGLCVLGEDNAPL--------------------

GPO MLCPSKTDGSGHSGRIHQETHGEGKRDKISNSEGRENGGRGFQMNGGSLEAEHASRMSVLRAKPMSNSQRLLLLSPGSPPRTGSISYINI----IMPSVFGTICLLGIIGNSTVIFAVVKKSKLH--WCNNVPDIFIINLSVVDLLFLL-GMPFMIHQLMGNG-VWHFGETMCTLITAMDANSQFTSTYILTAMAIDRYLATVHPISSTKFRKPSV---------------ATLVICLLWALSFISITPVWLYARLI--PFPGGAVGCGIRLPNP--D-TDLYWFTLY-----QFFLAFALPFVVITAAYVRILQRMTSSVA--PA-----------SQRSIRLRTKRVTRTAIAICLVFFVCWAPYYVLQLT---QLSIS----RPTLTF---------VYLYN----AAISLGYANSCLNPFVYIVLCETFRKRLVLSVKPAAQGQLRAVSNAQTADEERTESKGT-----------------------------------------------------

P2Y7 ----------------------------------------------------------------------MNTTSSAAPPSLG-VEFISL----LAIILLSVALAVGLPGNSFVVWSILKRMQK-----RSVTALMVLNLALADLAVLL-TAPFFLH-FLAQG-TWSFGLAGCRLCHYVCGVSMYASVLLITAMSLDRSLAVARPFVSQKLRTKAM---------------ARRVLAGIWVLSFLLATPVLAYRTVV--PWKTNMSLCFPRYPSE--G-HRAF-HLIF-----EAVTGFLLPFLAVVASYSDIGRRLQAR---------------------RFRRSRRTGRLVVLIILTFAAFWLPYHVVNLA---EAGRA----LAGQAAGLGLVGKRLSLARN----VLIALAFLSSSVNPVLYACAGG-------GLLRSAGVGFVAKLLEGTGSEASSTRRGGSLGQTARSGPAALEPGPSESLTASSPLKLNELN---------------------

SALPR --------MQMADAATIATMNKAAGGDKLAELFSLVPDLLEAANTSGNASLQLPDLWWELGLELPDGAPPGHPPGSGGAESADTEARVRI----LISVVYWVVCALGLAGNLLVLYLMKSMQ----GWRKSSINLFVTNLALTDFQFVL-TLPFWAV-ENALDFKWPFGKAMCKIVSMVTSMNMYASVFFLTAMSVTRYHSVASALKSHRTRGHGRGDCCGRSLGDSCCFSAKALCVWIWALAALASLPSAIFSTTV--KV-MGEELCLVRFPDKLLGRDRQF--WLGLYHSQKVLLGFVLPLGIIILCYLLLVRFIADRRAAGTKGGAAVAG--GRPTGASARRLSKVTKSVTIVVLSFFLCWLPNQALTTWSILIKFNA----VPFSQE--------YFLCQVYAFPVSVCLAHSNSCLNPVLYCLVRREFRKALKSLLWRIASPSITSMRPFTATTKPEHEDQGLQAPAPPHAAAEPDLLYYPPGVVVYSGGRYDLLPSSSAY---------------

UR2R_HUMA ------------------------------------------MALTPESPSSFPGLAATGSSVPEPPGGPNATLNSSWASPTEPSSLEDLVATGTIGTLLSAMGVVGVVGNAYTLVVTCRSLRAV-----ASMYVYVVNLALADLLYLL-SIPFIVA-TYVTK-EWHFGDVGCRVLFGLDFLTMHASIFTLTVMSSERYAAVLRPLDTVQRPKGYR----------------KLLALGTWLLALLLTLPVMLAMRLV--RR-GPKSLCLPAWGPR---AHRAY--LTL-----LFATSIAGPGLLIGLLYARLARAYRRSQ------RASFKR-------A-RRPGARALRLVLGIVLLFWACFLPFWLWQLL---AQYHQ----APLAPR-------TARIVNY----LTTCLTYGNSCANPFLYTLLTRNYRDHLRGRVRGPGSGGGRGPVPSLQPRARFQRCSGRSLSSCSPQPTDSLVLAPAAPARPAPEGPRAPA---------------------

Group A6a

FF1 --------------------------------------------------------------------------------------------------------MEGEPSQPPNSSWPLSQNGTNTEATPATNLTFSSYYQH----TSPVAAMFIVAYALIFLLCMVGNTLVCFIVLKNRHMHTVTNMFILNLAVSDLLVGIFCMPTTLVDNLITGWPFDNATCKMSGLVQGMSVSASVFTLVAIAVERFRCIVHPFREK--LTLRKALVTIAVIWALALLIMCPSAVTLTVTR-EEHHFMVDARNRSYPLYSCWEAWPEKGMRRVYTTVLFSHIYLAPLALIVVMYARIARKLCQA-------------------------------------------------------------------PGPAPGGEEAADPRASRRRARVVHMLVMVALFFTLSWLPLWALLLLIDYGQLSAPQLHLVTVYA-FPFAHWLAFFNSSANPIIYGYFNENFRRGFQAAFRARLCPRPSGSHKEAYSERPGGLLHRRVFVVVRPSDSGLPSESGPSSGAPRPGRLPLRNGRVAHHGLPREGPGCSHLPLTIPAWDI

FF2 MNSFFGTPAASWCLLESDVSSAPDKEAGRERRALSVQQRGGPAWSGSLEWSRQSAGDRRRLGLSRQTAKSSWSRSRDRTCCCRRAWWILVPAADRARRERFIMNEKWDTNSSENWHPIWNVNDTKHHLYSDINITYVNYYLH----QPQVAAIFIISYFLIFFLCMMGNTVVCFIVMRNKHMHTVTNLFILNLAISDLLVGIFCMPITLLDNIIAGWPFGNTMCKISGLVQGISVAASVFTLVAIAVDRFQCVVYPFKPK--LTIKTAFVIIMIIWVLAITIMSPSAVMLHVQEEKYYRVRLNSQNKTSPVYWCREDWPNQEMRKIYTTVLFANIYLAPLSLIVIMYGRIGISLFRAAVPHTGRK------------------------------------------------------------------NQEQWHVVSRKKQKIIKMLLIVALLFILSWLPLWTLMMLSDYADLSPNELQIINIYI-YPFAHWLAFGNSSVNPIIYGFFNENFRRGFQEAFQLQLCQKRAKPMEAYALKAKSHVLINTSNQLVQESTFQNPHGETLLYRKSAEKPQQELVMEELKETTNSSEI--------------

GASR_HUMA -------------------------------------------------------------------------------------------------MELLKLNRSVQGTGPGPGASLCRPGAPLLNSSSVGNLSCEPPRIRGAGTRELELAIRITLYAVIFLMSVGGNMLIIVVLGLSRRLRTVTNAFLLSLAVSDLLLAVACMPFTLLPNLMGTFIFGTVICKAVSYLMGVSVSVSTLSLVAIALERYSAICRPLQARVWQTRSHAARVIVATWLLSGLLMVPYPVYTVVQP------------VGPRVLQCVHRWPSARVRQTWSVLLLLLLFFIPGVVMAVAYGLISRELYLGLRFDGDSDSDSQSRVRNQGGLPGAVHQNGRCRPETGAVGEDSDGCYVQLPRSRPALELTAL------TAPGPGSGSRPTQAKLLAKKRVVRMLLVIVVLFFLCWLPVYSANTWRAFDG---PGAHRALSGAPISFIHLLSYASACVNPLVYCFMHRRFRQACLETCARCCPRPPRARPRALPDEDPPTPSIASLSRLSYTTISTLGPG--------------------------------------------

GPR103 ------------------------------------------MICCSALSPRIHLSFHRSLTGIVLANSSLDIVLHDTYYVVAHCGGNVRRLHCGGPASRERTAMQALNITPEQFSRLLRDHNLT--REQFIALYRLRPLVYTPELPGRAKLALVLTGVLIFALALFGNALVFYVVTRSKAMRTVTNIFICSLALSDLLITFFCIPVTMLQNISDNWLGGAFICKMVPFVQSTAVVTEILTMTCIAVERHQGLVHPFKMKWQYTNRRAFTMLGVVWLVAVIVGSPMWHVQQLE-------IKYDFLYEKEHICCLEEWTSPVHQKIYTTFIL----------------------------------------------------------------------------------------------------SSSSSCLLWKKKRAVIMMVTVVALFAVCWAPFHVVHMMIEYSNFEKEYDD-VTIKMIFAIVQIIGFSNSICNPIVYAFMNENFKKNVLSAVCYCIVNKTFSPAQRHGNSGITMMRKKAKFSLRENPVEETKGEAFSDGNIEVKLCEQTEEKKKLKRHLALFRSELAENSPLDSGH--

OX1R_HUMA -----------------------------------------------------------------------------------------------------MEPSATPGAQMGVPPGSREPSPVPPDYEDEFLRYLWRDYLY----PKQYEWVLIAAYVAVFVVALVGNTLVCLAVWRNHHMRTVTNYFIVNLSLADVLVTAICLPASLLVDITESWLFGHALCKVIPYLQAVSVSVAVLTLSFIALDRWYAICHPLLFK--STARRARGSILGIWAVSLAIMVPQAAVMECSS-----VLPELANRTRLFSVCDERWADDLYPKIYHSCFFIVTYLAPLGLMAMAYFQIFRKLWG-------------RQ-----------------IPGTTSALVRNWKRPSDQLGDLEQGLSGEPQPR--------GRAFLAEVKQMRARRKTAKMLMVVLLVFALCYLPISVLNVLKRVFGMFRQASDREAVYACFTFSHWLVYANSAANPIIYNFLSGKFREQFKAAFSCCLPGLGPCGSLKAPSPRSSASHKSLSLQSRCSISKISEHVVLTSVTTVLP----------------------------------

OX2R_HUMA ---------------------------------------------------------------------------------------------MSGTKLEDSPPCRNWSSASELNETQEPFLNPTDYDDEEFLRYLWREYLH----PKEYEWVLIAGYIIVFVVALIGNVLVCVAVWKNHHMRTVTNYFIVNLSLADVLVTITCLPATLVVDITETWFFGQSLCKVIPYLQTVSVSVSVLTLSCIALDRWYAICHPLMFK--STAKRARNSIVIIWIVSCIIMIPQAIVMECST-----VFPGLANKTTLFTVCDERWGGEIYPKMYHICFFLVTYMAPLCLMVLAYLQIFRKLWC-------------RQ-----------------IPGTSSVVQRKWKPLQPVSQPRGPGQPTKSRM----------SAVAAEIKQIRARRKTARMLMVVLLVFAICYLPISILNVLKRVFGMFAHTEDRETVYAWFTFSHWLVYANSAANPIIYNFLSGKFREEFKAAFSCCCLGVHHRQEDRLTRGRTSTESRKSLTTQISNFDNISKLSEQVVLTSISTLPAANGAGPLQNW---------------------

CCKR_HUMA --------------------------------------------------------------------------------------------------------------MDVVDSLLVNGSNITPPCELGLENETLFCLDQPRPSKEWQPAVQILLYSLIFLLSVLGNTLVITVLIRNKRMRTVTNIFLLSLAVSDLMLCLFCMPFNLIPNLLKDFIFGSAVCKTTTYFMGTSVSVSTFNLVAISLERYGAICKPLQSRVWQTKSHALKVIAATWCLSFTIMTPYPIYS--------NLVPFTKNNNQTANMCRFLLPNDVMQQSWHTFLLLILFLIPGIVMMVAYGLISLELYQGIKFEASQKKSAKERK-----------------PSTTSSGKYEDSDGCYLQKTRPPRKLELRQLSTGSSSRANRIRSNSSAANLMAKKRVIRMLIVIVVLFFLCWMPIFSANAWRAYD---TASAERRLSGTPISFILLLSYTSSCVNPIIYCFMNKRFRLGFMATFPCCPNPGPPGARGEVGEEEEGGTTGASLSRFSYSHMSASVPPQ-------------------------------------------

Group A6b

GRHR_HUMA ----------------MANSASPEQNQNHCSAINNSIPLMQGNLPTLTLSGKIRVTVTFFLFLLSATFNASFLLKLQKWTQKKEKGKKL--SRMKLLLKHLTLANLLETLIVMPLDGMWNITVQWYAGELLCKVLSYLKLFSMYAPAFMMVVISLDRSLAITRPL-ALKSNS-KVGQSMVGLAWILSSVFAGPQLYIFRMIHLADSSGQTKVFSQCVTHCSFSQWWHQAFYNFFTFSCLFIIPLFIMLICNAKIIFTLTRVLHQDP-HELQ-------------------------LNQSKNNIPRARLKTLKMTVAFATSFTVCWTPYYVLGIWYWFDPEMLNRLSD-PVNHFFFLFAFLNPCFDPLIYGYFSL---------------------------------------------------------------------------------

V1AR_HUMA MRLSAGPDAGPSGNSSPWWPLATGAGNTSREAEALGEGNGPPRDVRNEELAKLEIAVLAVTFAVAVLGNSSVLLALHR------TPRKT--SRMHLFIRHLSLADLAVAFFQVLPQMCWDITYRFRGPDWLCRVVKHLQVFGMFASAYMLVVMTADRYIAVCHPLKTLQQP-ARRSRLMIAAAWVLSFVLSTPQYFVFSMIEVNNVTKARDCWA------TFIQPWGSRAYVTWMTGGIFVAPVVILGTCYGFICYNIWCNVRGKT-ASRQSKGAEQAGVAFQKGFLLA-----PC-VSSVKSISRAKIRTVKMTFVIVTAYIVCWAPFFIIQMWSVWDPMSVWTESENPTITITALLGSLNSCCNPWIYMFFSGHLLQDCVQSFPCCQNMKEKFNKEDTDSMSRRQTFYSNNRSPTNSTGMWKDSPKSSKSIKFIPVST----------------

V1BR_HUMA -----------------MDSGPLWDANPTPRGTLSAPNATTPWLGRDEELAKVEIGVLATVLVLATGGNLAVLLTLGQ------LGRKR--SRMHLFVLHLALTDLAVALFQVLPQLLWDITYRFQGPDLLCRAVKYLQVLSMFASTYMLLAMTLDRYLAVCHPLRSLQQP-GQSTYLLIAAPWLLAAIFSLPQVFIFSLREVIQGSGVLDCWA------DFGFPWGPRAYLTWTTLAIFVLPVTMLTACYSLICHEICKNLKVKTQAWRVGGGGWRTWDRPSPSTLAATTRGLPSRVSSINTISRAKIRTVKMTFVIVLAYIACWAPFFSVQMWSVWDKNAPDEDSTNVAFTISMLLGNLNSCCNPWIYMGFNSHLLPRPLRHLACCGGPQPRMRRRLSDGSLSSRHTTLLTRSSCPATLSLSLSLTLSGRPRPEESPRDLELADGEGTAETIIF

V2R_HUMAN --------------MLMASTTSAVPGHPSLPSLPSNSSQERPLDTRDPLLARAELALLSIVFVAVALSNGLVLAALAR------RGRRGHWAPIHVFIGHLCLADLAVALFQVLPQLAWKATDRFRGPDALCRAVKYLQMVGMYASSYMILAMTLDRHRAICRPMLAYRHGSGAHWNRPVLVAWAFSLLLSLPQLFIFAQRNVEGGSGVTDCWA------CFAEPWGRRTYVTWIALMVFVAPTLGIAACQVLIFREIHASL-VPGPSERP-------GGRRRGRRTGS-----PG---EGAHVSAAVAKTVRMTLVIVVVYVLCWAPFFLVQLWAAWDPEAPLE---GAPFVLLMLLASLNSCTNPWIYASFSSSV-SSELRSLLCCARGRTPPSLGPQDESCTTASSSLAKDTSS---------------------------------------

Group A6c

GP22_HUMA MCFSPILEINMQSESNITVRDDIDDINTNMYQPLSYPLSFQVSLTGFLMLEIVLGLGSNLTVLVLYCMKSNLINSVSNIITMNLHVLDVIICVGCIPLTIVILLLSLESNTALICCFHEACVSFASVSTAINVFAITLDRYDISVKPANRILTMGRAVMLMISIWIFSFFSFLIPFIEVNFFSLQSGNTWENKTLLCVSTNEYYTELGMYYHLLVQIPIFFFTVVVMLITYTKILQALNIRIGTRFSTGQKKKARKKKTISLTTQHEATDMSQSSGGRNVVFGVRTSVSVIIALRRAVKRHRERRERQKRVFRMSLLIISTFLLCWTPISVLNTTILCLGPSDLLVKLRLCFLVMAYGTTIFHPLLYAFTRQKFQKVLKSKMKKRVVSIVEADPLPNNAVIHNSWIDPKRNKKITFEDSEIREKRLVPQVVTD

Group A7a

BRS3_HUMA ----------------------------------------------------------------------------------------------------------------------------------------------------------------------------------------------------------------------MAQRQPHSPNQTLISITNDTESSSSVVSNDNTNKGWSGDNSPGIEALCAIYITYAVIISVGILGNAILIKVFFKTKSMQTVPNIFITSLAFGDLLLLLTCVPVDATHYLAEGWL-----FGRIGCKVLSFIRLTSVGVSVFTLTILSADRYKAVVKPL-ERQPSNAILKTCVKAGCVWIVSMIFALPEAIFSNVYTFRDPNKN-MTFESCTSYP-----VSKKL----LQEIHSLLCFLVFYIIPLSIISVYYSLIARTLYKSTLN-IPTEEQSHARKQIESRKRIARTVLVLVALFALCWLPNHLLYLYHSFTSQTYVDPSAMHFI-----FTIFSRVLAFSNSCVNPFALYWLSKSFQKHFKAQLFCCKAERPEPPVADTSLTTLAVMGTVPGTGSIQMSEISVTSFTGCSVKQAEDRF

ET1R_HUMA -------------------------------------------------------------------------------------------------------------------------------------------------------------------------------------METLCLRASFWLALVGCVISDNPERYSTNLSNHVDDFTTFRGTELSFLVTTHQPTNLVLPSNGSMHNYCPQQTKITSAFKYINTVISCTIFIVGMVGNATLLRIIYQNKCMRNGPNALIASLALGDLIYVVIDLPINVFKLLAGRWPFDHNDFGVFLCKLFPFLQKSSVGITVLNLCALSVDRYRAVASWS-RVQGIGIPLVTAIEIVSIWILSFILAIPEAIGFVMVPFEY--RG-EQHKTCMLNA-----TSK--FMEFYQDVKDWWLFGFYFCMPLVCTAIFYTLMTCEMLNRRNGSLRIALSEH----LKQRREVAKTVFCLVVIFALCWFPLHLSRILKKT-VYNEMDKNRCELLSFLLLMDYIGINLATMNSCINPIALYFVSKKFKNCFQSCLCCCCYQSKSLMTSVPMNGTSIQWKNHDQNNHNTDRSSHKDSMN-----------

ETBR_HUMA ----------------------------------------------------------------------------------------------------------------------------------------------------------------MQPPPSLCGRALVALVLACGLSRIWGEERGFPPDRATPLLQTAEIMTPPTKTLWPKGSNASLARSLAPAEVPKGDRTAGSPPRTISPPPCQGPIEIKETFKYINTVVSCLVFVLGIIGNSTLLRIIYKNKCMRNGPNILIASLALGDLLHIVIDIPINVYKLLAEDWP-----FGAEMCKLVPFIQKASVGITVLSLCALSIDRYRAVASWS-RIKGIGVPKWTAVEIVLIWVVSVVLAVPEAIGFDIITMDY--KG-SYLRICLLHP-----VQKTAFMQFYKTAKDWWLFSFYFCLPLAITAFFYTLMTCEMLRKKSG-MQIALNDH----LKQRREVAKTVFCLVLVFALCWLPLHLSRILKLT-LYNQNDPNRCELLSFLLVLDYIGINMASLNSCINPIALYLVSKRFKNCFKSCLCCWC-QSFEEKQSLEEKQSCLKFKANDHGYDNFRSSNKYSSS------------

ETB2_HUMA -----------------------------------------------------------------------------------------------------------------------------------MRWLWPLAVSLAVILAVGLSRVSGGAPLHLGRHRAETQEQQSRSKRGTEDEEAKGVQQYVPEEWAEYPRPIHPAGLQPTKPLVATSPNPDKDGGTPDSGQELRGNLTGAPGQRLQIQNPLYPVTESSYSAYAIMLLALVVFAVGIVGNLSVMCIVWHSYYLKSAWNSILASLALWDFLVLFFCLPIVIFNEITKQRL-----LGDVSCRAVPFMEVSSLGVTTFSLCALGIDRFHVATSTLPKVRPIERCQSILAKLAVIWVGSMTLAVPELLLWQLAQEPAPTMGT--LDSCIMKPSASLPESLYSLVMTYQNARMWWYFGCYFCLPILFT-VTCQLVTWRVRGPPGRKSEC----RASKHEQCESQLNSTVVGLTVVYAFCTLPENVCNIVVAY-LSTELTRQTLDLL------GLINQFSTFFKGAITPVLLLCICRPLGQAFLDCCCCCCCEECGGASEASAANGSDNKLKTEVSSSIYFHKPRESPPLLPLGTPC----

GP37_HUMA MRAPGALLARMSRLLLLLLLKVSASSALGVAPASRNETCLGESCAPTVIQRRGRDAWGPGNSARDVLRARAPREEQGAAFLAGPSWDLPAAPGRDPAAGRGAEASAAGPPGPPTRPPGPWRWKGARGQEPSETLGRGNPTALQLFLQISEEEEKGPRGAGISGRSQEQSVKTVPGASDLFYWPRRAGKLQGSHHKPLSKTANGLAGHEGWTIALPGRALAQNGSLGEGIHEPGGPRRGNSTNRRVRLKNPFYPLTQESYGAYAVMCLSVVIFGTGIIGNLAVMCIVCHNYYMRSISNSLLANLAFWDFLIIFFCLPLVIFHELTKKWL-----LEDFSCKIVPYIEVASLGVTTFTLCALCIDRFRAATNVQMYYEMIENCSSTTAKLAVIWVGALLLALPEVVLRQLSKEDLGFSGRAPAERCIIKISPDLPDTIYVLALTYDSARLWWYFGCYFCLPTLFT-ITCSLVTARKIRKAEKAC----TRGNKRQIQLESQMNCTVVALTILYGFCIIPENICNIVTAY-MATGVSQQTMDLL------NIISQFLLFFKSCVTPVLLFCLCKPFSRAFMECCCCC-CEECIQKSSTVTSDDNDNEYTTELELSPFSTIRREMSTFASVGTHC----

GRPR_HUMA -----------------------------------------------------------------------------------------------------------------------------------------------------------------------------------------------------------------------------MALNDCFLLNLEVDHFMHCNISSHSADLPVNDDWSHPGILYVIPAVYGVIILIGLIGNITLIKIFCTVKSMRNVPNLFISSLALGDLLLLITCAPVDASRYLADRWL-----FGRIGCKLIPFIQLTSVGVSVFTLTALSADRYKAIVRPM-DIQASHALMKICLKAAFIWIISMLLAIPEAVFSDLHPFHEESTN-QTFISCAPYP-----HSNEL----HPKIHSMASFLVFYVIPLSIISVYYYFIAKNLIQSAYN-LPVEGNIHVKKQIESRKRLAKTVLVFVGLFAFCWLPNHVIYLYRSY-HYSEVDTSMLHFV-----TSICARLLAFTNSCVNPFALYLLSKSFRKQFNTQLLCCQPGLIIRSHSTGRSTTCMTSLKSTNPSVATFSLINGNICHERYV-------

NMBR_HUMA --------------------------------------------------------------------------------------------------------------------------------------------------------------------------------------------------------------------------MPSKSLSNLSVTTGANESGSVPEGWERDFLPASDGTTTELVIRCVIPSLYLLIITVGLLGNIMLVKIFITNSAMRSVPNIFISNLAAGDLLLLLTCVPVDASRYFFDEWM-----FGKVGCKLIPVIQLTSVGVSVFTLTALSADRYRAIVNPM-DMQTSGALLRTCVKAMGIWVVSVLLAVPEAVFSEVARISSLD-N-SSFTACIPYP-----QTDEL----HPKIHSVLIFLVYFLIPLAIISIYYYHIAKTLIKSAHN-LPGEYNEHTKKQMETRKRLAKIVLVFVGCFIFCWFPNHILYMYRSF-NYNEIDPSLGHMI-----VTLVARVLSFGNSCVNPFALYLLSESFRRHFNSQLCCGRKSYQERGTSYLLSSSAVRMTSLKSNAKNMVTNSVLLNGHSMKQEMAM---

Group A7b

GHSR_HUMA -----------------MWNATPSEEPGFNLTLADLDWDASPGNDSLGDELLQLFPAPL---LAGVTATCVALFVVGIAGNLLTMLVVSRFRE---LRTTTNLYLSSMAFSDLLIFL--CMPLDLVR-LWQYRPWNFGDLLCKLFQFVSESCTYATVLTITALSVERYFAICFPLRAKVVVTKGRVKLVIFVIWAVAFCSAGPIFVLVGVEHEN-GTDPWD------------------------------------------TNECRPTEFAVRSGLLTV-MVWVSSI-FFFLPVFCLTVLYSLIGRKLWRRRRGD-----------------AVVGA-----------------------SLRDQNHKQTVKMLAVVVFAFILCWLPFHVGRYLFSKSFEP-GSLEIAQISQYCNLVSFVLFYLSAAINPILYNIMSKKYRVAVFRLLGFEPFSQRKLSTLKDESSRAWTESSINT------------------------------------------------------------------

TLR_HUMA ----------------------MGSPWNGSDGPEGAREPPWPALPPCDERRCSPFPLGA---LVPVTAVCLCLFVVGVSGNVVTVMLIGRYRD---MRTTTNLYLGSMAVSDLLILL--GLPFDLYR-LWRSRPWVFGPLLCRLSLYVGEGCTYATLLHMTALSVERYLAICRPLRARVLVTRRRVRALIAVLWAVALLSAGPFLFLVGVEQD---PGISVVPGLNGTARIASSPLASSPPLWLSRAPPPSPPSGPETAEAAALFSRECRPSPAQLGALR-VMLWVTTA-YFFLPFLCLSILYGLIGRELWSSRRPL-----------------RGPAA-----------------------SGRERGHRQTVRVLLVVVLAFIICWLPFHVGRIIYINTEDS----RMMYFSQYFNIVALQLFYLSASINPILYNLISKKYRAAAFKLLLARKSRPRGFHRSRDTAGEVAGDTGGDTVGYTETSANVKTMG----------------------------------------------------

GP39_HUMA ---------------------------------MASPSLPGSDCSQIIDHSHVPEFEVATWIKITLILVYLIIFVMGLLGNSATIRVTQVLQKKGYLQKEVTDHMVSLACSDILVFLI-GMPMEFYSIIWNPLTTSSYTLSCKLHTFLFEACSYATLLHVLTLSFERYIAICHPFRYKAVSGPCQVKLLIGFVWVTSALVALPLLFAMGTEYPLVNVPSHRGLTCN--------------------------RSSTRHHEQPETSNMSICTNLSSRWT----VFQSSIFGAFVVYLVVLLSVAFMCWNMMQVLMKSQKGSLAGGTRPPQLRKSESEES---------------------------RTARRQTIIFLRLIVVTLAVCWMPNQIRRIMAAAKPKHDWTRSYFRAYMILLPFSETFFYLSSVINPLLYTVSSQQFRRVFVQVLCCRLSLQHANHEKRLRVHAHSTTDSARFVQRPLLFASRRQSSARRTEKIFLSTFQSEAEPQSKSQSLSLESLEPNSGAKPANSAAENGFQEHEV

NMU1R ---MTPLCLNCSVLPGDLYPGGARNPMACNGSAARGHFDPEDLNLTDEALRLKYLGPQQTELFMPICATYLLIFVVGAVGNGLTCLVILRHKA---MRTPTNYYLFSLAVSDLLVLLV-GLPLELYE-MWHNYPFLLGVGGCYFRTLLFEMVCLASVLNVTALSVERYVAVVHPLQARSMVTRAHVRRVLGAVWGLAMLCSLPNTSLHGIRQLH-VPCRGP------------------------------------------VPDSAVCMLVRPRALYNM-VVQTTALLFFCLPMAIMSVLYLLIGLRLRRERLLLMQEAKGRGSAAARSRYTCRLQQ-------------------------HDRGRRQVTKMLFVLVVVFGICWAPFHADRVMWSVVSQ--WTDGLHLAFQHVHVISGIFFYLGSAANPVLYSLMSSRFRETFQEALCLGACCHRLRPRHSSHSLSRMTTGSTLCDVGSLGSWVHPLAGNDGPEAQQETDPS---------------------------------------

NMU2R ---------------MSGMEKLQNASWIYQQKLEDPFQKHLNSTEEYLAFLCGPRRSHF---FLPVSVVYVPIFVVGVIGNVLVCLVILQHQA---MKTPTNYYLFSLAVSDLLVLLL-GMPLEVYE-MWRNYPFLFGPVGCYFKTALFETVCFASILSITTVSVERYVAILHPFRAKLQSTRRRALRILGIVWGFSVLFSLPNTSIHGIKFHY-FPNGSL------------------------------------------VPGSATCTVIKPMWIYNF-IIQVTSFLFYLLPMTVISVLYYLMALRLKKDKSLE-----------------ADEGN----------------------ANIQRPCRKSVNKMLFVLVLVFAICWAPFHIDRLFFSFVEE--WSESLAAVFNLVHVVSGVFFYLSSAVNPIIYNLLSRRFQAAFQNVISSFHKQWHSQHDPQLPPAQRNIFLTECHFVELTEDIGPQFPCQSSMHNSHLPTALSSEQMSRTNYQSFHFNKT---------------------

NTR1_HUMA MRLNSSAPGTPGTPAADPFQRAQAGLEEALLAPGFGNASGNASERVLAAPSSELDVNTDIYSKVLVTAVYLALFVVGTVGNTVTAFTLARKKSLQSLQSTVHYHLGSLALSDLLTLLL-AMPVELYNFIWVHHPWAFGDAGCRGYYFLRDACTYATALNVASLSVERYLAICHPFKAKTLMSRSRTKKFISAIWLASALLTVPMLFTMGEQNR--SADGQ-------------------------------------------HAGGLVCTPTIHTATVKV-VIQVNTFMSFIFPMVVISVLNTIIANKLTVMVRQA-----------------AEQGQ-------VCTVGGEHSTFSMAIEPGRVQALRHGVRVLRAVVIAFVVCWLPYHVRRLMFCYISDEQWTPFLYDFYHYFYMVTNALFYVSSTINPILYNLVSANFRHIFLATLACLCPVWRRRRKRPAFSRKADSVSSNHTLSSNATRETLY-------------------------------------------------------

NTR2_HUMA -------------------------------METSSPRPPRPSSNPGLSLDARLGVDTRLWAKVLFTALYALIWALGAAGNALSVHVVLKARAGRA--GRLRHHVLSLALAGLLLLLV-GVPVELYSFVWFHYPWVFGDLGCRGYYFVHELCAYATVLSVAGLSAERCLAVCQPLRARSLLTPRRTRWLVALSWAASLGLALPMAVIMGQKHELETADGEP------------------------------------------EPASRVCTVLVSRTALQV-FIQVNVLVSFVLPLALTAFLNGVTVSHLLALCSQVPSTSTPGSSTPSRLELLSEEGLLSFIVWKKTFIQGGQVSLVRHKDVRRIRSLQRSVQVLRAIVVMYVICWLPYHARRLMYCYVPDDAWTDPLYNFYHYFYMVTNTLFYVSSAVTPLLYNAVSSSFRKLFLEAVSSLCGEHHPMKRLPPKPQSPTLMDTASGFGDPPETRT---------------------------------------------------------

TRFR_HUMA --------------------------------------MENETVSELNQTQLQPRAVVALEYQVVTILLVLIICGLGIVGNIMVVLVVMRTKH---MRTPTNCYLVSLAVADLMVLVAAGLPNITDS-IYGS--WVYGYVGCLCITYLQYLGINASSCSITAFTIERYIAICHPIKAQFLCTFSRAKKIIIFVWAFTSLYCMLWFFLLDLNIS----TYKDAIV-------------------------------------------ISCGYKISRNYYSP-IYLMDFGVFYVVPMILATVLYGFIARILFLNPIPSDPKENSKTWKNDSTHQNTNLN----------------VNTSNRCFNSTVSSRKQVTKMLAVVVILFALLWMPYRTLVVVNSFLSSP-------FQENWFLLFCRICIYLNSAINPVIYNLMSQKFRAAFRKLCNCKQKPTEKPANYSVALNYSVIKESDHFSTELDDITVTDTYLSATKVSFDDTCLASEVSFSQS-------------------------------

Group A8a

C3AR_HUMA --------------------MASFSAETNSTDLLSQPWNEPPVILSMVILSLTFLLGLPGNGLVLWVAGLKMQRTVNTIWFLHLTLADLLCCLSLPFSLAHLALQGQWPYGRFLCKLIPSIIVLNMFASVFLLTAISLDRCLVVFKPIWCQNHRNVGMACSICGCIWVVAFVMCIPVFVYREIF--TTDNHNRCGYKFGLSSSLDYPDFYGDPLENRSLENIVQRPGEMNDRLDPSSFQTNDHPWTVPTVFQPQTFQRPSADSLPRGSARLTSQNLYSNVFKPADVVSPKIPSGFPIEDHETSPLDNSDAFLSTHLKLFPSASSNSFYESELPQGFQDYYNLGQFTDDDQVPTPLVAITITRLVVGFLLPSVIMIACYSFIVFRMQRGRFAKSQSKTFRVAVVVVAVFLVCWTPYHIFGVLSLL---TDPETPLGKTLMSWDHVCIALASANSCFNPFLYALLGKDFRKKARQSIQGILEAAFSE--ELTRSTHCPSNNVISERNSTTV--------------------------------------------

C5AR_HUMA ------MNSFNYTTPDYGHYDDKDTLDLNTPVDKTSNTLRVPDILALVIFAVVFLVGVLGNALVVWVTAFEAKRTINAIWFLNLAVADFLSCLALPILFTSIVQHHHWPFGGAACSILPSLILLNMYASILLLATISADRFLLVFKPIWCQNFRGAGLAWIACAVAWGLALLLTIPSFLYRVVREEYFPPKVLCGVDYSHD------------------------------------------------------------------------------------------------------------------------------------------------------KRRERAVAIVRLVLGFLWPLLTLTICYTFILLRTWSRRATRS-TKTLKVVVAVVASFFIFWLPYQVTGIMMSF---LEPSSPTFLLLNKLDSLCVSFAYINCCINPIIYVVAGQGFQGRLRKSLPSLLRNVLTE--ESVVRESKSFTRSTVDTMAQKTQAV-----------------------------------------

C5L2_HUMA --------MGNDSVSYEYGDYSDLSDRPVDCLDGACLAIDPLRVAPLPLYAAIFLVGVPGNAMVAWVAGKVARRRVGATWLLHLAVADLLCCLSLPILAVPIARGGHWPYGAVGCRALPSIILLTMYASVLLLAALSADLCFLALGPAWWSTVQRACGVQVACGAAWTLALLLTVPSAIYRRLHQEHFPARLQCVVDYGGSS------------------------------------------------------------------------------------------------------------------------------------------------------STENAVTAIRFLFGFLGPLVAVASCHSALLCWAARR------CRPLG--TAIVVGFFVCWAPYHLLGLVLTV---AAPNSALLARALRAEPLIVGLALAHSCLNPMLFLYFGR---AQLRRSLPAACHWALRESQGQDESVDSKKSTSHDLVSEMEV--------------------------------------------

CML1_HUMA ---MRMEDEDYNTSISYGDEYPDYLDSIVVLEDLSPLEARVTRIFLVVVYSIVCFLGILGNGLVIIIATFKMKKTVNMVWFLNLAVADFLFNVFLPIHITYAAMDYHWVFGTAMCKISNFLLIHNMFTSVFLLTIISSDRCISVLLPVWSQNHRSVRLAYMACMVIWVLAFFLSSPSLVFRDTA--NLHGKISCFNNFSLS------------------------TPGSSSWPTHSQM-------------------------------------------------------------------------------------------------------------DPVGYSRHMVVTVTRFLCGFLVPVLIITACYLTIVCKLQRNRLAKT-KKPFKIIVTIIITFFLCWCPYHTLNLLEL---HHTAMPG--SVFSLGLPLATALAIANSCMNPILYVFMGQDFK-KFKVALFSRLVNALSE--DTGHSSYPSHRSFTKMSSMNERTSMNERETGML---------------------------------

FML1_HUMA -----------------METNFSTPLNEYEEVSYESAGYTVLRILPLVVLGVTFVLGVLGNGLVIWVAGFRMTRTVTTICYLNLALADFSFTATLPFLIVSMAMGEKWPFGWFLCKLIHIVVDINLFGSVFLIGFIALDRCICVLHPVWAQNHRTVSLAMKVIVGPWILALVLTLPVFLFLTTV-TIPNGDTYCTFNFASW-----------------------GGT------PEERL--------------------------------------------------------------------------------------------------------------KVAITMLTARGIIRFVIGFSLPMSIVAICYGLIAAKIHKKGMIKS-SRPLRVLTAVVASFFICWFPFQLVALLGTVWLKEMLFYGKYKIIDILVNPTSSLAFFNSCLNPMLYVFVGQDFRERLIHSLPTSLERALSE--DSA-PTNDTAANSASPPAETELQAM-----------------------------------------

FML2_HUMA -----------------METNFSIPLNETEEVLPEPAGHTVLWIFSLLVHGVTFVFGVLGNGLVIWVAGFRMTRTVNTICYLNLALADFSFSAILPFRMVSVAMREKWPFASFLCKLVHVMIDINLFVSVYLITIIALDRCICVLHPAWAQNHRTMSLAKRVMTGLWIFTIVLTLPNFIFWTTI-STTNGDTYCIFNFAFWGD-----------------------------TAVERL--------------------------------------------------------------------------------------------------------------NVFITMAKVFLILHFIIGFTVPMSIITVCYGIIAAKIHRNHMIKS-SRPLRVFAAVVASFFICWFPYELIGILMAVWLKEMLLNGKYKIILVLINPTSSLAFFNSCLNPILYVFMGRNFQERLIRSLPTSLERALTEVPDSA-QTSNTHTTSASPPEETELQAM-----------------------------------------

FMLR_HUMA -----------------METNSSLPTNISGGTPAVSAGYLFLDIITYLVFAVTFVLGVLGNGLVIWVAGFRMTHTVTTISYLNLAVADFCFTSTLPFFMVRKAMGGHWPFGWFLCKFLFTIVDINLFGSVFLIALIALDRCVCVLHPVWTQNHRTVSLAKKVIIGPWVMALLLTLPVIIRVTTV-PGKTGTVACTFNFSPW-----------------------TND------PKERI--------------------------------------------------------------------------------------------------------------NVAVAMLTVRGIIRFIIGFSAPMSIVAVSYGLIATKIHKQGLIKS-SRPLRVLSFVAAAFFLCWSPYQVVALIATVRIR-ELLQGMYKEIGIAVDVTSALAFFNSCLNPMLYVFMGQDFRERLIHALPASLERALTE--DST-QTSDTATNSTLPSAEVALQAK-----------------------------------------

GPR1_HUMA -----MEDLEETLFEEFENYSYDLDYYSLESDLEEKVQLGVVHWVSLVLYCLAFVLGIPGNAIVIWFTGLKWKKTVTTLWFLNLAIADFIFLLFLPLYISYVAMNFHWPFGIWLCKANSFTAQLNMFASVFFLTVISLDHYIHLIHPVLSHRHRTLKNSLIVIIFIWLLASLIGGPALYFRDTV-E-FNNHTLCYNNFQKH-------------------------------------------------------------------------------------------------------------------------------------------------DPDLTLIRHHVLTWVKFIIGYLFPLLTMSICYLCLIFKVKKRTVLIS-SRHFWTILVVVVAFVVCWTPYHLFSIWELT---IHHNSYSHHVMQAGIPLSTGLAFLNSCLNPILYVLISKKFQARFRSSVAEILKYTLWE--VSCSGTVSEQLRNSETKNLCLLETAQ----------------------------------------

GPR44 -----------MSANATLKPLCPILEQMSRLQSHSNTSIRYIDHAAVLLHGLASLLGLVENGVILFVVGCRMRQTVVTTWVLHLALSDLLASASLPFFTYFLAVGHSWELGTTFCKLHSSIFFLNMFASGFLLSAISLDRCLQVVRPVWAQNHRTVAAAHKVCLVLWALAVLNTVPYFVFRDTI-SRLDGRIMCYYNVLLLN------------------------------PGPDR--------------------------------------------------------------------------------------------------------------DATCNSRQAALAVSKFLLAFLVPLAIIASSHAAVSLRLQHRGRRRP-GRFVRLVAAVVAAFALCWGPYHVFSLLEAR---AHANPGLRPLVWRGLPFVTSLAFFNSVANPVLYVLTCPDMLRKLRRSLRTVLESVLVD--DSELGGAGSSRRRRTSSTARSASPLALCSRPEEPRGPARLLGWLLGSCAASPQTGPLNRALSSTSS

GPRW MNGVSEGTRGCSDRQPGVLTRDRSCSRKMNSSGCLSEEVGSLRPLTVVILSASIVVGVLGNGLVLWMTVFRMARTVSTVCFFHLALADFMLSLSLPIAMYYI-VSRQWLLGEWACKLYITFVFLSYFASNCLLVFISVDRCISVLYPVWALNHRTVQRASWLAFGVWLLAAALCSAHLKFRTT--RKWNGCTHCYLAFNSDN-----------------------E------TAQIWI--------------------------------------------------------------------------------------------------------------EGVVEGHIIGTIGHFLLGFLGPLAIIGTCAHLIRAKLLREGWVHA-NRPKRLLLVLVSAFFIFWSPFNVVLLVHL-WRRVMLKEIYHPRMLLILQASFALGCVNSSLNPFLYVFVGRDFQEKFFQSLTSALARAFGE--EEFLSSCPRGNAPRE---------------------------------------------------

Group A8b

3hMrgF ----------------------------------------------------------------------------YI---FLLLCLCGLVGNGL-VLWFFGFSIKRNPFSIYFLHLASADVGYLFSKAVFSILNTGGFLGTFADYIRSVC-RVLGLCMFL-TGVSLLPAVSAE-RCASVIFPAWYWRRRP-KRLSAVVCALLWVLSLLVTCLHNYFCVFLGRGA-PG-AACRHMDIFLGILLFLLCCP----LMVLPCLALILHVECRAR--RRQRSAKLNHVILAMVSVFLVSSIYLGIDWFLFWVFQIPAP----FPEYVTDLCICINSSAKPIVYFLAGRDKSQRLWEP--LRVVFQRALRDGAELGEAGG------------------------------

5hMAS1 ----------------------------------------------------STGRNA-----SVGNAHRQ---IPIVHWVIMSISPVGFVENGI-LLWFLCFRMRRNPFTVYITHLSIADISLLFCIFILSIDYALDYELSSGHYYTIVTLSVTFLFGYN-TGLYLLTAISVE-RCLSVLYPIWYRCHRP-KYQSALVCALLWALSCLVTTMEYVMCIDREEES-HSRNDCRAVIIFIAILSFLVFTP----LMLVSSTILVVKIRKNTW--ASHSS-KLYIVIMVTIIIFLIFAMPMRLLYLLYYEYWST----FGNLHHISLLFSTINSSANPFIYFFVGSSKKKRFKES--LKVVLTRAFKDEMQPRRQKDNCNTVTVE---TVV----------------

8hMrgD -----------------------------------------------------SALNY----SRGSTVHTAYLVLSSL---AMFTCLCGMAGNSM-VIWLLGFRMHRNPFCIYILNLAAADLLFLFSMASTLSLE-TQPLVNTTDKVHELM-KRLMYFAYT-VGLSLLTAISTQ-RCLSVLFPIWFKCHRP-RHLSAWVCGLLWTLCLLMNGLTSSFCSKFLKFNEDR---CFRVDMVQAALIMGVLTP----VMTLSSLTLFVWVRRSSQQWRRQPT-RLFVVVLASVLVFLICSLPLSIYWFVLYWLSLPPEMQVL-CFSLSRLSSSVSSSANPVIYFLVGSR------------------------------------------------------------

60hDRR1 -----------------------------------------------------NGREE-------TPCYNQTLSFTGL---TCIISLVALTGNAV-VLWLLGCRMRRNAVSIYILNLVAANFLFLSGHIIFSPLPLIN----IRHPISKIL-SPVMTFPYF-IGLSMLSAISTE-RCLSILWPIWYHCRRP-RYLSSVMCVLLWALSLLRSILEWMFCDFLFSGA-NS-VWCETSDFITIA-WLVFLCV----VLCGSSLVLLVRILCGSR--KMPLT-RLYVTILLTVLVFLLCGLPFGIQWALFSRIHLDWKVLFCHVHLVSIFLSALNSSANPIIYFFVGSFRQRQNRQN--LKLVLQRALQDTPEVDEGGGWLPQETLELSGSKLEQ--------------

61hMrgX3 -----------------------------------------------------NGREE-------TPCYKQTLSFTGL---TCIVSLVALTGNAV-VLWLLGCRMRRNAVSIYILNLVAADFLFLSGHIICSPLRLIN----IRHPISKIL-SPVMTFPYF-IGLSMLSAISTE-RCLSILWPIWYHCRRP-RYLSSVMCVLLWALSLLRSILEWMFCDFLFSGA-DS-VWCETSDFITIA-WLVFLCV----VLCGSSLVLLVRILCGSR--KMPLT-RLYVTILLTVLVFLLCGLPFGIQWALFSRIHLDWKVLFCHVHLVSIFLSALNSSANPIIYFFVGSFRQRQNRQN--LKLVLQRALQDTPEVDEGGGWLPQETLELSGSRLEQ--------------

62hDRR2 -----------------------------------------------------NGREE-------TPCYKQTLSFTGL---TCIVSLVALTGNAV-VLWLLGCRMRRNAVSIYILNLVAADFLFLSGHIICSPLRLIN----ISHPISKIL-SPVMTFPYF-IGLSMLNAISTE-RCLSILWPIWYHCRRP-RYLSSVMCVLLWAPSLLRSILEWMFCDFLFSGA-DS-VRCETSDFITIA-WLVFLRV----VLCGSSLVLLVRILCGSR--KMPLT-RLYVTILLTVLVFLLCGLPFGIQWALFSRIHLDWKVLFCHVHLVSIFLSALNSSANPIIYFFMGSFRQLQNRKT--LKLVLQRDLQDTPEVDEGGWWLPQETLELSGSKLEI--------------

63hMRGpse -----------------------------------------------------NGREE-------TPCYKQTLSFTGL---TCIVSLVALTGNAV-VLWLLGCRMRRNAVSIYILNLVAADFLFLSGHIICSPLRLIN----IRHPISKIL-SPVMTFPYF-IGLSMLSAISTE-RCLSILWPIWYHCRRP-RYLSSVMCVLLWCLSLLRRIMEWMICDYLLSGAANA-GWCKTAD-ITTA-WLVVLGV----LRGGCSVVLLMRMR-GSR--KMPLR-MFELIILLMVVVLLRGVLPFGIQWALFSRIHLEWKVLFCHVHLVSIFLSALNSSANPIIYFFVGSFRQRQNRQN--LKLVLQRALQDTPEVDEGGGWLPQETLELSGSRL----------------

64hMRGpse -----------------------------------------------------NGTEE-------TPCYNQTLSFTVL---TCIVSLVALTGNAV-VLWLLGFRMCRNAVSIYILNLVAANFLLLSSHIIHSLLHLIN----NVHPISLIL--PVMTFPYL-AGLNILSAMSTK-RCLSILWPIW-RCRHP-THLSTVVCVLLWALSLL-SILEWMFCDSLFSDA-DS-VWCQTFRFITVT-WLIFLFV----VLCVSSLVLVVRILCGSQ--KMPLT-RLYMTILLTVLVFLLCGLPIGIQWALFSRIHMDWEVLYSHVHLPSIFLSSLNSSANPIIYFFMGFVRQHQNWQN--LKLVLQRDLQDTPEVD----------------------------------

65hDRR6 -----------------------------------------------------NGREE-------TPCYNQTLSFTVL---TCIISLVGLTGNAV-VLWLLGYRMRRNAVSIYILNLAAADFLFLSFQIIRSPLRLIN----ISHLIRKIL-VSVMTFPYF-TGLSMLSAISTE-RCLSVLWPIWYRCRRP-THLSAVVCVLLWGLSLLFSMLEWRFCDFLFSGA-DS-SWCETSDFIPVV-WLIFLCV----VLCVSSLVLLVRILCGSR--KMPLT-RLYVTILLTVLVFLLCGLPFGILGALIYRMHLNLEVLYCHVYLVCMSLSSLNSSANPIIYFFVGSFRQRQNRQN--LKLVLQRALQDKPEVDKGEGQLPEESLELSGSKLGP--------------

66hMrgX4 -----------------------------------------------------NGREE-------TPCYNQTLSFTVL---TCIISLVGLTGNAV-VLWLLGYRMRRNAVSIYILNLAAADFLFLSFQIIRSPLRLIN----ISHLIRKIL-VSVMTFPYF-TGLSMLSAISTE-RCLSVLWPIWYRCRRP-THLSAVVCVLLWGLSLLFSMLEWRFCDFLFSGA-DS-SWCETSDFIPVA-WLIFLCV----VLCVSSLVLLVRILCGSR--KMPLT-RLYVTILLTVLVFLLCGLPFGILGALIYRMHLNLEVLYCHVYLVCMSLSSLNSSANPIIYFFVGSFRQRQNRQN--LKLVLQRALQDKPEVDKGEGQLPEESLELSGSRLGP--------------

67hDRR5 -----------------------------------------------------NGREE-------TPCYKQTLSFTVL---TCIISLVGLTGNAV-VLWLLGCRMRRNAVSIYILNLAAADFLFLSFQIICRPLRLIN----ISHLIRKIL-VSVMTFPYF-TGLSMLSAISTE-RCLSVLWPIWYRCRRP-THLSAVVCVLLWAGLLLFSMLEWRFCDFLFSGA-DS-SWCETSDFIPVA-WLIFLCV----VLCVSSLVLLVRILCGSR--KMPLT-RLYVTILLTVLVFLLCGLPFGILGALIYRMHLNLEVLYCHVYLVCMSLSSLNSSANPIIYFFVGSFRQRQNRQN--LKLVLQRALQDKPEVDKGEGQLPEESLELSGRRLGP--------------

68hDRR4 -----------------------------------------------------NGTEE-------TLCYKQTLSLTVL---TCIVSLVGLTGNAV-VLWLLGCRMRRNAFSIYILNLAAADFLFLSGRLIYSLLSFIS----IPHTISKIL-YPVMMFSYF-AGLSFLSAVSTE-RCLSVLWPIWYRCHRP-THLSAVVCVLLWALSLLRSILEWMLCGFLFSGA-DS-AWCQTSDFITVA-WLIFLCV----VLCGSSLVLLIRILCGSR--KIPLT-RLYVTILLTVLVFLLCGLPFGIQFFLFLWIHVDREVLFCHVHLVSIFLSALNSSANPIIYFFVGSFRQRQNRQN--LKLVLQRALQDASEVDEGGGQLPQETLELSGSRLEQ--------------

69hMrgX1 -----------------------------------------------------NGTEE-------TLCYKQTLSLTVL---TCIVSLVGLTGNAV-VLWLLGCRMRRNAFSIYILNLAAADFLFLSGRLIYSLLSFIS----IPHTISKIL-YPVMMFSYF-AGLSFLSAVSTE-RCLSVLWPIWYRCHRP-THLSAVVCVLLWALSLLRSILEWMLCGFLFSGA-DS-AWCQTSDFITVA-WLIFLCV----VLCGSSLVLLIRILCGSR--KIPLT-RLYVTILLTVLVFLLCGLPFGIQFFLFLWIHVDREVLFCHVHLVSIFLSALNSSANPIIYFFVGSFRQRQNRQN--LKLVLQRALQDASEVDEGGGQLPEEILELSGSRLEQ--------------

70hDRR3 -----------------------------------------------------NGTEE-------TLCYKQTLSLTVL---TCIVSLVGLTGNAV-VLWLLGCRMRRNAFSIYILNLAAADFLFLSGRLIYSLLSFIS----IPHTISKIL-YPVMMFSYF-AGLNFLSAVSTD-RCLSVLWPIWYRCHRP-THLSAVVCVLLWALSLLRSILEWMLCGFLFSGA-DS-AWCQTSDFITVA-WLIFLCV----VLCGSSLVLLIRILCGSR--KIPLT-RLYVTILLTVLVFLLCGLPFGIQFFLFLWIHVDREVLFCHVHLVSIFLSALNSSANPIIYFFVGSLRQRQNRQN--LKLVLQRALQDTPEVDEGGGWLPQETLELSGSRLEQ--------------

71hMRGpse -----------------------------------------------------------------------------L---TCIVSLVGMTGNAV-VLWLLGFRMRRNAFSIYIFNLSMADFLFLRSHIIRFPLSLIN----ILHPIFKIL-SPVMMFSYL-ASLSFLSAMSTE-RCLYVLWPIW-RCRPR-PHLSAVVCVMLWALSLLRSILEWSFCDFLFSGA-DS-VWC-TSDFIIVG-GLIFLCV----ALCGSSLVLLVRILCGSR--KMPLT-RLYVTILLIALVFLLCGLPFGIRFFLFSWNHVDLEVLYCHVHLVSIFLSSLN--ANPNIYFFVGSFRQCQNRQN--LKLVLQRALQDTTEVD----------------------------------

72hMRGpse -----------------------------------------------------NRTEE-------TPCYKQTLSLMGL---TCIISLVTLTGNAV-VLWLLGFRMRRNAVSIYILNLAAADFLFLSGHVIRSASLLIN----ICHPISKIL-IPVMTFLYF-TGLSFLSAMSTE-RCLCVLWPIWYRCRPPPTHLSAVVCVLLWALSLLRSILE-MFCDFLFSDA-DS-IWCQPSDFITVV-WLIFLCV----VLCGSSLVLLIRILCGSW--KMPLT-GLYVTILLTVLVFLLRSLPFGIRWALSTGIHLDLEVIFCHVHLVSIFLSPLNGSANPVIYFFVGSFRQRQNRQN--LKLVLQRALQDMPEVKVEGG-FREGTLELSGS------------------

73hMRGpse -----------------------------------------------------SDTEE----THPHRCGMEVLVLIVL---ILIIDLVGLAGNAV-MLWLLGFCMHSNTFSLYILNLARADFLCTCFQIITFINFFSDFVSSLSIHFSRFV-TTVLFCACI-TGLSMLSTISTEHRLLPSLWPIWYCCHCP-THLSAVMCVLLWALSLLQSILEWMFCSFLFSDV-DSDNWCQILDFLTAV-WLIFLIL----VLCGFTLVLLVRIICGSQ--KMPLT-RLYVTILLTGLVFLFCSLPLSIQFLL-YWIEKDLDDLPCVVRLISIFLSALNSSANPIIYFFMGSFRQLQNRKT--LKLVLQRALQDMLEVDEGGGQLPEETLKLSGSRL----------------

74hMRGpse -----------------------------------------------------NRTEE----THLQHCGMEIMILMLL---LLIVDLVQLAGNGSHFLWLLGFRLHRNTFSLYTLNLAGADF-FLCSQILEIVNFYHD----FFLSISTYF-TTVMTFLYF-TGLSMLGSISTK-HCLSILWPI-YRCHHP-THLSAVVC-LLWALSLLQSILEWMFCGFLSSGA-DS-VWCETSDFITVT-WLIFLCV----VLCGSSPVLLVRILCGSR--KMPLT-RLYMTILLRVLVFLLCDLPFGIQF-LFFWIHVD-----CHVRLVSIFLSTLNSSANPIIHFFMGSFRQLQNRKT--L-LVLQRALQDTPEVEEGRWRLSEETLELS-SRL----------------

75hMrgX2 -----------------------------------------------------NGNDQ----ALLLLCGKETLIPVFL---ILFIALVGLVGNGF-VLWLLGFRMRRNAFSVYVLSLAGADFLFLCFQIINCLVYLSNFFCSISINFPSFF-TTVMTCAYL-AGLSMLSTVSTE-RCLSVLWPIWYRCRRP-RHLSAVVCVLLWALSLLLSILEGKFCGFLFSDG-DS-GWCQTFDFITAA-WLIFLFM----VLCGSSLALLVRILCGSR--GLPLT-RLYLTILLTVLVFLLCGLPFGIQWFLILWIWKDSDVLFCHIHPVSVVLSSLNSSANPIIYFFVGSFRKQWRLQQPILKLALQRALQDIAEVDHSEGCFRQGTPEMSRSSLV---------------

76hMRGpse ---------------------------------------------------------------------------------------------------------------LF----CTCD---LN--------------------TLNFF-TSVMTFAYL-VGLSMLSAISTE-CCLSVLRPIWYCCCCP-RNLSTVMCALPWALSLLLNTLEGKFCGFLVSNG-DY-GWCWTFDFITAV-WLVFIFLNFFCVLCESSLVLLVRILCGSL--HILLT-TL-LTILLTVLLFLICSLPLGIKWFLLFWILVDFDIFLCHLQPVSDVLSSLNSSANPIIYFFMGSFRQCQNWQN--LKLVLQKALQDTPEVDEGGGRLPQETLEMSGSRL----------------

77hMRGpse -----------------------------------------------------NGNEK-----NLSTCDLETLIPNLL---TCIIALDGLAGNAV-VLWLLGFHVPKNTFSIYMLNLARADYLFLSGHIIHSPM-FISTFSSISIYFPSFF-NAVMILSYL-AGLSMLSTISIK-HCISALWPIWYHCRRP-THLSAVLCALLWAPSLLLAFLEGYYCAFLFKIG-DY-SWFQTFDFITGT-WLIFKFV----VLCGSNLVLLVRILCGSQ--QMPLS-GL-MTILLTVLVFLLCSLPLGIQFLLF-WIK-NFHVFLCHVLPVSVVWSSLNSSANPIIYFFLGSFRQRQNRQT--LKLVLQRALQDMPRIDHREGYFAQGKLELSGSGL----------------

78hMRGpse ---------------------------------------------------------------------------------------------------------------------LARDFLFLCCQILDSLLKLISSFHPISISIPYFC-ITMITYFYI-TSLGMLSAISTK-HCMSVLWCIWYHCYHHLRQKSAVICGIL-VLSLLLSILERNYCSFFWGDS-NF-AW-----FLA----LYFITM----VLSGSSLSLLVRLLCGCR--SLQLT-RLLCDNLLLVLVFHFCSLPAGVQLFLGLWVQIDYNSFFC-LDCIVFILTSLAAAPTLLFAFFVGSFRY-QYGKI--LKLVLQRALQDTAEVYEGGGRLVQETLKLSGSRL----------------

80hMrgE -------------------------------------------------------------------------------------HMVAIVPD----L-LQG-------------RL---DF---------------------PGFVQTSL-ATLRFFCYI-VGLSLLAAVSVE-QCLAALFPAWYSCRRP-RHLTTCVCALTWALCLLLHLLLSSACTQFFGEP-SR-HLCRTLWLVAAV-LLALLCC----TMCGASLMLLLRVERGPQ--RPPPR-GFPGLILLTVLLFLFCGLPFGIYWLSRNLLWYIPH-YFYHF---SFLMAAVHCAAKPVVYFCLGSAQGR--RLP--LRLVLQRALGDEAELGAVRETSRRGLVDIAA-------------------

82hMrgG ----------------------------------------------------------------------------YL---TLIVGLGGPVGNGL-VLWNLGFRIKKGPFSIYLLHLAAADFLFLSCRVGFSVAQ-------AALGAQDTL-YFVLTFLWFAVGLWLLAAFSVE-RCLSDLFPACYQGCRP-RHASAVLCALVWTPTLPAVPLPANACGLLRNSA-CP-LVCPRYHVASVT-WFLVLAR----VAWTAGVVLFVRVTCCST--R-PRP-RLYGIVLGALLPALLCGLPSVFYWSLQPLLNF----LLPVFSPLATLLACVNSSSKPLIYSGLG--RQPGKREP--LRSVLRRALGEGAEL-----------------------------------

MRG_HUMA MVWGKICWFSQRAGWTVFAESQISLSCSLCLHSGDQEAQNPNLVSQLCGVFLQNETNETIHMQMSMAVGQQALPLNIIAPKAVLVSLCGVLLNGT-VFWLLCCGA-TNPYMVYILHLVAADVIYLCCSAVGFLQVTLLTYHGVVFFIPDFL-AILSPFSFE-VCLCLLVAISTE-RCVCVLFPIWYRCHRP-KYTSNVVCTLIWGLPFCINIVKSLFLTY---WK-HV-KACVIFLKLSGL-FHAILSL----VMCVSSLTLLIRFLCCSQ--QQKAT-RVYAVVQISAPMFLLWALPLSVAPLI-----TDFKMFVTTSYLISLFL-IINSSANPIIYFFVGSLRKKRLKES--LRVILQRALADKPEVGRNKKAAGIDPMEQPHSTQHVENLLPREHRVDVET

Group A9a

GP83_HUMA ---------------------------MVPHLLLLCLLPLVRATEPHEGRADEQSAEAALAVPNASHFFSWNNYTFSDWQNFVGRRRYGAESQNPTVKALLIVAYSFIIVFSLFGNVLVCHVIFKNQRMHSATSLFIVNLAVADIMITLLNTPFTLVRFVNSTWIFGKGMCHVSRFAQYCSLHVSALTLTAIAVDRHQVIMHPLKPRISITKGVIYIAVIWTMATFFSLPHAICQKLFTFKYSEDIVRSLCLPDFPE-PADLFWKYLDLATFILLYILPLLIISVAYARVAKKLWLCNMIGDVTTEQYF-ALRRKKKKTIKMLMLVVVLFALCWFPLNCYVLLLSSK----VIRTNNALYFAFHWFAMSSTCYNPFIYCWLNENFRIELKALLSMCQRPPKPQEDG---------------------QPSPVPSFRVAWTEKNDGQRAPLANNLLPTSQLQSGKTDLSSVEPIVTMS-------------

GP19_HUMA ---------------------------------MVFAHRMDNSKPHLIIPTLLVPLQNRSCTETATPLPSQYLMELSEEHSWMSNQTDLHYVLKPGEVATASIFFGILWLFSIFGNSLVCLVIHRSRRTQSTTNYFVVSMACADLLISVASTPFVLLQFTTGRWTLGSATCKVVRYFQYLTPGVQIYVLLSICIDRFYTIVYPLSFKVSREKAKKMIAASWIFDAGFVTPVLFFYGSNW-----D---SHCNYFLPSSWEGT---AYTVIHFLVGFVIPSVLIILFYQKVIKYIWRIGTDGR-TVRRTMNIVPRTKVKTIKMFLILNLLFLLSWLPFHVAQLWHPHEQD---YKKSSLVFTAITWISFSSSASKPTLYSIYNANFRRGMKETF--C-MSSMKCYRSNAYTITTSSRMAKKNYVGISEIPSMAKTITKDSIYDSFDREAKEKKLAWPINSNPPNTFV------------------------

NK1R_HUMA -------------------------------------------------------MDNVLPVDSDLSPNISTN-----------TSEPNQFVQPAWQIVLWAAAYTVIVVTSVVGNVVVMWIILAHKRMRTVTNYFLVNLAFAEASMAAFNTVVNFTYAVHNEWYYGLFYCKFHNFFPIAAVFASIYSMTAVAFDRYMAIIHPLQPRLSATATKVVICVIWVLALLLAFPQGYYSTTETMP-----SRVVCMIEWPEHPNKIYEKVYHICVTVLIYFLPLLVIGYAYTVVGITLWASEIPGD-SSDRYH-EQVSAKRKVVKMMIVVVCTFAICWLPFHIFFLLPYINPDLYLKKFIQQVYLAIMWLAMSSTMYNPIIYCCLNDRFRLGFKHAFRCCPFISAGDYEGLEMKSTRYLQT-QGSVYKVSRLET--TISTVVGAHEEEPEDGPKATP-SSLDLTSNCSSRSDSKTMTESFSFSSNVLS------

NK2R_HUMA -----------------------------------------------------------------MGTCDIVTEANISSGPESNTTGITAFSMPSWQLALWATAYLALVLVAVTGNAIVIWIILAHRRMRTVTNYFIVNLALADLCMAAFNAAFNFVYASHNIWYFGRAFCYFQNLFPITAMFVSIYSMTAIAADRYMAIVHPFQPRLSAPSTKAVIAGIWLVALALASPQCFYSTVTMDQ-----GATKCVVAWPEDSGGKTLLLYHLVVIALIYFLPLAVMFVAYSVIGLTLWRRAVPGH-QAHGANLRHLQAKKKFVKTMVLVVLTFAICWLPYHLYFILGSFQEDIYCHKFIQQVYLALFWLAMSSTMYNPIIYCCLNHRFRSGFRLAFRCCPWVTPTKEDKLELTPTTSLST------RVNRCHTKETLFMAGDTAPSEATSGEAGRPQDGSGLWFGYGLLAPTKTHVEI---------------

NK3R_HUMA MATLPAAETWIDGGGGVGADAVNLTASLAAGAATGAVETGWLQLLDQAGNLSSSPSALGLPVASPAPSQPWANLT-------------NQFVQPSWRIALWSLAYGVVVAVAVLGNLIVIWIILAHKRMRTVTNYFLVNLAFSDASMAAFNTLVNFIYALHSEWYFGANYCRFQNFFPITAVFASIYSMTAIAVDRYMAIIDPLKPRLSATATKIVIGSIWILAFLLAFPQCLYSKTKVMP-----GRTLCFVQWPEGPKQHF--TYHIIVIILVYCFPLLIMGITYTIVGITLWGGEIPGD-TCDKYH-EQLKAKRKVVKMMIIVVMTFAICWLPYHIYFILTAIYQQLNRWKYIQQVYLASFWLAMSSTMYNPIIYCCLNKRFRAGFKRAFRWCPFIKVSSYDELELKTTRFHPNRQSSMYTVTRMESMTVVFD--PNDADTTRSSRKKRA-TPRDPSFNGCSRRNSKSASATSSFISSPYTSVDEYS

NK4R_HUMA --------------------------------------MASPAGNLSAWPGWGWPPPAALRNLTSSPAPTASPSPAPSWTPSPRPGPAHPFLQPPWAVALWSLAYGAVVAVAVLGNLVVIWIVLAHKRMRTVTNSFLVNLAFADAAMAALNALVNFIYALHGEWYFGANYCRFQNFFPITAVFASIYSMTAIAVDRYMAIIDPLKPRLSATATRIVIGSIWILAFLLAFPQCLYSKIKVMP-----GRTLCYVQWPEGSRQHF--TYHMIVIVLVYCFPLLIMGITYTIVGITLWGGEIPGD-TCDKYQ-EQLKAKRKVVKMMIIVVVTFAICWLPYHIYFILTAIYQQLNRWKYIQQVYLASFWLAMSSTMYNPIIYCCLNKRFRAGFKRAFRWCPFIHVSSYDELELKATRLHPMRQSSLYTVTRMESMSVVFD--SNDGDSARSSHQKRG-TTRDVGSNVCSRRNSKSTSTTASFVSSSHMSVEEGS

Group A9b

PKR1_HUMA METTMGFMDDNATNTSTSFLSVLNPHGAHATSFPFNFSYSDYDMPLDEDEDVTNSRTFFAAKIVIGMALVGIMLVCGIGNFIFIAALVRYKKLRNLTNLLIANLAISDFLVAIVCCPFEMDYYVVRQLSWEHGHVLCTSVNYLRTVSLYVSTNALLAIAIDRYLAIVHPLRPRM--KCQTATGLIALVWTVSILIAIPSAYFTTET-------VLVIVKSQEKIFCGQIWPV-DQ-QLYYKSYFLFIFGIEFVGPVVTMTLCYARISREL----------------------------------------------WFK----------AVPG-------------------------------------------------FQTEQIRKRLRCRRKTVLVLMCILTAYVLCWAPFYGFTIVRDFFPTVFVKEKHYLTAFYIVECIAMSNSMINTLCFVTVKNDTVKYFKKIMLLHWKASYNGGKSSADLDLKTIGMPATEEVDCIRLK----------------------------------------------------------------------------------------------------------------------------------------------------------------------------------------------------------------------------------------------------------------------------

GP10_HUMA --MASSTTRGPRVSDLFSGLPPAVTTPANQSAEASAGNGSVAGADAPAVTPFQSLQLVHQLKGLIVLLYSVVVVVGLVGNCLLVLVIARVRRLHNVTNFLIGNLALSDVLMCTACVPLTLAYAFEPR-GWVFGGGLCHLVFFLQPVTVYVSVFTLTTIAVDRYVVLVHPLRRRI--SLRLSAYAVLAIWALSAVLALPAAVHTY----------HVELKPHDVRLCEEFWGSQER---QRQLYAWGLLLVTYLLPLLVILLSYVRVSVKL----------------------------------------------RNR----------VVPGCV--------------------------------------------TQSQA----DWDRARRRRTFCLLVVVVVVFAVCWLPLHVFNLLRDLDPHAI-DPYAFGLVQLLCHWLAMSSACYNPFIYAWLHDSFREELRKLLVAWPRKIAPHGQNMTVSVVI--------------------------------------------------------------------------------------------------------------------------------------------------------------------------------------------------------------------------------------------------------------------------------------------

L1A_HUMA -----------------------------------MQGNGSALPNASQPVLRGDGARPSWLASALACVLIFTIVVDILGNLLVILSVYRNKKLRNAGNIFVVSLAVADLVVAIYPYPLVLMSIFNNG--WNLGYLHCQVSGFLMGLSVIGSIFNITGIAINRYCYICHSLKYDKLYSSKNSLCYVLLIWLLTLAAVLPNL------------RAGTLQYDPRIYSCTFAQ-------SVSSAYTIAVVVFHFLVPMIIVIFCYLRIWILV----------------------------------------------LQV----------RQRV---------------------------------------------KPD-RKPKLK---PQDF-RNFVTMFVVFVLFAICWAPLNFIGLAVASDPASM-VPRIPEWLFVASYYMAYFNSCLNAIIYGLLNQNFRKEYRRIIVSLCTARVFFVDSSNDVADRVKWKPSPLMTNNNVVKVDSV------------------------------------------------------------------------------------------------------------------------------------------------------------------------------------------------------------------------------------------------------------------------

L1B_HUMA ----------------------MSENGSFANCCEAGGWAVRPGWSGAGSARPSRTPRPPWVAPALSAVLIVTTAVDVVGNLLVILSVLRNRKLRNAGNLFLVSLALADLVVAFYPYPLILVAIFYDG--WALGEEHCKASAFVMGLSVIGSVFNITAIAINRYCYICHSMAYHRIYRRWHTPLHICLIWLLTVVALLPNF------------FVGSLEYDPRIYSCTFIQ-------TASTQYTAAVVVIHFLLPIAVVSFCYLRIWVLV----------------------------------------------LQA----------RRKA----------------------------------------------KPESRLCLK---PSDL-RSFLTMFVVFVIFAICWAPLNCIGLAVAINPQEM-APQIPEGLFVTSYLLAYFNSCLNAIVYGLLNQNFRREYKRILLALWNPRHCIQDASKGSHAEGLQSPAPPIIGVQHQADAL-------------------------------------------------------------------------------------------------------------------------------------------------------------------------------------------------------------------------------------------------------------------------

L1X_HUMA ----------------------------------MGPTLAVPTPYGCIGCKLPQPEYPPALIIFMFCAMVITIVVDLIGNSMVILAVTKNKKLRNSGNIFVVSLSVADMLVAIYPYPLMLHAMSIGG--WDLSQLQCQMVGFITGLSVVGSIFNIVAIAINRYCYICHSLQYERIFSVRNTCIYLVITWIMTVLAVLPNM------------YIGTIEYDPRTYTCIFNY-------LNNPVFTVTIVCIHFVLPLLIVGFCYVRIWTKV----------------------------------------------LAA----------RDPA---------------------------------------------------GQNPDNQLAEV-RNFLTMFVIFLLFAVCWCPINVLTVLVAVSPKEM-AGKIPNWLYLAAYFIAYFNSCLNAVIYGLLNENFRREYWTIFHAMRHPIIFFPGLISDIREMQEARTLARARAHARDQAREQDRAHACPAVEETPMNVRNVPLPGDAAAGHPDRASGHPKPHSRSSSAYRKSASTHHKSVFSHSKAASGHLKPVSGHSKPASGHPKSATVYPKPASVHFKGDSVHFKGDSVHFKPDSVHFKPASSNPKPITGHHVSAGSHSKSAFSAATSHPKPIKPATSHAEPTTADYPKPATTSHPKPAAADNPELSASHCPEIPAIAHPVSDDSDLPESASSPAAGPTKPAASQLESDTIADLPDPTVVTTSTNDYHDVVVVDVEDDPDEMAV

NY1R_HUMA ----------------------MNSTLFSQVENHSVHSNFSEKNAQLLAFENDDCHLPLAMIFTLALAYGAVIILGVSGNLALIIIILKQKEMRNVTNILIVNLSFSDLLVAIMCLPFTFVYTLMDH--WVFGEAMCKLNPFVQCVSITVSIFSLVLIAVERHQLIINPRGWRP--NNRHAYVGIAVIWVLAVASSLPFLIYQVMT--DEPFQNVTLDAYKDKYVCFDQFPS-DS---HRLSYTTLLLVLQYFGPLCFIFICYFKIYIRL----------------------------------------------KRR--------------------------------------------------------NNMMDKMRDNKYR---SSETKRINIMLLSIVVAFAVCWLPLTIFNTVFDWNHQII-ATCNHNLLFLLCHLTAMISTCVNPIFYGFLNKNFQRDLQFFFNFCDFRSRDDDYETIAMSTMHTDVSKTSLKQASPVAFKKINNNDDNEKI---------------------------------------------------------------------------------------------------------------------------------------------------------------------------------------------------------------------------------------------------------------

NY2R_HUMA ------------MGPIGAEADENQTVEEMKVEQYGPQTTPRGELVPDPEPELIDSTKLIEVQVVLILAYCSIILLGVIGNSLVIHVVIKFKSMRTVTNFFIANLAVADLLVNTLCLPFTLTYTLMGE--WKMGPVLCHLVPYAQGLAVQVSTITLTVIALDRHRCIVYHLESKI--SKRISFLIIGLAWGISALLASPLAIFRE-------YSLIEIIPDFEIVACTEKWPG-EEKSIYGTVYSLSSLLILYVLPLGIISFSYTRIWSKL----------------------------------------------KNH----------VSPG-------------------------------------------------AANDHY---HQRRQKTTKMLVCVVVVFAVSWLPLHAFQLAVDIDSQVL-DLKEYKLIFTVFHIIAMCSTFANPLLYGWMNSNYRKAFLSAFRCEQRLDAIHSEVSVTFKAKKNLEVRKNSGPNDSFTEATNV-----------------------------------------------------------------------------------------------------------------------------------------------------------------------------------------------------------------------------------------------------------------------

NY4R_HUMA ---------------------MNTSHLLALLLPKSPQGENRSKPLGTPYNFSEHCQDSVDVMVFIVTSYSIETVVGVLGNLCLMCVTVRQKEKANVTNLLIANLAFSDFLMCLLCQPLTAVYTIMDY--WIFGETLCKMSAFIQCMSVTVSILSLVLVALERHQLIINPTGWKP--SISQAYLGIVLIWVIACVLSLPFLANSILENVFHKNHSKALEFLADKVVCTESWPL-AH---HRTIYTTFLLLFQYCLPLGFILVCYARIYRRL----------------------------------------------QRQ--------------------------------------------------------GRVFHK-GTYSLR---AGHMKQVNVVLVVMVVAFAVLWLPLHVFNSLEDWHHEAI-PICHGNLIFLVCHLLAMASTCVNPFIYGFLNTNFKKEIKALVLTCQQSAPLEESEHLPLSTVHTEVSKGSLRLSGRSNPI--------------------------------------------------------------------------------------------------------------------------------------------------------------------------------------------------------------------------------------------------------------------------

NY5R_HUMA -----------MSFYSKQDYNMDLELDEYYNKTLATENNTAATRNSDFPVWDDYKSSVDDLQYFLIGLYTFVSLLGFMGNLLILMALMKKRNQKTTVNFLIGNLAFSDILVVLFCSPFTLTSVLLDQ--WMFGKVMCHIMPFLQCVSVLVSTLILISIAIVRYHMIKHPISNNL--TANHGYFLIATVWTLGFAICSPLPVFHSLVELQETFGSALL---SSRYLCVESWPS-DS---YRIAFTISLLLVQYILPLVCLTVSHTSVCRSISCGLSNKENRLEENEMINLTLHPSKKSGPQVKLSGSHKWSYSFIKKHRRRYSKKTACVLPAPERPSQENHSRILPENFGSVRSQLSSSSKFIPGVPTCFEIKPEENSDVHELRVKRSVTRIKKRSRSVFYRLTILILVFAVSWMPLHLFHVVTDFNDNLI-SNRHFKLVYCICHLLGMMSCCLNPILYGFLNNGIKADLVSLIHCLHM-----------------------------------------------------------------------------------------------------------------------------------------------------------------------------------------------------------------------------------------------------------------------------------------------------------

Group A10

FSHR_HUMA --------------------MALLLVSLLAFLSLGSGCHHRICHCSNRVFLCQESKVTEIPSDLPRN------------------------AIELRFVLTKLRVIQKGAFSGFGDLEKIEISQNDVLEVIEADVFSNLPKLHEIRIEKANNLLYINPEAFQNLPNLQYLLISNTGIKHLPDVHKIHSLQKV-LLDIQDNINIHTIERNSFVGLSFESVILWLNKNGIQEIHNCAFNGT------------------------QLDELNLSDN-----------------------NNLEELPNDVFHGA-----------------------------------------------SGPVI----------------------------------------------------------------------LDISRTRIHSLPSYGLENLKKLRARSTYNLKKLPTLEKLVALMEASLTYPSHCCAFAN---------------------------------------------WRRQISELHPICNKSILRQEVDYMTQTRGQRSSLAEDNESSYSRGFDMTYTEFDYDLCNEVVDVTCSPKPDAFNPCEDIMGYNILRVLIWFISILAITGNIIVLVILTTSQYKLTVPRFLMCNLAFADLCIGIYLLLIASVDIHTKSQYHNYAIDWQTGAGCDAAGFFTVFASELSVYTLTAITLERWHTITHAMQLDCKVQLRHAASVMVMGWIFAFAAALFPIFGISSYMKVSICLPMDIDSPLSQLYVMSLLVLNVLAFVVICGCYIHIYLTVRNPNIVSSSSDTRIAKRMAMLIFTDFLCMAPISFFAISASLKVPLITVSKAKILLVLFHPINSCANPFLYAIFTKNFRRDFFILLSKCGCYEMQAQIYRTETSSTVHNTHPRNGHCSSAPRVTNGSTYILVPLSHLAQN---------------------------------------------------------------------------------

GPR48 -----MPGPLGLLCFLALGLLGSAGPSGAAPPLCAAPCS----CDGDRRVDCSGKGLTAVPEGLSAFTQALDISMNNITQLPEDAFKNFPFLEELQLAGNDLSFIHPKALSGLKEL-KVLTLQNNQLKTVPSEAIRGLSALQSLRLD-ANHITSVPEDSFEGLVQLRHLWLDDNSLTEVPV-HPLSNLPTLQALTLALN-KISSIPDFAFTNLSSL-VVLHLHNNKIRGLSQHCFDGLDNLETLDLSYNNLGEFPQAIKARPSLKELGFHSNSISVIPDGAFDGNPLLRTIHLYDNPLSFVGNSAFHNLSDLHSLVIRGASMVQQFPNLTGTVHLESLTLTGTKISSIPNNLCQEQKMLRTLDLSYNNIRDLPSFNGCHALEEISLQRNQIYQIKEGTFQGLISLRILDLSRNLIHEIHSRAFATLGPITNLDVSFNELTSFPTEGLNGLNQLKLVGNFKLKEALAAKDFVNLRSLSVPYAYQCCAFWGCDSYAN--------------------------------------------------LNTEDNSLQDHSVAQEKGTADAANVTSTLENEEHSQII--------------IHCTPSTGAFKPCEYLLGSWMIRLTVWFIFLVALFFNLLVILTTFASCTSLPSSKLFIGLISVSNLFMGIYTGILTFLDAVSWGRFAEFGIWWETGSGCKVAGFLAVFSSESAIFLLMLATVERSLSAKDIMKNGKSNHLKQFRVAALLAFLGATVAGCFPLFHRGEYSASPLCLPFPTGETPSLGFTVTLVLLNSLAFLLMAVIYTKLYCNLEKEDL-SENSQSSMIKHVAWLIFTNCIFFCPVAFFSFAPLITAISISPEIMKSVTLIFFPLPACLNPVLYVFFNPKFKEDWKLLKRRVTKKSGSVSVSISSQGGCLEQDFYYDCGMYSHLQGNLTVCDCCESFLLTKPVSCKHLIKSHSCPALAVASCQRPEGYWSDCGTQSAHSDYADEEDSFVSDSSDQVQACGRACFYQSRGFPLVRYAYNLPRVKD

GPR49 MDTSRLGVLLSLPVLLQLATGGSSPRSGVLLRGCPTHCHCEPDGRMLLRVDCSDLGLSELPSNLSVFTSYLDLSMNNISQLLPNPLPSLRFLEELRLAGNALTYIPKGAFTGLYSL-KVLMLQNNQLRHVPTEALQNLRSLQSLRLD-ANHISYVPPSCFSGLHSLRHLWLDDNALTEIPV-QAFRSLSALQAMTLALN-KIHHIPDYAFGNLSSL-VVLHLHNNRIHSLGKKCFDGLHSLETLDLNYNNLDEFPTAIRTLSNLKELGFHSNNIRSIPEKAFVGNPSLITIHFYDNPIQFVGRSAFQHLPELRTLTLNGASQITEFPDLTGTANLESLTLTGAQISSLPQTVCNQLPNLQVLDLSYNLLEDLPSFSVCQKLQKIDLRHNEIYEIKVDTFQQLLSLRSLNLAWNKIAIIHPNAFSTLPSLIKLDLSSNLLSSFPITGLHGLTHLKLTGNHALQSLISSENFPELKVIEMPYAYQCCAFGVCENAYK----------------------------------------------ISNQWNKGDNSSMDDLHKKDAGMFQAQDERDLEDFLLDFE--------EDLKALHSVQCSPSPGPFKPCEHLLDGWLIRIGVWTIAVLALTCNALVTSTVFRSPLYISPIKLLIGVIAAVNMLTGVSSAVLAGVDAFTFGSFARHGAWWENGVGCHVIGFLSIFASESSVFLLTLAALERGFSVKYSAKFETKAPFSSLKVIILLCALLALTMAAVPLLGGSKYGASPLCLPLPFGEPSTMGYMVALILLNSLCFLMMTIAYTKLYCNLDKGDL-ENIWDCSMVKHIALLLFTNCILNCPVAFLSFSSLINLTFISPEVIKFILLVVVPLPACLNPLLYILFNPHFKEDLVSLRKQTYVWTRS----KHPSLMSINSDDVEKQSCDSTQALVTFTSSSITYDLPPSSVPSPAYPVTESCHLSSVAFVPCL-----------------------------------------------------------

LSHR_HUMA ----------------MKQRFSALQLLKLLLLLQPPLPRALREALCPEPCNCVPDGALRCPGP-TAG------------------------LTRLSLAYLPVKVIPSQAFRGLNEVIKIEISQIDSLERIEANAFDNLLNLSEILIQNTKNLRYIEPGAFINLPRLKYLSICNTGIRKFPDVTKVFSSESNFILEICDNLHITTIPGNAFQGMNNESVTLKLYGNGFEEVQSHAFNGT------------------------TLTSLELKEN-----------------------VHLEKMHNGAFRGA-----------------------------------------------TGPKT----------------------------------------------------------------------LDISSTKLQALPSYGLESIQRLIATSSYSLKKLPSRETFVNLLEATLTYPSHCCAFRNLPTKEQ---------------------------------------------------NFSHSISENFSKQCESTVRKVSNKTLYSSMLAESELSGWDYEYGFCLP-KTPRCAPEPDAFNPCEDIMGYDFLRVLIWLINILAIMGNMTVLFVLLTSRYKLTVPRFLMCNLSFADFCMGLYLLLIASVDSQTKGQYYNHAIDWQTGSGCSTAGFFTVFASELSVYTLTVITLERWHTITYAIHLDQKLRLRHAILIMLGGWLFSSLIAMLPLVGVSNYMKVSICFPMDVETTLSQVYILTILILNVVAFFIICACYIKIYFAVRNPELMATNKDTKIAKKMAILIFTDFTCMAPISFFAISAAFKVPLITVTNSKVLLVLFYPINSCANPFLYAIFTKTFQRDFFLLLSKFGCCKRRAELYRRKDFSAYTSNCKNGFTGSNKPSQSTLKLSTLHCQGTALLDKTRYTEC--------------------------------------------------------------------------

TSHR_HUMA -------------MRPADLLQLVLLLDLPRDLGGMGCSSPPCECHQEEDFRVTCKDIQRIP-SLPPS------------------------TQTLKLIETHLRTIPSHAFSNLPNISRIYVSIDVTLQQLESHSFYNLSKVTHIEIRNTRNLTYIDPDALKELPLLKFLGIFNTGLKMFPDLTKVYSTDIFFILEITDNPYMTSIPVNAFQGLCNETLTLKLYNNGFTSVQGYAFNGT------------------------KLDAVYLNKN-----------------------KYLTVIDKDAFGGV----------------------------------------------YSGPSL----------------------------------------------------------------------LDVSQTSVTALPSKGLEHLKELIARNTWTLKKLPLSLSFLHLTRADLSYPSHCCAFKN-QKKIRGILESLMCNESSMQSLRQRKSVNALNSPLHQEYEENLGDSIVGYKEKSKFQDTHNNAHYYVFFEEQEDEIIGFGQELKNPQEETLQAFDSHYDYTICGDSEDMVCTPKSDEFNPCEDIMGYKFLRIVVWFVSLLALLGNVFVLLILLTSHYKLNVPRFLMCNLAFADFCMGMYLLLIASVDLYTHSEYYNHAIDWQTGPGCNTAGFFTVFASELSVYTLTVITLERWYAITFAMRLDRKIRLRHACAIMVGGWVCCFLLALLPLVGISSYAKVSICLPMDTETPLALAYIVFVLTLNIVAFVIVCCCHVKIYITVRNPQYNPGDKDTKIAKRMAVLIFTDFICMAPISFYALSAILNKPLITVSNSKILLVLFYPLNSCANPFLYAIFTKAFQRDVFILLSKFGICKRQAQAYRGQRVPPKNSTDIQVQKVTHDMRQGLHNMEDVYELIENSHLTPKKQGQISEEYMQTVL----------------------------------------------------------------

Group A11

GPR82 -------------------------------------MPFPNCSAPSTVVATAVGVLLGLECGLGLLGNAVALWTFLFR-VRVWK--PYAVYLLNLALADLLLAACLPFLAAFYLSLQAWHLGRVGCWALRFL----LDLSRSVGMAFLAAVALDRYLRVVHPRLK---------------V-NLLSPQAALGVSGLVWLLMVALTCPGL-----LISEAA----QNS-TRCHS----------FYSRADGSFSIIWQEALSCLQFV-LPFGLIVFCNAGIIRALQKRLREPEKQ---PKLQRAQALVTLVVVLFALCFLPCFLARVLMHIFQNL------GSCRALCAVAH-----------TSDVTGSLTYLHSVVNPVVYCFSSPTFRSSYRRVFHTLRGKGQAAEPPDFNPRDSYS--------------------------------------------------------

P2UR ------------------------------------MNNNTTCIQPSMISSMALPIIYILLCIVGVFGNTLSQWIFLTK-IGKKT--STHIYLSHLVTANLLVCSAMPFMSIYFLKGFQWEYQSAQCRVVNFLGTLSMHASMFVSLLILSWIAISRYATLMQKDSSQETTSCYEKIFYGHLLKKFRQPNFARKLCIYIWGVVLGIIIPVT-----VYYSVIEATEGEE-SLCYN------------RQMELGAMISQIAGLIGTTFIGFSFLVVLTSYYSFVSHLRKIRTCTSIMEKDLTYSSVKRHLLVIQILLIVCFLPYSIFKPIF-YVLH-----QRDNCQQLNYLIE-----------TKNILTCLASARSSTDPIIFLLLDKTFKKTLYNLFTKSNSAHMQSYG------------------------------------------------------------------

P2Y11 ------------------MAADLGPWNDTINGTWDGDELGYRCRFNEDFKYVLLPVSYGVVCVLGLCLNAVALYIFLCR-LKTWN--ASTTYMFHLAVSDALYAASLPLLVYYYARGDHWPFSTVLCKLVRFL----FYTNLYCSILFLTCISVHRCLGVLRPLRS---------------L-RWGRARYARRVAGAVWVLVLACQAPVL-----YFVTTSA--RGGR-VTCHD----------TSAPELFSRFVAYSSVMLGLLFA-VPFAVILVCYVLMARRLLKPAYGTSGGL-PRAKRKSVRTIAVVLAVFALCFLPFHVTRTLY-YSFRS---LDL-SCHTLNAINM-----------AYKVTRPLASANSCLDPVLYFLAGQRLVRFARDAKPPTGPSPATPARRRLGLRRSDRTDMQRIEDVLGSSEDSRRTESTPAGSENTKDIRL----------------------

GPR41 -----------------------MAANVSGAKSCPANFLAAADDKLSGFQGDFLWPILVVEFLVAVASNGLALYRFSIRKQRPWH--PAVVFSVQLAVSDLLCALTLPPLAAYLYPPKHWRYGEAACRLERFL----FTCNLLGSVIFITCISLNRYLGIVHPFFA---------------R-SHLRPKHAWAVSAAGWVLAALLAMPTL-----SFSHLKRPQQGAG--NCSVARPEACIKCLGTADHGLAAYRAYSLVLAGLGCG-LPLLLTLAAYGALGRAVLRSPGMT-----VAEKLRVAALVASGVALYASSYVPYHIMRVLN-VDAR---RRWSTRCPSFADIAQATAALELGPYVGYQVMRGLMPLAFCVHPLLYMAAVPS----LGCCCRHCPGYRDSWNPEDAKSTGQALPLNATAAPKPSEPQSRELSQ------------------------------------

GPR42 -------------------------------------MDTGPDQSYFSGNHWFVFSVYLLTFLVGLPLNLLALVVFVGK-LQR-RPVAVDVLLLNLTASDLLLLLFLPFRMVEAANGMHWPLPFILCPLSGFI----FFTTIYLTALFLAAVSIERFLSVAHPLWY---------------K-TRPRLGQAGLVSVACWLLASAHCSVVYVIEFSGDISHS---QGTN-GTCYL----------EFRKDQLAILLPVRLEMAVVLFV-VPLIITSYCYSRLVWILGRG------GSH-RRQRRVAGLLAATLLNFLVCFGPYNVSHVVG-YIC---GESPAWRIYV----------------------TLLSTLNSCVDPFVYYFSSSGFQADFHELLRRLCGLWGQWQQESSMELKEQKGGEEQRADRPAERKTSEHSQGCGTGGQVACAES-----------------------

GPR43 -------------------------------------MDTGPDQSYFSGNHWFVFSVYLLTFLVGLPLNLLALVVFVGK-LRC-RPVAVDVLLLNLTASDLLLLLFLPFRMVEAANGMHWPLPFILCPLSGFI----FFTTIYLTALFLAAVSIERFLSVAHPLWY---------------K-TRPRLGQAGLVSVACWLLASAHCSVVYVIEFSGDISHS---QGTN-GTCYL----------EFWKDQLAILLPVRLEMAVVLFV-VPLIITSYCYSRLVWILGRG------GSH-RRQRRVAGLVAATLLNFLVCFGPYNVSHVVG-YIC---GESPVWRIYV----------------------TLLSTLNSCVDPFVYYFSSSGFQADFHELLRRLCGLWGQWQQESSMELKEQKGGEEQRADRPAERKTSEHSQGCGTGGQVACAEN-----------------------

HM74 --------------------------------------------MLPDWKSSLILMAYIIIFLTGLPANLLALRAFVGR-IRQPQPAPVHILLLSLTLADLLLLLLLPFKIIEAASNFRWYLPKVVCALTSFG----FYSSIYCSTWLLAGISIERYLGVAFPVQY---------------K-LSRRPLYGVIAALVAWVMSFGHCTIVIIVQYLNTTEQVR--SGNE-ITCYE----------NFTDNQLDVVLPVRLELCLVLFF-IPMAVTIFCYWRFVWIMLSQPLVGA-----QRRRRAVGLAVVTLLNFLVCFGPYNVSHLVG-YHQR---KSPWWR----------------------SIAVVFSSLNASLDPLLFYFSSSVVRRAFGRGLQVLRNQGSSLLGRRGKDTAEGTNEDRGVGQGEGMPSSDFTTE------------------------------------

P2Y4 ------------------------MNRHHLQDHFLEIDKKNCCVFRDDFIAKVLPPVLGLEFIFGLLGNGLALWIFCFH-LKSWK--SSRIFLFNLAVADFLLIICLPFVMDYYVRRSDWNFGDIPCRLVLFM----FAMNRQGSIIFLTVVAVDRYFRVVHPHHA---------------L-NKISNWTAAIISCLLWGITVGLTVHLL-----KKKLLIQ--NGPA-NVCIS----------FS----ICHTFRWHEAMFLLEFL-LPLGIILFCSARIIWSLRQRQM--DRH---AKIKRAITFIMVVAIVFVICFLPSVVVRI--RIFW-LLHTSGTQNCEVYRSVDL-----------AFFITLSFTYMNSMLDPVVYYFSSPSFPNFFSTLINRCLQRKMTGEPDNNRSTSVELTGDPNKTRGAPEALMANSGEPWSPSYLGPTSNNHSKKGHCHQEPASLEKQLGCCIE

P2Y6 ----------------MASTESSLLRSLGLSPGPGSSEVELDCWFDEDFKFILLPVSYAVVFVLGLGLNAPTLWLFIFR-LRPWD--ATATYMFHLALSDTLYVLSLPTLIYYYAAHNHWPFGTEICKFVRFL----FYWNLYCSVLFLTCISVHRYLGICHPLRA---------------L-RWGRPRLAGLLCLAVWLVVAGCLVPNL-----FFVTTSN--KGTT-VLCHD----------TTRPEEFDHYVHFSSAVMGLLFG-VPCLVTLVCYGLMARRLYQPLPGSAQS---SSRLRSLRTIAVVLTVFAVCFVPFHITRTIY-YLARL---LEA-DCRVLNIVNV-----------VYKVTRPLASANSCLDPVLYLLTGDKYRRQLRQLCGGGKPQPRTAASSLALVSLPEDSSCRWAATPQDSSCSTPRADRL----------------------------------

P2YR -------------------------MEWDNGTGQALGLPPTTCVYRENFKQLLLPPVYSAVLAAGLPLNICVITQICTS-RRALT--RTAVYTLNLALADLLYACSLPLLIYNYAQGDHWPFGDFACRLVRFL----FYANLHGSILFLTCISFQRYLGICHPLAP---------------WHKRGGRRAAWLVCVAVWLAVTTQCLPTA-----IFAATGI--QRNR-TVCYD----------LSPPALATHYMPYGMALTVIGFL-LPFAALLACYCLLACRLCRQDGPAEPVA-QERRGKAARMAVVVAAAFAISFLPFHITKTAY-LAVRS---TPGVPCTVLEAFAA-----------AYKGTRPFASANSVLDPILFYFTQKKFRRRPHELLQKLTAKWQRQGR------------------------------------------------------------------

GPR80 MTEVLWPAVPNGTDAAFLAGPGSSWGNSTVASTAAVSSSFKCALTKTGFQFYYLPAVYILVFIIGFLGNSVAIWMFVFH-MKPWS--GISVYMFNLALADFLYVLTLPALIFYYFNKTDWIFGDAMCKLQRFI----FHVNLYGSILFLTCISAHRYSGVVYPLKS---------------L-GRLKKKNAICISVLVWLIVVVAISPIL-----FYSGTGV--RKNKTITCYD----------TTSDEYLRSYFIYSMCTTVAMFC-VPLVLILGCYGLIVRALIYKDLDN-----SPLRRKSIYLVIIVLTVFAVSYIPFHVMKTMN-LRARLDFQTPA-MCAFNDRVYA-----------TYQVTRGLASLNSCVDPILYFLAGDTFRRRLSRATRKASRRSEANLQSKSEDMTLNILPEFKQNGDTSL--------------------------------------------

GPR91 ------------------MNEPLDYLANASDFPDYAAAFGNCTDENIPLKMHYLPVIYGIIFLVGFPGNAVVISTYIFK-MRPWK--SSTIIMLNLACTDLLYLTSLPFLIHYYASGENWIFGDFMCKFIRFS----FHFNLYSSILFLTCFSIFRYCVIIHPMSC---------------F-SIHKTRCAVVACAVVWIISLVAVIPMT-----FLITSTN--RTNR-SACLD----------LTSSDELNTIKWYNLILTATTFC-LPLVIVTLCYTTIIHTLTHGLQTD-----SCLKQKARRLTILLLLAFYVCFLPFHILRVIR-IESRL---LSI-SCSIENQIHE-----------AYIVSRPLAALNTFGNLLLYVVVSDNFQQAVCSTVRCKVSGNLEQAKKISYSNNP----------------------------------------------------------

GPR81 --------------------------------MAWNATCKNWLAAEAALEKYYLSIFYGIEFVVGVLGNTIVVYGYIFS-LKNWN--SSNIYLFNLSVSDLAFLCTLPMLIRSYANG-NWIYGDVLCISNRYV----LHANLYTSILFLTFISIDRYLIIKYPFRE---------------H-LLQKKEFAILISLAIWVLVTLELLPIL-----PLINPVI--TDNG-TTCND----------FASSGDPNYNLIYSMCLTLLGFL-IPLFVMCFFYYKIALFLKQRNRQVATAL---PLEKPLNLVIMAVVIFSVLFTPYHVMRNVR-IASRL-GSWKQYQCTQV-VINS-----------FYIVTRPLAFLNSVINPVFYFLLGDHFRDMLMNQLRHNFKSLTSFSRWAHELLLSFREK------------------------------------------------------

Group A12

GPR34 MRSHTITMTTTSVSSWPYSSHRMRFITNHSDQPPQNFSATPNVTTCPMDEKLLSTVLTTSYSVIFIVGLVGNIIALYVFLGIHRK--RNSIQIYLLNVAIADLLLIFCLPFRIMYHINQNKWTLGVILCKVVGTLFYMNMYISIILLGFISLDRYIKINRSIQQRKAITTKQSIYVCCIVWMLALGGFLTMIILTLKKG------GHNSTMCFHYRDKHNAKGEAIFNFILVVMFWLIFLLIILSYIKIGKNLLRISKRRSKFPNSGKYATTARNSFIVLIIFTICFVPYHAFRFIYISSQLN-VSSCYWKEIVHKTNEIMLVLSSFNSCLDPVMYFLMSSNIRKIMCQLLFRRFQGEPSRSESTSEFKPGYSLHDTSVAVKIQSSSKST

H963 -------------------------------MTNSSF-------FCPVYKDLEP--FTYFFYLVFLVGIIGSCFATWAFIQKNTN--HRCVSIYLINLLTADFLLTLALPVKIVVDLGVAPWKLKIFHCQVTACLIYINMYLSIIFLAFVSIDRCLQLTHSCKIYRIQEPGFAKMISTVVWLMVLLIMVPNMMIPIKDIK-----EKSNVGCMEFKKEFGRNWHLLTNFICVAIFLNFSAIILISNCLVIRQLYRNKDN----ENYPNVKKALINILLVTTGYIICFVPYHIVRIPYTLSQTEVITDCSTRISLFKAKEATLLLAVSNLCFDPILYYHLSKAFRSKVTETFASPKETKAQKEKLRCENNA--------------------

P2Y12 ----------------------MQAVDNLTSAPGNTS-------LCTRDYKITQVLFPLLYTVLFFVGLITNGLAMRIFFQIRS---KSNFIIFLKNTVISDLLMILTFPFKILSDAKLGTGPLRTFVCQVTSVIFYFTMYISISFLGLITIDRYQKTTRPFKTSNPKNLLGAKILSVVIWAFMFLLSLPNMILTNRQPR-----DKNVKKCSFLKSEFGLVWHEIVNYICQVIFWINFLIVIVCYTLITKELYRSYVRT-RGVGKVPRKKVNVKVFIIIAVFFICFVPFHFARIPYTLSQTRDVFDCTAENTLFYVKESTLWLTSLNACLDPFIYFFLCKSFRNSLISMLKCPNSATSLSQDNRKKEQDGGDPNEETPM----------

KI01 -----------------------MINSTSTQPPDES---------CSQNLLITQQIIPVLYCMVFIAGILLNGVSGWIFFYVPS---SKSFIIYLKNIVIADFVMSLTFPFKILGDSGLGPWQLNVFVCRVSAVLFYVNMYVSIVFFGLISFDRYYKIVKPLWTSFIQSVSYSKLLSVIVWMLMLLLAVPNIILTNQSVR-----EVTQIKCIELKSELGRKWHKASNYIFVAIFWIVFLLLIVFYTAITKKIFKSHLKS-SRNSTSVKKKSSRNIFSIVFVFFVCFVPYHIARIPYTKSQTEAHYSCQSKEILRYMKEFTLLLSAANVCLDPIIYFFLCQPFREILCKKLHIPLKAQNDLDISRIKRG--NTTLESTDTL---------

PAFR ------------------------------MEPHDS---------SHMDSEFRYTLFPIVYSIIFVLGVIANGYVLWVFARLYPCKKFNEIKIFMVNLTMADMLFLITLPLWIVYYQNQGNWILPKFLCNVAGCLFFINTYCSVAFLGVITYNRFQAVTRPIKTAQANTRKRGISLSLVIWVAIVGAASYFLILDSTNTVPDSAGSGNVTRCFEHYEK-GSVPVLIIHIFIVFSFFLVFLIILFCNLVIIRTLLMQPVQ--QQRNAEVKRRALWMVCTVLAVFIICFVPHHVVQLPWTLAELG-FQDSKFHQAINDAHQVTLCLLSTNCVLDPVIYCFLTKKFRKHLTEKFYSMRSSRKCSRATTDTVTEVVVPFNQIPGNSLKN-----

GPR86 ------------------------MNTTVMQGFNRSE-------RCPRDTRIVQLVFPALYTVVFLTGILLNTLALWVFVHIPS---SSTFIIYLKNTLVADLIMTLMLPFKILSDSHLAPWQLRAFVCRFSSVIFYETMYVGIVLLGLIAFDRFLKIIRPLRNIFLKKPVFAKTVSIFIWFFLFFISLPNMILSNKEAT-----PSSVKKCASLKGPLGLKWHQMVNNICQFIFWTVFILMLVFYVVIAKKVYDSYRKS-KSKDRKNNKKLEGKVFVVVAVFFVCFAPFHFARVPYTHSQTNNKTDCRLQNQLFIAKETTLFLAATNICMDPLIYIFLCKKFTEKLPCMQGRKTTAS--SQENHSSQTDNITLG---------------

GPR87 ---MGFNLTLAKLPNNELHGQESHNSGNRSDGPGKN---------TTLHNEFDTIVLPVLYLIIFVASILLNGLAVWIFFHIRN---KTSFIFYLKNIVVADLIMTLTFPFRIVHDAGFGPWYFKFILCRYTSVLFYANMYTSIVFLGLISIDRYLKVVKPFGDSRMYSITFTKVLSVCVWVIMAVLSLPNIILTNGQPT-----EDNIHDCSKLKSPLGVKWHTAVTYVNSCLFVAVLVILIGCYIAISRYIHKSSRQ--FISQSSRKRKHNQSIRVVVAVFFTCFLPYHLCRIPFTFSHLDRLLDESAQKILYYCKEITLFLSACNVCLDPIIYFFMCRSFSRRLFKKSNIRTRSESIRSLQSVRRSEVRIYYDYTDV----------

Group A13

EDG1 -----------------------------------------------------MGPTSVPLVKAHRSS--------VSDYVNYDIIVRH-YNYTGKLNISADKE---NS-I-----KLTSVVFILICCFIILENIFVLLTIWKTKKFHR-PMYYFIGNLALSDLLAGVAYTANLLLSGATT-------YKLTPAQWFLREGSMFVALSASVFSLLAIAIERYITMLK-MKLHNGSNNFRLFLLISACWVISLILGGLPIMGWNCISALSSCSTVLPLYHKHYILFCTTVFTLLLLSIVILYCRIYSLVRTRSRRL---------TFRKNIS------KASRSSENVALLKTVIIVLSVFIACWAPLFILLLLDVGCKVKTCDILFRAEYFLVLA--VLNSGTNPIIYTLTNKEMRRAFIRIMSC-CKCPSGDS---------AGKFKRPIIAGMEFSRSKS---DNSSHPQKDEGDNPETIMSSGNVNSSS---------

EDG2 ------------------------------------------------MAAISTSIPVISQPQFTAMN--------EPQCFYNESIAFF-YNRSGKHLATEWN-----TVS-----KLVMGLGITVCIFIMLANLLVMVAIYVNRRFHF-PIYYLMANLAAADFFAGLAYFYLMFNTGPNT-------RRLTVSTWLLRQGLIDTSLTASVANLLAIAIERHITVFR-MQLHTRMSNRRVVVVIVVIWTMAIVMGAIPSVGWNCICDIENCSNMAPLYSDSYLVFWAIFNLVTFVVMVVLYAHIFGYVRQRTMRM---------SRHSSGP------RRNRD-TMMSLLKTVVIVLGAFIICWTPGLVLLLLD--VCCPQCDVLAYEKFFLLLA--EFNSAMNPIIYSYRDKEMSATFRQILCC-Q---------------------RSENPTGPTESSDRSASSLNHTILAG----VHSNDHSVV--------------

EDG3 ------------------------------------------------------------MATALPPR--------LQPVRGNETLREH-YQYVGKLAGRLKEA---SEGS-----TLTTVLFLVICSFIVLENLMVLIAIWKNNKFHN-RMYFFIGNLALCDLLAGIAYKVNILMSGKKT-------FSLSPTVWFLREGSMFVALGASTCSLLAIAIERHLTMIK-MRPYDANKRHRVFLLIGMCWLIAFTLGALPILGWNCLHNLPDCSTILPLYSKKYIAFCISIFTAILVTIVILYARIYFLVKSSSRKV---------ANHNNSE------RS------MALLRTVVIVVSVFIACWSPLFILFLIDVACRVQACPILFKAQWFIVLA--VLNSAMNPVIYTLASKEMRRAFFRLVCN-CLVR-GR-----------GARASPIQPALDPSRSKSSSSNNSSHSPKVKEDLPHTDPSSCIMDKNAALQNGIFCN

EDG4 -----------------------------------------------------------------MVI--------MGQCYYNETIGFF-YNNSGKELSSHWR-----PKD-----VVVVALGLTVSVLVLLTNLLVIAAIASNRRFHQ-PIYYLLGNLAAADLFAGVAYLFLMFHTGPRT-------ARLSLEGWFLRQGLLDTSLTASVATLLAIAVERHRSVMA-VQLHSRLPRGRVVMLIVGVWVAALGLGLLPAHSWHCLCALDRCSRMAPLLSRSYLAVWALSSLLVFLLMVAVYTRIFFYVRRRVQRM---------AEHVSCH------PRYRE-TTLSLVKTVVIILGAFVVCWTPGQVVLLLDG-LGCESCNVLAVEKYFLLLA--EANSLVNAAVYSCRDAEMRRTFRRLLCC-ACL------------------RQSTRESVHYTSSAQGGASTRIMLPEN----GHPLMDSTL--------------

EDG5 ----------------------------------------------------------------MGSL--------YSEYLNPNKVQEH-YNYTKETLETQETT---SR-------QVASAFIVILCCAIVVENLLVLIAVARNSKFHS-AMYLFLGNLAASDLLAGVAFVANTLLSGSVT-------LRLTPVQWFAREGSASITLSASVFSLLAIAIERHVAIAK-VKLYGSDKSCRMLLLIGASWLISLVLGGLPILGWNCLGHLEACSTVLPLYAKHYVLCVVTIFSIILLAIVALYVRIYCVVRSS-------------HADMAAP------QT------LALLKTVTIVLGVFIVCWLPAFSILLLDYACPVHSCPILYKAHYFFAVS--TLNSLLNPVIYTWRSRDLRREVLRPLQC-WRPGVGVQ---------GRRRVGTPGHHLLPLRSSSS-LERGMHMPTS----PTFLEGNTVV-------------

EDG6 -------------------------------------------------------------MNATGTPVAPESCQQLAAGGHSRLIVLH-YNHSGRLAGRGGPE---DGGL-----GALRGLSVAASCLVVLENLLVLAAITSHMRSRR-WVYYCLVNITLSDLLTGAAYLANVLLSGART-------FRLAPAQWFLREGLLFTALAASTFSLLFTAGERFATMVRPVAESGATKTSRVYGFIGLCWLLAALLGMLPLLGWNCLCAFDRCSSLLPLYSKRYILFCLVIFAGVLATIMGLYGAIFRLVQASGQK---------APRPAARR------------KARRLLKTVLMILLAFLVCWGPLFGLLLADVFGS-NLWAQEYLRGMDWILALAVLNSAVNPIIYSFRSREVCRAVLSFLCCGCLRLGMRGP--------GDCLARAVEAHSGASTTDSSLRPRDSFRGSRSLSFRMREPLSSISSVRSI--------

EDG7 ----------------------------------------------------------------------------MNECHYDKHMDFF-YNRSNTDTVDDWTG----TKL-----VIVLCVGTFFCLFIFFSNSLVIAAVIKNRKFHF-PFYYLLANLAAADFFAGIAYVFLMFNTGPVS-------KTLTVNRWFLRQGLLDSSLTASLTNLLVIAVERHMSIMR-MRVHSNLTKKRVTLLILLVWAIAIFMGAVPTLGWNCLCNISACSSLAPIYSRSYLVFWTVSNLMAFLIMVVVYLRIYVYVKRKTNVL---------SPHTSGS------ISRRR-TPMKLMKTVMTVLGAFVVCWTPGLVVLLLDG-LNCRQCGVQHVKRWFLLLA--LLNSVVNPIIYSYKDEDMYGTMKKMICC-FSQE-------------NPERRPSRIPSTVLSRSDTGSQYIEDSISQG----AVCNKSTS---------------

EDG8 ---------------------------------------------------------------MESGL--------LRPAPVSEVIVLH-YNYTGKLRGARYQP---GAGL-----RADAVVCLAVCAFIVLENLAVLLVLGRHPRFHA-PMFLLLGSLTLSDLLAGAAYAANILLSGPLT-------LKLSPALWFAREGGVFVALTASVLSLLAIALERSLTMAR-RGPAPVSSRGRTLAMAAAAWGVSLLLGLLPALGWNCLGRLDACSTVLPLYAKAYVLFCVLAFVGILAAICALYARIYCQVRANARRLPAR----PGTAGTTST------RARRKPRSLALLRTLSVVLLAFVACWGPLFLLLLLDVACPARTCPVLLQADPFLGLA--MANSLLNPIIYTLTNRDLRHALLRLVCC-GRHSCGRDPSGSQQSASAAEASGGLRRCLPPGLDGSFSGSERSSPQRDGLDTSGSTGSPGAPTAARTLVSEPAAD

ACTR ----------------------------------------------------------------------------------MKHIINS-YENINN-TARNNSD---CPRV-----VLPEEIFFTISIVGVLENLIVLLAVFKNKNLQA-PMYFFICSLAISDMLGSLYKILENILIILRNMGYLKPRGSFETTADDIIDSLFVLSLLGSIFSLSVIAADRYITIFHALRYHSIVTMRRTVVVLTVIWTFCTGTGIT--------------MVIFSH-HVPTVITFTSLFPLMLVFILCLYVHMFLLARSHTRKI--------STLPRAN---------------MKGAITLTILLGVFIFCWAPFVLHVLLMTFCPSNPYCACYMSLFQVNGMLIMCNAVIDPFIYAFRSPELRDAFKKMIF--CS--------------------RYW--------------------------------------------------

CB1R MKSILDGLADTTFRTITTDLLYVGSNDIQYEDIKGDMASKLGYFPQKFPLTSFRGSPFQEKMTAGDNPQLVPADQVNITEFYNKSLSSFKENEENIQCGENFMDIECFMVLNPSQQLAIAVLSLTLGTFTVLENLLVLCVILHSRSLRCRPSYHFIGSLAVADLLGSVIFVYSFIDFHVFHR-------KDSRNVFLFKLGGVTASFTASVGSLFLTAIDRYISIHRPLAYKRIVTRPKAVVAFCLMWTIAIVIAVLPLLGWNCEKLQSVCSDIFPHIDETYLMFWIGVTSVLLLFIVYAYMYILWKAHSHAVRMIQRGTQKSIIIHTSEDGKVQVTRPDQARMDIRLAKTLVLILVVLIICWGPLLAIMVYDVFGKMNKLIKTVFAFCSMLC---LLNSTVNPIIYALRSKDLRHAFRSMFPS-CEG----------------TAQPLDNSMGDSDCLHKHANNAASVHRAAESCIKSTVKIAKVTMSVSTDTSAEAL-

CB2R ----------------------------------------------------------------------------MEECWVTEIANGS-KDGLDSNPMKDYMILS-GPQK-----TAVAVLCTLLGLLSALENVAVLYLILSSHQLRRKPSYLFIGSLAGADFLASVVFACSFVNFHVFHG-------VDSKAVFLLKIGSVTMTFTASVGSLLLTAIDRYLCLRYPPSYKALLTRGRALVTLGIMWVLSALVSYLPLMGWTCCPR--PCSELFPLIPNDYLLSWLLFIAFLFSGIIYTYGHVLWKAHQHVASLS-------GHQDRQVPG------MARMRLDVRLAKTLGLVLAVLLICWFPVLALMAHSLATTLSDQVKKAFAFCSMLC---LINSMVNPVIYALRSGEIRSSAHHCLAHWKKCVR-----------------GLGSEAKEEAPRSSVTETEADGKITPWPDSRDLDLSDC---------------

GPR3 ------------------------------------------------------------MMWGAGSP---------LAWLSAGSGNVN-VSSVGPAEGPTGPA-APLPSP-----KAWDVVLCISGTLVSCENALVVAIIVGTPAFRA-PMFLLVGSLAVADLLAGLGLVLHFAAVFCIG----------SAEMSLVLVGVLAMAFTASIGSLLAITVDRYLSLYNALTYYSETTVTRTYVMLALVWGGALGLGLLPVLAWNCLDGLTTCGVVYPL-SKNHLVVLAIAFFMVFGIMLQLYAQICRIVCRHAQQI--------ALQRHLLP-------ASHYVATRKGIATLAVVLGAFAACWLPFTVYCLLGDAHS--PPLYTYLTLLPATY-----NSMINPIIYAFRNQDVQKVLWAVCCC-CS--------------------SSKIPFRSRSPSDV---------------------------------------

GPR6 -------------------------------------MNASAASLNDSQVVVVAAEGAAAAATAAGGPDTGEWGPPAAAALGAGGGANG-SLELSSQLSAGPPG-LLLPAV-----NPWDVLLCVSGTVIAGENALVVALIASTPALRT-PMFVLVGSLATADLLAGCGLILHFVFQYLVP----------SETVSLLTVGFLVASFAASVSSLLAITVDRYLSLYNALTYYSRRTLLGVHLLLAATWTVSLGLGLLPVLGWNCLAERAACSVVRPL-ARSHVALLSAAFFMVFGIMLHLYVRICQVVWRHAHQI--------ALQQHCLA-------PPHLAATRKGVGTLAVVLGTFGASWLPFAIYCVVGSHED--PAVYTYATLLPATY-----NSMINPIIYAFRNQEIQRALWLLLCG-CF--------------------QSKVPFRSRSPSEV---------------------------------------

GPRC -------------------------------------------------------MNEDLKVNLSGLP---------RDYLDAAAAENI-SAAVSSRVPAVEP--EPELVV-----NPWDIVLCTSGTLISCENAIVVLIIFHNPSLRA-PMFLLIGSLALADLLAGIGLITNFVFAYLLQ----------SEATKLVTIGLIVASFSASVCSLLAITVDRYLSLYYALTYHSERTVTFTYVMLVMLWGTSICLGLLPVMGWNCLRDESTCSVVRPL-TKNNAAILSVSFLFMFALMLQLYIQICKIVMRHAHQI--------ALQHHFLA-------TSHYVTTRKGVSTLAIILGTFAACWMPFTLYSLIADYTY--PSIYTYATLLPATY-----NSIINPVIYAFRNQEIQKALCLICCG-CI--------------------PSSLAQRARSPSDV---------------------------------------

MC3R ---------------------------MSIQKKYLEGDFVFPVSSSSFLRTLLEPQLGSALLTAMNAS--------CCLPSVQPTLPNG-SEHLQAPFFSNQSSSAFCEQV-----FIKPEIFLSLGIVSLLENILVILAVVRNGNLHS-PMYFFLCSLAVADMLVSVSNALETIMIAIVHSDYLTFEDQFIQHMDNIFDSMICISLVASICNLLAIAVDRYVTIFYALRYHSIMTVRKALTLIVAIWVCCGVCGVV--------------FIVYSE-SKMVIVCLITMFFAMMLLMGTLYVHMFLFARLHVKRI--------AALPPADG------VAPQQHSCMKGAVTITILLGVFIFCWAPFFLHLVLIITCPTNPYCICYTAHFNTYLVLIMCNSVIDPLIYAFRSLELRNTFREILCG-CN--------------------GMNLG------------------------------------------------

MC4R ----------------------------------------------------------MVNSTHRGMH--------TSLHLWNRSSYRL-HSNASESLGKGYSDGGCYEQL-----FVSPEVFVTLGVISLLENILVIVAIAKNKNLHS-PMYFFICSLAVADMLVSVSNGSETIIITLLN-STDTDAQSFTVNIDNVIDSVICSSLLASICSLLSIAVDRYFTIFYALQYHNIMTVKRVGIIISCIWAACTVSGIL--------------FIIYSD-SSAVIICLITMFFTMLALMASLYVHMFLMARLHIKRI--------AVLPGTG--------AIRQGANMKGAITLTILIGVFVVCWAPFFLHLIFYISCPQNPYCVCFMSHFNLYLILIMCNSIIDPLIYALRSQELRKTFKEIIC--CY--------------------PLGGLCDLSSRY-----------------------------------------

MC5R ----------------------------------------------------------------MNSS--------FHLHFLDLNLNAT-EGNLSGPNVKNKSS--PCEDM-----GIAVEVFLTLGVISLLENILVIGAIVKNKNLHS-PMYFFVCSLAVADMLVSMSSAWETITIYLLNNKHLVIADAFVRHIDNVFDSMICISVVASMCSLLAIAVDRYVTIFYALRYHHIMTARRSGAIIAGIWAFCTGCGIV--------------FILYSE-STYVILCLISMFFAMLFLLVSLYIHMFLLARTHVKRI--------AALPGAS--------SARQRTSMQGAVTVTMLLGVFTVCWAPFFLHLTLMLSCPQNLYCSRFMSHFNMYLILIMCNSVMDPLIYAFRSQEMRKTFKEIIC--CR--------------------GFRIACSFPRRD-----------------------------------------

MSHR ----------------------------------------------------------------MAVQ--------GSQRRLLGSLNST-PTAIPQLGLAANQTGARCLEV-----SISDGLFLSLGLVSLVENALVVATIAKNRNLHS-PMYCFICCLALSDLLVSGSNVLETAVILLLEAGALVARAAVLQQLDNVIDVITCSSMLSSLCFLGAIAVDRYISIFYALRYHSIVTLPRARRAVAAIWVASVVFSTL--------------FIAYYD-HVAVLLCLVVFFLAMLVLMAVLYVHMLARACQHAQGI--------ARLHKRQR-------PVHQGFGLKGAVTLTILLGIFFLCWGPFFLHLTLIVLCPEHPTCGCIFKNFNLFLALIICNAIIDPLIYAFHSQELRRTLKEVLT--CS--------------------W----------------------------------------------------

Group A14

PD2R_HUMA --------------------------MKSPFYRCQNTTS-----VE-KGNSAVMGGVLFSTGLLGNLLALGLLARSGL--GWCSRRPLRPLPSVFYMLVCGLTVTDLLGKCLLSPVVLAAYAQNRSLRVLAPALDNSLCQAFAFFMSFFGLSSTLQLLAMALECWLSLGHPFFYRRHITLRLGALVAPVVSAFSLAFCALPFMGFGKFVQYCPGTWCFIQMVHEEGSLS------VLGYSVLYSSLMALLVLATVLCNLGAMRNLYAMHRR-LQR---H------------------------PRSCTRDCAEPRADGREASPQPLEELDHLLLLALMTVLFTMCSLPVIYRAYYGAFKDVK------EKNRTSEEAEDLRALRFLSVISIVDPWIFIIFRSPVFRIFFHKIFIRPLRYRSRCSNSTNMESSL---------------------------------------------------------------------------------------------------------------------------

PE21_HUMA ----------------MSPCGPLNLSLAGEATTCAAPWVPNTSAVPPSGASPALPIFSMTLGAVSNLLALALLAQAAG------RLRRRRSATTFLLFVASLLATDLAGHVIPGALVLRLYTAGR-----APA--GGACHFLGGCMVFFGLCPLLLGCGMAVERCVGVTRPLLHAARVSVARARLALAAVAAVALAVALLPLARVGRYELQYPGTWCFIGLGPPGG-------WRQALLAGLFASLGLVALLAALVCNTLSGLALHRARWRRRSRRPPPASGPDSRRRWGAHGPRSASASSASSIASASTFFGGSRSSGSARRARAHDVEMVGQLVGIMVVSCICWSPMLVLVALAV---------GGWSSTSLQ-RPLFLAVRLASWNQILDPWVYILLRQAVLRQLLRLLPPRAGAKGGPAGLGLTPSAWEASSLRSSRHSGLSHF------------------------------------------------------------------------------------------------------------

PE22_HUMA ---------------------------MGNASNDSQSEDCETRQWLPPGESPAISSVMFSAGVLGNLIALALLARRWRGDVGCS-AGRRSSLSLFHVLVTELVFTDLLGTCLISPVVLASYARNQTLVALAPE-S-RACTYFAFAMTFFSLATMLMLFAMALERYLSIGHPYFYQRRVSASGGLAVLPVIYAVSLLFCSLPLLDYGQYVQYCPGTWCFIRHG-----RT--------AYLQLYATLLLLLIVSVLACNFSVILNLIRMHRR-SRR---S----------------------RCGPSLGSGRGGPGARRRGERVSMAEETDHLILLAIMTITFAVCSLPFTIFAYMNETS------------SRKE-KWDLQALRFLSINSIIDPWVFAILRPPVLRLMRSVL---CCRISLRTQDATQTSCSTQSDASKQADL-----------------------------------------------------------------------------------------------------------------

PE23_HUMA MKETRGYGGDAPFCTRLNHSYTGMWAPERSAEARGNLTRPPGSGEDCGSVSVAFPITMLLTGFVGNALAMLLVSRS-------YRRRESKRKKSFLLCIGWLALTDLVGQLLTTPVVIVVYLSKQRWEHIDPS--GRLCTFFGLTMTVFGLSSLFIASAMAVERALAIRAPHWYASHMKTRATRAVLLGVWLAVLAFALLPVLGVGQYTVQWPGTWCFISTGRGGNGTSSSHNWGNLFFASAFAFLGLLALTVTFSCNLATIKALVSRCRAKATASQSS------------------------------------------AQWGRITTETAIQLMGIMCVLSVCWSPLLIMMLKMIFNQTSVEHCKTHTEKQKECNFFLIAVRLASLNQILDPWVYLLLRKILLRKFCQIRYHTNNYASSSTSLPCQCSSTLMWSDHLER-------------------------------------------------------------------------------------------------------------------

PE24_HUMA -------------------------------MSTPGVNSSASLSPDRLNSPVTIPAVMFIFGVVGNLVAIVVLCKS----------RKEQKETTFYTLVCGLAVTDLLGTLLVSPVTIATYMKGQ-----WPG-GQPLCEYSTFILLFFSLSGLSIICAMSVERYLAINHAYFYSHYVDKRLAGLTLFAVYASNVLFCALPNMGLGSSRLQYPDTWCFIDWTTNVTAHA--------AYSYMYAGFSSFLILATVLCNVLVCGALLRMHRQ-FMRRTSL-----GTEQHHAAAAASVASRGHPAASPALPRLSDFRRRRSFRRIAGAEIQMVILLIATSLVVLICSIPLVVRVFVNQLYQPS-------LEREVSKNPDLQAIRIASVNPILDPWIYILLRKTVLSKAIEKIKCLFCRIGGSRRERSGQHCSDSQRTSSAMSGHSRSFISRELKEISSTSQTLLPDLSLPDLSENGLGGRNLLPGVPGMGLAQEDTTSLRTLRISETSDSSQGQDSESVLLVDEAGGSGRAGPAPKGSSLQVTFPSETLNLSEKCI

PF2R_HUMA ----------------------MSMNNSKQLVSPAAALLSNTTCQTENRLSVFFSVIFMTVGILSNSLAIAILMKA-------YQRFRQKSKASFLLLASGLVITDFFGHLINGAIAVFVYASDKEWIRFDQS--NVLCSIFGICMVFSGLCPLLLGSVMAIERCIGVTKPIFHSTKITSKHVKMMLSGVCLFAVFIALLPILGHRDYKIQASRTWCFYNTEDIK-------DWEDRFYLLLFSFLGLLALGVSLLCNAITGITLLRVKFKSQQH----------------------------------------------RQGRSHHLEMVIQLLAIMCVSCICWSPFLVTMANIGIN--------GNHSLETC-ETTLFALRMATWNQILDPWVYILLRKAVLKNLYKLASQCCGVHVISLHIWELSSIKNSLKVAAISESPVAEKSAST--------------------------------------------------------------------------------------------------------

PI2R_HUMA -----------------------------------MADSCRNLTYVRGSVGPATSTLMFVAGVVGNGLALGILS-----------ARRPARPSAFAVLVTGLAATDLLGTSFLSPAVFVAYARNSSLLGLARG-GPALCDAFAFAMTFFGLASMLILFAMAVERCLALSHPYLYAQLDGPRCARLALPAIYAFCVLFCALPLLGLGQHQQYCPGSWCFLRMRWAQPGGA--------AFSLAYAGLVALLVAAIFLCNGSVTLSLCRMYRQ-QKR---H-----------------------------------QGSLGPRPRTGEDEVDHLILLALMTVVMAVCSLPLTIRCFTQAVAPDS-----------SSEMGDLLAFRFYAFNPILDPWVFILFRKAVFQRLKLWVCCLCLGPAHGDSQTPLSQLASGRRDPRAPSAPVGKEGSCVPLSAWGEGQVEPLPPTQQSSGSAVGTSSKAEASVACSLC-----------------------------------------------------------------

TA2R_HUMA ------------------------MWPNGSSLGPCFRPTNITLEERRLIASPWFAASFCVVGLASNLLALSVLAGA--------RQGGSHTRSSFLTFLCGLVLTDFLGLLVTGTIVVSQHAALFEWHAVDPG--CRLCRFMGVVMIFFGLSPLLLGAAMASERYLGITRPFSRPAVASQRRAWATVGLVWAAALALGLLPLLGVGRYTVQYPGSWCFLTLGAES---------GDVAFGLLFSMLGGLSVGLSFLLNTVSVATLCHVYHGQEAAQ---------------------------------------------QRPRDSEVEMMAQLLGIMVVASVCWLPLLVFIAQTVLRNPPAMSPAGQLSRTTE-KELLIYLRVATWNQILDPWVYILFRRAVLRRLQPRLSTRPRRVSLCGPAWSTVARSRLTATSASRVQAILVPQPPEQLGLQA--------------------------------------------------------------------------------------------------

Group A15

EBI2 -----------------------------------------------------------------MDIQMANNFTPPSATP--QGNDCDLYAHHSTARIVMP-------LHYSLVFIIGLVGNLLALVVIVQNRK----KINSTTLYSTNLVISDILFTTALPTRIAYYAMGFDWRIGDALCRITALV-FYINTYAGVNFMTCLSIDRFIAVVHPLRYNKIKRIEHAKGVCIFVWILVFAQTLPLLINPMSKQ------EAERITCMEYPNFEETKSLPW--ILLGACFIGYVLPLIIILICYSQICCKLFRTAK-QNPLTEKS-GVNKKALNTIILIIVVFVLCFTPYHVAIIQHMIKKLRFSN--FLECSQRHSFQISLHFTVCLMNFNCCMDPFIYFFACKGYKRKVMRMLK------RQVSVSISSAVKSAPEENSREMTETQMMIHSKSSNGK------------------------------

G2A ----------------------MCPMLLKNGYNGNAT-------------------------------PVTTTAPWASLGL--SAKTCNNV-SFEESRIVLV-------VVYSAVCTLGVPANCLTAWLALLQVL----QGNVLAVYLLCLALCELLYTGTLPLWVIYIRNQHRWTLGLLACKVTAYI-FFCNIYVSILFLCCISCDRFVAVVYALESRGRRRRRTAILISACIFILVGIVHYPVFQTEDKE------------TCFDMLQMDSRIAGYY----YARFTVGFAIPLSIIAFTNH----RIFRSIK-QSMGLSAA--QKAKVKHSAIAVVVIFLVCFAPYHLVLLVKAAAFSYYRGDRNAMCGLEERLYTASVVFLCLSTVNGVADPIIYVLATDHSRQEVSRIHKGWKEWSMKTDVTRLTHSRDTE-ELQSPVALADHYTFSRPVHPPGSPCPAKRLIEESC----------------

GPR35 ---------------------------------------------------------------------MNGTYNTCG--------SSDLT-WPPAIKLGFY-------AYLGVLLVLGLLLNSLALWVFCCRMQ----QWTETRIYMTNLAVADLCLLCTLPFVLHSLRDT----SDTPLCQLSQGI-YLTNRYMSISLVTAIAVDRYVAVRHPLRARGLRSPRQAAAVCAVLWVLVIGSLVARWLLGIQE-------GG---FCFRSTRHNFNSMRFP--------LLGFYLPLAVVVFCSL----KVVTALAQRPPTDVGQAEATRKAARMVWANLLVFVVCFLPLHVGLTVRLAVGWN-------ACALLETIRRALYITSKLSDANCCLDAICYYYMAKEFQEASAL-----------AVAPRAKAHKSQ---DSLCVTLA------------------------------------------

GPR4 ----------------------------------------------------------------------MGNHTWEG---------C-HV-DSRVDHLFPP-------SLYIFVIGVGLPTNCLALWAAYRQVQ----QRNELGVYLMNLSIADLLYICTLPLWVDYFLHHDNWIHGPGSCKLFGFI-FYTNIYISIAFLCCISVDRYLAVAHPLRFARLRRVKTAVAVSSVVWATELGANSAPLFHDELFR-----DRYNHTFCFEKFPMEGWVAWMN----LYRVFVGFLFPWALMLLSYR----GILRAVR-GSVSTERQ--EKAKIKRLALSLIAIVLVCFAPYHVLLLSRSAIYLGR----PWDCGFEERVFSAYHSSLAFTSLNCVADPILYCLVNEGARSDVAKA--------LHNLLRFLASDKPQE-MANASLTLETPLTS-KRNSTAKAMTGSWAATPPSQG-DQVQLKMLPPAQ--

GPR55 ----------------------------------------------------------------------MSQQNTSG--------DCLFDGVNELMKTLQF-------AVHIPTFVLGLLLNLLAIHGFSTFLKNRWPDYAATSIYMINLAVFDLLLVLSLPFKMVLSQVQ---SPFPSLCTLVECL-YFVSMYGSVFTICFISMDRFLAIRYPLLVSHSGPPGRSLGSACTIWVLVWTGSIPIYSFHGKV--------EK-YMCFHNMSDDTWSAKVF----FPLEVFGFLLPMGIMGFCCS----RSIHILLGRRDHTQDW-VQQKACIYSIAASLAVFVVSFLPVHLGFFLQFLVRNSF----IVECRAKQSISFFLQLSMCFSNVNCCLDVFCYYFVIKEFRMNIRAH----------RPSRVQLVLQDTT-ISRG-----------------------------------------------

GPR65 ----------------------------------------------------------------------MNSTCIEE--------------QHDLDHYLFP-------IVYIFVIIVSIPANIGSLCVSFLQPK----KESELGIYLFSLSLSDLLYALTLPLWIDYTWNKDNWTFSPALCKGSAFL-MYMKFYSSTAFLTCIAVDRYLAVVYPLKFFFLRTRRIALMVSLSIWILETIFNAVMLWEDETVVEYCDAEKSNFTLCYDKYPLEKWQINLN----LFRTCTGYAIPLVTILICNR----KVYQAVR-HNKATENK--EKKRIIKLLVSITVTFVLCFTPFHVMLLIRCILEHAVNF--EDHSNSGKRTYTMYRITVALTSLNCVADPILYCFVTETGRYDMWNI--------LKFCTGRCNTSQRQ---RKRILSVSTKDTMELEVLE-------------------------------

GPR68 ----------------------------------------------------------------------MGNITADNS-----SMSC-TI-DHTIHQTLAP-------VVYVTVLVVGFPANCLSLYFGYLQIK----ARNELGVYLCNLTVADLFYICSLPFWLQYVLQHDNWSHGDLSCQVCGIL-LYENIYISVGFLCCISVDRYLAVAHPFRFHQFRTLKAAVGVSVVIWAKELLTSIYFLMHEEVIE-----DENQHRVCFEHYPIQAWQRAIN----YYRFLVGFLFPICLLLASYQ----GILRAVR-RSHGTQKS--RKDQIQRLVLSTVVIFLACFLPYHVLLLVRSVWEAS--------CDFAKGVFNAYHFSLLLTSFNCVADPVLYCFVSETTHRDLARL--------RGACLAFLTCSRTG--RAREAYPLGAPEASGKSGAQGEEPELLTKLHPAFQTPNSPGSGGFPTGRLA

GPR92 --------------------------------------------------------------------MLANSSSTNSS-----VLPC--P-DYRPTHRLHL-------VVYSLVLAAGLPLNALALWVFLRALR----VHSVVSVYMCNLAASDLLFTLSLPVRLSYYALH-HWPFPDLLCQTTGAI-FQMNMYGSCIFLMLINVDRYAAIVHPLRLRHLRRPRVARLLCLGVWALILVFAVPAARVHRPSR--CRYRDLEVRLCFESFSDELWKGRLLP-LVLLAEALGFLLPLAAVVYSSG----RVFWTLA-RPDATQSQ--RRRKTVRLLLANLVIFLLCFVPYNSTLAVYGLLRSKLV---AASVPARDRVRGVLMVMVLLAGANCVLDPLVYYFSAEGFRNTLRGLGT-----PHRARTSATNGTRAAL-AQSERSAVTTDATRPDAASQGLLRPSDSHSLSSFTQCPQDSAL--------

GPRH -----------------------------MNGLEVAP-------------------------------PGLITNFSLAT-----AEQC-GQ-ETPLENMLFA-------SFYLLDFILALVGNTLALWLFIRDHK----SGTPANVFLMHLAVADLSCVLVLPTRLVYHFSGNHWPFGEIACRLTGFL-FYLNMYASIYFLTCISADRFLAIVHPVKSLKLRRPLYAHLACAFLWVVVAVAMAPLLVSPQTVQ------TNHTVVCLQLYREKASHHALV------SLAVAFTFPFITTVTCYL----LIIRSLR-QGLRVEKR--LKTKAVRMIAIVLAIFLVCFVPYHVNRSVYVLHYRSHG----ASCATQRILALANRITSCLTSLNGALDPIMYFFVAEKFRHALCN----------LLCGKRLKGPPPS---FEGKTNESSLSAKSEL----------------------------------

GPRI ----------------------------------------------------------------------MITLNNQDQ-------PVTFNSSHPDEYKIAA------LVFYSCIFIIGLFVNITALWVFSCTTK----KRTTVTIYMMNVALVDLIFIMTLPFRMFYYAKD-AWPFGEYFCQIIGAL-TVFYPSIALWLLAFISADRYMAIVQPKYAKELKNTCKAVLACVGVWIMTLTTTTPLLLLYKDPD-----KDSTPATCLKISDIIYLKAVNVLN--LTRLTFFFLIPLFIMIGCYL----VIIHNLL-HGRTSKLKPKVKEKSIRIIITLLVQVLVCFMPFHICFAFLMLGTGE------------NSYNPWGAFTTFLMNLSTCLDVILYYIVSKQFQARVISV--------MLYRNYLRSLRRKS---FRSGSLRSLSNINSEML---------------------------------

GPRK -------------------MPSVSPAGPSAGAVPNAT-------------------------------AVTTVRTNASGLE--VPLFHLFARLDEELHGTFPGLCVALMAVHGAIFLAGLVLNGLALYVFCCRTR----AKTPSVIYTINLVVTDLLVGLSLPTRFAVYYGA----RGCLRCAFPHVLGYFLNMHCSILFLTCICVDRYLAIVRPEAPAACRQPACARAVCAFVWLAAGAVTLSVLGVTGSR------------PCCRVFAL-------------T--VLEFLLPLLVISVFTG----RIMCALS-RPGLLHQGRQRRVRAMQLLLTVLIIFLVCFTPFHARQVAVALWPDMP----------HHTSLVVYHVAVTLSSLNSCMDPIVYCFVTSGFQATVRGLFGQHGE-REPSSGDVVSMHRSSKGSGRHHILSAGPHALTQALANGPEA---------------------------

P2Y5 -----------------------------------------------------------------------MVSVNSS--------HC-FY-NDSFKYTLYG-------CMFSMVFVLGLVSNCVAIYIFICVLK----VRNETTTYMINLAMSDLLFVFTLPFRIFYFTTR-NWPFGDLLCKISVML-FYTNMYGSILFLTCISVDRFLAIVYPFKSKTLRTKRNAKIVCTGVWLTVIGGSAPAVFVQSTHSQ----GNNASEACFENFPEATWKTYLSR-IVIFIEIVGFFIPLILNVTCSS----MVLKTLT-KPVTLSRSKINKTKVLKMIFVHLIIFCFCFVPYNINLILYSLVRTQT----FVNCSVVAAVRTMYPITLCIAVSNCCFDPIVYYFTSDTIQNSIKMK--------NWSVRRSDFRFSEVH-GAENFIQHNLQTLKSKIFDNESAA---------------------------

P2Y9 ------------------MGDRRFIDFQFQDSNSSLR-------------------------------PRLGNATANN--------TC-IV-DDSFKYNLNG-------AVYSVVFILGLITNSVSLFVFCFRMK----MRSETAIFITNLAVSDLLFVCTLPFKIFYNFNR-HWPFGDTLCKISGTA-FLTNIYGSMLFLTCISVDRFLAIVYPFRSRTIRTRRNSAIVCAGVWILVLSGGISASLFSTTN------VNNATTTCFEGFSKRVWKTYLSK-ITIFIEVVGFIIPLILNVSCSS----VVLRTLR-KPATLSQIGTNKKKVLKMITVHMAVFVVCFVPYNSVLFLYALVRSQA----ITNCFLERFAKIMYPITLCLATLNCCFDPFIYYFTLESFQKSFYIN--------AHIRMESLFKTETPL-TTKPSLPAIQEEVSDQTTNNGGELMLESTF---------------------

P2Y10 ---------------------------MANLDKYTET-------------------------------FKMGSNSTSTA-----EIYCNVT-NVKFQYSLYA-------TTYILIFIPGLLANSAALWVLCRFIS----KKNKAIIFMINLSVADLAHVLSLPLRIYYYISH-HWPFQRALCLLCFYL-KYLNMYASICFLTCISLQRCFFLLKPFRARDWKR-RYDVGISAAIWIVVGTACLPFPILRSTD-------LNNNKSCFADLGYKQMNAVALVGMITVAELAGFVIPVIIIAWCTW----KTTISLR-QPPMAFQGISERQKALRMVFMCAAVFFICFTPYHINFIFYTMVKETI----ISSCPVVRIALYFHPFCLCLASLCCLLDPILYYFMASEFRDQLSRH--------GSSVTRSRLMSKES---GSSMIG--------------------------------------------

PAR1 MGPRRLLLVAACFSLCGPLLSARTRARRPESKATNATLDPRSFLLRNPNDKYEPFWEDEEKNESGLTEYRLVSINKSSP-L--QKQLPAFISEDASGYLTSSWLTLFVPSVYTGVFVVSLPLNIMAIVVFILKMK----VKKPAVVYMLHLATADVLFVSVLPFKISYYFSGSDWQFGSELCRFVTAA-FYCNMYASILLMTVISIDRFLAVVYPMQSLSWRTLGRASFTCLAIWALAIAGVVPLVLKEQTIQVP----GLNITTCHDVLNETLLEGYYAY-YFSAFSAVFFFVPLIISTVCYV----SIIRCLS-SSAVANRS--KKSRALFLSAAVFCIFIICFGPTNVLLIAHYSFLSHT--------STTEAAYFAYLLCVCVSSISSCIDPLIYYYASSECQRYVYS----------ILCCKESSDPSSYN--SSGQLMASKMDTCSSNLNNSIYKKLLT-----------------------

PAR2 ---MRSPSAAWLLGAAILLAASLSCSGTIQGTNRSSKGR-------------------------SLIGKVDGTSHVTGKGV--TVETVFSVDEFSASVLTGKLTTVFLPIVYTIVFVVGLPSNGMALWVFLFRTK----KKHPAVIYMANLALADLLSVIWFPLKIAYHIHGNNWIYGEALCNVLIGF-FYGNMYCSILFMTCLSVQRYWVIVNPMG-HSRKKANIAIGISLAIWLLILLVTIPLYVVKQTIFIP----ALNITTCHDVLPEQLLVGDMFN-YFLSLAIGVFLFPAFLTASAYV----LMIRMLR-SSAMDENSEKKRKRAIKLIVTVLAMYLICFTPSNLLLVVHYFLIKS---------QGQSHVYALYIVALCLSTLNSCIDPFVYYFVSHDFRDHAKN---------ALLCRSVRTVKQMQVSLTSKKHSRKSSSYSSSSTTVKTSY---------------------------

PAR3 -----MKALIFAAAGLLLLLPTFCQSGMENDTNNLAKPTLPIKTFRGAPPN------SFEEFPFSALEGWTGATITVKIKCPEESASHLHVKNATMGYLTSSLSTKLIPAIYLLVFVVGVPANAVTLWMLFFRTR-----SICTTVFYTNLAIADFLFCVTLPFKIAYHLNGNNWVFGEVLCRATTVI-FYGNMYCSILLLACISINRYLAIVHPFTYRGLPKHTYALVTCGLVWATVFLYMLPFFILKQEYY----LVQPDITTCHDVHNTCESSSPFQLYYFISLAFFGFLIPFVLIIYCYA----AIIRTLN---AYDHRW----LWYVKASLLILVIFTICFAPSNIILIIHHANYYYN---------NTDGLYFIYLIALCLGSLNSCLDPFLYFLMSKTRNHSTAY------------LTK-------------------------------------------------------------

Group A16

OPSB_HUMA -------------------MRKMSEEEFYLFKNISS---VGPWDGPQYHIAPVWAFYLQAAFMGTVF--LIGFPLNAMVLVATLRYKKLRQPLNYILVNVSFGGFLLCIFSVFPVFVASCNGYFVFGRHVCALEGFLGTVAGLVTGWSLAFLAFERYIVICKPFGNFRFSSKHALTVVLATWTIGIGVSIPPFFGWSRFIPEGLQCSCGPDWYTVGTKYRSESYTWFLFIFCFIVPLSLICFSYTQLLRALKA-VAAQQQESATTQKAER-EVSRMVVVMVGSFCVCYVPYAAFAMYMVNNRNHGLDLRLVTIPSFFSKSACIYNPIIYCFMNKQFQACIMKMV-CGKA-MTDESDTCSSQKTEVSTVSSTQVGPN

OPSD_HUMA -------------------MNGTEGPNFYVPFSNATGVVRSPFEYPQYYLAEPWQFSMLAAYMFLLI--VLGFPINFLTLYVTVQHKKLRTPLNYILLNLAVADLFMVLGGFTSTLYTSLHGYFVFGPTGCNLEGFFATLGGEIALWSLVVLAIERYVVVCKPMSNFRFGENHAIMGVAFTWVMALACAAPPLAGWSRYIPEGLQCSCGIDYYTLKPEVNNESFVIYMFVVHFTIPMIIIFFCYGQLVFTVKE-AAAQQQESATTQKAEK-EVTRMVIIMVIAFLICWVPYASVAFYIFTHQGSNFGPIFMTIPAFFAKSAAIYNPVIYIMMNKQFRNCMLTTICCGKNPLGDDEASATVSKTETSQVAPA-----

OPSG_HUMA MAQQWSLQRLAGRHPQDSYEDSTQSSIFTYTNSNST---RGPFEGPNYHIAPRWVYHLTSVWMIFVV--IASVFTNGLVLAATMKFKKLRHPLNWILVNLAVADLAETVIASTISVVNQVYGYFVLGHPMCVLEGYTVSLCGITGLWSLAIISWERWMVVCKPFGNVRFDAKLAIVGIAFSWIWAAVWTAPPIFGWSRYWPHGLKTSCGPDVFSGSSYPGVQSYMIVLMVTCCITPLSIIVLCYLQVWLAIRA-VAKQQKESESTQKAEK-EVTRMVVVMVLAFCFCWGPYAFFACFAAANPGYPFHPLMAALPAFFAKSATIYNPVIYVFMNRQFRNCILQLF--GKK-VDDGSELSSASKTEVSSVSSVSPA--

OPSR_HUMA MAQQWSLQRLAGRHPQDSYEDSTQSSIFTYTNSNST---RGPFEGPNYHIAPRWVYHLTSVWMIFVV--TASVFTNGLVLAATMKFKKLRHPLNWILVNLAVADLAETVIASTISIVNQVSGYFVLGHPMCVLEGYTVSLCGITGLWSLAIISWERWLVVCKPFGNVRFDAKLAIVGIAFSWIWSAVWTAPPIFGWSRYWPHGLKTSCGPDVFSGSSYPGVQSYMIVLMVTCCIIPLAIIMLCYLQVWLAIRA-VAKQQKESESTQKAEK-EVTRMVVVMIFAYCVCWGPYTFFACFAAANPGYAFHPLMAALPAYFAKSATIYNPVIYVFMNRQFRNCILQLF--GKK-VDDGSELSSASKTEVSSVSSVSPA--

OPSX_HUMA -------------------------------MLRNNLGNSSDSKNEDGSVFSQTEHNIVATYLIMAG--MISIISNIIVLGIFIKYKELRTPTNAIIINLAVTDIGVSSIGYPMSAASDLYGSWKFGYAGCQVYAGLNIFFGMASIGLLTVVAVDRYLTICLPDVGRRMTTNTYIGLILGAWINGLFWALMPIIGWASYAPDPTGATCTINWRKNDR--SFVSYTMTVIAINFIVPLTVMFYCYYHVTLSIKHHTTSDCTESLNRDWSDQIDVTKMSVIMICMFLVAWSPYSIVCLWASFGDPKKIPPPMAIIAPLFAKSSTFYNPCIYVVANKKFRRAMLAMFKCQTHQTMPVTSILPMDVSQNPLASGRI----

RGR_HUMAN ------------------------------------------MAETSALPTGFGELEVLAVGMVLLVEALSGLSLNTLTIFSFCKTPELRTPCHLLVLSLALADSGISLNA-LVAATSSLLRRWPYGSDGCQAHGFQGFVTALASICSSAAIAWGRYHHYCT---RSQLAWNSAVSLVLFVWLSSAFWAALPLLGWGHYDYEPLGTCCTLDYSKGDR--NFTSFLFTMSFFNFAMPLFITITSYSLM------------EQKLGKSGHLQVNTT----LPARTLLLGWGPYAILYLYAVIADVTSISPKLQMVPALIAKMVPTINAINYALGNEMVCRGIWQCLSPQKREKDRTK---------------------

Group A17

5H2A_HUMA ----------------------MDILCEENTSLSSTTNSLMQLNDDTRLYSNDFNSGEANTSDAFNWTVDSENRTNLSCEGCLSPSCLSLLHLQEKNWSALLTAVVIILTIAGNILVIMAVSLEKKLQN-ATNYFLMSLAIADMLLGFLVMPVSMLTILYGYRWPLPSKLCAVWIYLDVLFSTASIMHLCAISLDRYVAIQNPIHHSRFNSR---TKAFLKIIAVWTISVGISMPIPVFGLQDDS-------------------------KVFKEGSCLL-ADD--NFVLIGSFVSFFIPLTIMVITYFLTIKSLQKEATLCVS--------------------------------------------------------------------------------------------------------------------DLGTRAKLASFSFLPQSSLSSEKLFQRSIHREPGSYTGRRTMQSISNEQKACKVLGIVFFLFVVMWCPFFITNIMAVICKESCN------EDVIGALLNVFVWIGYLSSAVNPLVYTLFNKTYRSAFSRYIQ-CQYKENKKPLQLILVNTIPALAYKSSQLQMGQKKNSKQDAKTTDNDCSMVALGKQHSEEASKDNSDGVNEKVSCV--------------------------------------------------------------------------------

5H2B_HUMA ------------------------------------------MALSYRVSELQSTIPEHILQSTFVHVISSNWSGLQTESIPEEMKQIVEEQGNKLHWAALLILMVIIPTIGGNTLVILAVSLEKKLQY-ATNYFLMSLAVADLLVGLFVMPIALLTIMFEAMWPLPLVLCPAWLFLDVLFSTASIMHLCAISVDRYIAIKKPIQANQYNSR---ATAFIKITVVWLISIGIAIPVPIKGI-------------------------ETDVDNPNNITCVLTKERFGDFMLFGSLAAFFTPLAIMIVTYFLTIHALQKKAYLVKNKPPQRLTWLTVSTVFQRDETPC--------------------------------------------------------------------------------------------------SSPEKVAMLDGSRKDKALPNSGDETLMRRTSTIGKKSVQTISNEQRASKVLGIVFFLFLLMWCPFFITNITLVLCDS-CN------QTTLQMLLEIFVWIGYVSSGVNPLVYTLFNKTFRDAFGRYIT-CNYRATKSVKTLRKRSSKIYFRNPMAENSKFFKKHGIRNGINPAMYQSPMRLRSSTIQSSSIILLDTLLLTENEGDKTEEQVSYV----------------------------------------------------------------------

5H2C_HUMA -------------------------------------------MVNLRNAVHSFLVHLIGLLVWQCDISVSPVAAIVTDIFNTSDGGRFKFPDGVQNWPALSIVIIIIMTIGGNILVIMAVSMEKKLHN-ATNYFLMSLAIADMLVGLLVMPLSLLAILYDYVWPLPRYLCPVWISLDVLFSTASIMHLCAISLDRYVAIRNPIEHSRFNSR---TKAIMKIAIVWAISIGVSVPIPVIGLRDE------------------------EKVFVNNTTCVL-NDP--NFVLIGSFVAFFIPLTIMVITYCLTIYVLRRQALMLLHGHTEEPPGLSLDF------------------------------------------------------------------------------------------------------------LKCCKRNTAEEENSANPNQDQNARRRKKKERRPRGTMQAINNERKASKVLGIVFFVFLIMWCPFFITNILSVLCEKSCN------QKLMEKLLNVFVWIGYVCSGINPLVYTLFNKIYRRAFSNYLR-CNYKVEKKP-PVRQIPRVAATALSGRELNVNIYRHTNEPVIEKASDNEPGIEMQVENLELPVNPSSVVSERISSV--------------------------------------------------------------------------------

5H6_HUMAN ----------------------------------------------------------------------MVPEPGPTANSTPAWGAGPPSAPGGSGWVAAALCVVIALTAAANSLLIALICTQPALRN-TSNFFLVSLFTSDLMVGLVVMPPAMLNALYG-RWVLARGLCLLWTAFDVMCCSASILNLCLISLDRYLLILSPLRYKLRMTP---LRALALVLGAWSLAALASFLPLLLGWHELG-----------------------HARPPVPGQCRLLASL--PFVLVASGLTFFLPSGAICFTYCRILLAARKQAVQVASLTTG-----------------------------------------------------------------------------------------------------------------------------MASQASETLQVPRTPRPGVESADSRRLATKHSRKALKASLTLGILLGMFFVTWLPFFVANIVQAVCD-----------CISPGLFDVLTWLGYCNSTMNPIIYPLFMRDFKRALGRFLP-CPRCPRERQASLASPSLRTSHSGPRPGLSLQQVLPLPLPPDSDSDSDAGSGGSSGLRLTAQLLLPGEATQDPPLPTRAAAAVNFFNIDPAEPELRPHPLGIPTN---------------------------------------------------

A1AA_HUMA ----------------------------------------------------------------------MVFLSGNASDSSNCTQPPAPVNISKAILLGVILGGLILFGVLGNILVILSVACHRHLHS-VTHYYIVNLAVADLLLTSTVLPFSAIFEVLG-YWAFGRVFCNIWAAVDVLCCTASIMGLCIISIDRYIGVSYPLRYPTIVTQ---RRGLMALLCVWALSLVISIGPLFG-WRQPA--------------------------PEDETICQINEEP--GYVLFSALGSFYLPLAIILVMYCRVYVVAKRESRGLKSGLKTD---------------------------------------------------------------------------------------------------------------------KSDSEQVTLRIHRKNAPAGGSGMASAKTKTHFSVRLLKFSREKKAAKTLGIVVGCFVLCWLPFFLVMPIGSFFP-DFK--------PSETVFKIVFWLGYLNSCINPIIYPCSSQEFKKAFQNVLRIQCLCRKQSSKHALGYTLHPPSQAVEGQHKDMVRIPVGSRETFYRISKTDGVCEWKFFSSMPRGSARITVSKDQSSCTTARVRSKSFLQVCCCVGPSTPSLDKNHQVPTIKVHTISLSENGEEV--------------------------------

A1AB_HUMA ---------------------------------------------------MNPDLDTGHNTSAPAHWGELKNANFTGPNQTSSNSTLPQLDITRAISVGLVLGAFILFAIVGNILVILSVACNRHLRT-PTNYFIVNLAMADLLLSFTVLPFSAALEVLG-YWVLGRIFCDIWAAVDVLCCTASILSLCAISIDRYIGVRYSLQYPTLVTR---RKAILALLSVWVLSTVISIGPLLG-WKEPA--------------------------PNDDKECGVTEEP--FYALFSSLGSFYIPLAVILVMYCRVYIVAKRTTKNLEAGVMKEMSN------------------------------------------------------------------------------------------------------------------SKELTLRIHSKNFHEDTLSSTKAKGHNPRSSIAVKLFKFSREKKAAKTLGIVVGMFILCWLPFFIALPLGSLFS-TLK--------PPDAVFKVVFWLGYFNSCLNPIIYPCSSKEFKRAFVRILG-CQCRGRRRRRRRRRRLGGCAYTYRPWTRGGSLERSQSRKDSLDDSGSCLSGSQRTLPSASPSPGYLGRGAPPPVELCAFPEWKAPGALLSLPAPEPPGRRGRHDSGPLFTFKLLTEPESPGTDGGASNGGCEAAADVANGQPGFKSNMPLAPGQF

A1AD_HUMA MTFRDLLSVSFEGPRPDSSAGGSSAGGGGGSAGGAAPSEGPAVGGVPGGAGGGGGVVGAGSGEDNRSSAGEPGSAGAGGDVNGTAAVGGLVVSAQGVGVGVFLAAFILMAVAGNLLVILSVACNRHLQT-VTNYFIVNLAVADLLLSATVLPFSATMEVLG-FWAFGRAFCDVWAAVDVLCCTASILSLCTISVDRYVGVRHSLKYPAIMTE---RKAAAILALLWVVALVVSVGPLLG-WKEP--------------------------VPPDERFCGITEEA--GYAVFSSVCSFYLPMAVIVVMYCRVYVVARSTTRSLEAGVKRERGKASEV---------------------------------------------------------------------------------------------------------------VLRIHCRGAATGADGAHGMRSAKGHTFRSSLSVRLLKFSREKKAAKTLAIVVGVFVLCWFPFFFVLPLGSLFP-QLK--------PSEGVFKVIFWLGYFNSCVNPLIYPCSSREFKRAFLRLLR-CQCRRRRRRRPLWRVYGHHWRASTSGLRQDCAPSSGDAPPGAPLALTALPDPDPEPPGTPEMQAPVASRRKPPSAFREWRLLGPFRRPTTQLRAKVSSLSHKIRAGGAQRAEAACAQRSEVEAVSLGVPHEVAEGATCQAYELADYSNLRETDI-

A2AA_HUMA ---------------------------------------------------------------MGSLQPDAGNASWNGTEAPGGGARATPYSLQVTLTLVCLAGLLMLLTVFGNVLVIIAVFTSRALKA-PQNLFLVSLASADILVATLVIPFSLANEVMG-YWYFGKAWCEIYLALDVLFCTSSIVHLCAISLDRYWSITQAIEYNLKRTP---RRIKAIIITVWVISAVISFPPLISIEKKGG----------------------GGGPQPAEPRCEINDQK--WYVISSCIGSFFAPCLIMILVYVRIYQIAKRRTRVPPSRRGPDAVAAPPGGTERRPNGLGPERSAGPGGAEAEPLPTQLNGAPGEPAPAGPRDTDALDLEESSSSDHAERPPGPRRPERGPRGKGKARAS---------------------------QVKPGDSLPRRGPGATGIGTPAAGPGEERVGAAKASRWRGRQNREKRFTFVLAVVIGVFVVCWFPFFFTYTLTAV---GCS--------VPRTLFKFFFWFGYCNSSLNPVIYTIFNHDFRRAFKKILCRGDRKRIV----------------------------------------------------------------------------------------------------------------------------------------------------

A2AB_HUMA ------------------------------------------------------------------------------------MDHQDPYSVQATAAIAAAITFLILFTIFGNALVILAVLTSRSLRA-PQNLFLVSLAAADILVATLIIPFSLANELLG-YWYFRRTWCEVYLALDVLFCTSSIVHLCAISLDRYWAVSRALEYNSKRTP---RRIKCIILTVWLIAAVISLPPLIYKGDQG-------------------------PQPRGRPQCKLNQEA--WYILASSIGSFFAPCLIMILVYLRIYLIAKRSNRRGPRAKGGPGQGESKQPRPDHGGALASAKLPALASVASAREVNGHSKSTGEKEEGETPEDTGTRALPPSWAALPNSGQGQKEGVCGASPEDEAEEEEEEEEEEEECEPQAVPVSPAS------ACSPPLQQPQGSRVLATLRGQVLLGRGVGAIGGQWWRRRAQLTREKRFTFVLAVVIGVFVLCWFPFFFSYSLGAICPKHCK--------VPHGLFQFFFWIGYCNSSLNPVIYTIFNQDFRRAFRRILCRPWTQTAW----------------------------------------------------------------------------------------------------------------------------------------------------

A2AC_HUMA ---------------------------------------------MASPALAAALAVAAAAGPNASGAGERGSGGVANASGASWGPPRGQYSAGAVAGLAAVVGFLIVFTVVGNVLVVIAVLTSRALRA-PQNLFLVSLASADILVATLVMPFSLANELMA-YWYFGQVWCGVYLALDVLFCTSSIVHLCAISLDRYWSVTQAVEYNLKRTP---RRVKATIVAVWLISAVISFPPLVSLYRQPD--------------------------GAAYPQCGLNDET--WYILSSCIGSFFAPCLIMGLVYARIYRVAKLRTRTLSEKRAPVGPDGASPTTENGLGAAAGAGENGHCAPPPADVEPDESSAAAERRRRRGALRRGGRRRAGAEGGAGGADGQGAGPGAAESGAL---------------------------------TASRSPGPGGRLSRASSRSVEFFLSRRRRARSSVCRRKVAQAREKRFTFVLAVVMGVFVLCWFPFFFSYSLYGICREACQ--------VPGPLFKFFFWIGYCNSSLNPVIYTVFNQDFRRSFKHILFRRRRRGFRQ---------------------------------------------------------------------------------------------------------------------------------------------------

B1AR_HUMA --------------------------------------MGAGVLVLGASEPGNLSSAAPLPDGAATAARLLVPASPPASLLPPASESPEPLSQQWTAGMGLLMALIVLLIVAGNVLVIVAIAKTPRLQT-LTNLFIMSLASADLVMGLLVVPFGATIVVWG-RWEYGSFFCELWTSVDVLCVTASIETLCVIALDRYLAITSPFRYQSLLTR---ARARGLVCTVWAISALVSFLPILMHWWRAE-------------------SDEARRCYNDPKCCDFVTNR--AYAIASSVVSFYVPLCIMAFVYLRVFREAQKQVKKIDSCERRFLGGPARPPS---------------------------------------------------------------------------------------------------------PSPSPVPAPAPPPGPPRPAAAAATAPLANGRAGKRRPSRLVALREQKALKTLGIIMGVFTLCWLPFFLANVVKAFHRE----------LVPDRLFVFFNWLGYANSAFNPIIYCR-SPDFRKAFQGLLC-CARRAARRRHATHGDRPRASGCLARPG-----PPPSPGAASDDDDDDVVGATPPARLLEPWAGCNGGAAADSDSSLDEPCRPGFASESKV-----------------------------------------------------------------

B2AR_HUMA ---------------------------------------------------------------MGQPGNGSAFLLAPNRSHAPDHDVTQQRDEVWVVGMGIVMSLIVLAIVFGNVLVITAIAKFERLQT-VTNYFITSLACADLVMGLAVVPFGAAHILMK-MWTFGNFWCEFWTSIDVLCVTASIETLCVIAVDRYFAITSPFKYQSLLTK---NKARVIILMVWIVSGLTSFLPIQMHWYRAT-------------------HQEAINCYANETCCDFFTNQ--AYAIASSIVSFYVPLVIMVFVYSRVFQEAKRQLQKIDKSEGRF-----------------------------------------------------------------------------------------------------------------------------------HVQNLSQVEQDGRTGHGLRRSSKFCLKEHKALKTLGIIMGTFTLCWLPFFIVNIVHVIQD----------NLIRKEVYILLNWIGYVNSGFNPLIYCR-SPDFRIAFQELLCLRRSSLKAYGNGYSSNGNTGEQSGYHVEQEKENKLLCEDLPGTEDFVGHQGTVPSDNIDSQGRNCSTNDSLL------------------------------------------------------------------------------------

B3AR_HUMA -----------------------------------------------------------MAPWPHENSSLAPWPDLPTLAPNTANTSGLPGVPWEAALAGALLALAVLATVGGNLLVIVAIAWTPRLQT-MTNVFVTSLAAADLVMGLLVVPPAATLALTG-HWPLGATGCELWTSVDVLCVTASIETLCALAVDRYLAVTNPLRYGALVTK---RCARTAVVLVWVVSAAVSFAPIMSQWWRVG------------------ADAEAQRCHSNPRCCAFASNM--PYVLLSSSVSFYLPLLVMLFVYARVFVVATRQLRLLRGELGRFPPEES---------------------------------------------------------------------------------------------------------------------PPAPSRSLAPAPVGTCAPPEGVPACGRRPARLLPLREHRALCTLGLIMGTFTLCWLPFFLANVLRALGGP---------SLVPGPAFLALNWLGYANSAFNPLIYCR-SPDFRSAFRRLLCRCGRRLPPEPCAAARPALFPSGVPA-----------------------ARSSPAQPRLCQRLDGASWGVS--------------------------------------------------------------------------------------

D2DR_HUMA --------------------------------------------------------------MDPLNLSWYDDDLERQNWSRPFNGSDGKADRPHYNYYATLLTLLIAVIVFGNVLVCMAVSREKALQT-TTNYLIVSLAVADLLVATLVMPWVVYLEVVG-EWKFSRIHCDIFVTLDVMMCTASILNLCAISIDRYTAVAMPMLYNTRYS--SKRRVTVMISIVWVLSFTISCPL-LFGLNNA-----------------------------DQNECII-ANP--AFVVYSSIVSFYVPFIVTLLVYIKIYIVLRRRRKRVNTKRSSRAFRAHLRAPLKGNCTHPEDMKLCTVIMKSNGSFPVNRRRVEAARRAQELEMEMLSSTSPPERTRYSPIPPSHHQLTLPDPSHHGLHSTPDSPA---------------------KPEKNGHAKDHPKIAKIFEIQTMPNGKTRTSLKTMSRRKLSQQKEKKATQMLAIVLGVFIICWLPFFITHILNIHCD----------CNIPPVLYSAFTWLGYVNSAVNPIIYTTFNIEFRKAFLKILH-C----------------------------------------------------------------------------------------------------------------------------------------------------------

D3DR_HUMA -------------------------------------------------------------------MASLSQLSSHLNYTCGAENSTGASQARPHAYYALSYCALILAIVFGNGLVCMAVLKERALQT-TTNYLVVSLAVADLLVATLVMPWVVYLEVTGGVWNFSRICCDVFVTLDVMMCTASILNLCAISIDRYTAVVMPVHYQHGTGQSSCRRVALMITAVWVLAFAVSC-PLLFGFNTTG----------------------------DPTVCSI-SNP--DFVIYSSVVSFYLPFGVTVLVYARIYVVLKQRRRKRILTRQNSQCNSVRPGFPQQTLSPDPAHLELKRYYSICQDTALGGPGFQERGGELKREEK----------------------------------------------------------------TRNSLSPTIAPKLSLEVRKLSNGRLSTSLKLGPLQPRGVPLREKKATQMVAIVLGAFIVCWLPFFLTHVLNTHCQT---------CHVSPELYSATTWLGYVNSALNPVIYTTFNIEFRKAFLKILS-C----------------------------------------------------------------------------------------------------------------------------------------------------------

D4DR_HUMA --------------------------------------------------------------MGNRSTADADGLLAGRGPAAGASAGASAGLAGQGAAALVGGVLLIGAVLAGNSLVCVSVATERALQT-PTNSFIVSLAAADLLLALLVLPLFVYSEVQGGAWLLSPRLCDALMAMDVMLCTASIFNLCAISVDRFVAVAVPLRYNRQGGS---RRQLLLIGATWLLSAAVAA-PVLCGLNDV--------------------------RGRDPAVCRL-EDR--DYVVYSSVCSFFLPCPLMLLLYWATFRGLQRWEVARRAKLHGRAPRRPSGPGPPSPTPPAPRLPQDPCGPDCAPPAPGLPRGPCGPDCAPAAPGLPPDPCGPDCAPPAPGLPQDPCGPDCAPPAPGLPRGPCGPDCAPPAPGLPQDPCGPDCAP---PAPGLPPDPCGSNCAPPDAVRAAALPPQTPPQTRRRRRAKITGRERKAMRVLPVVVGAFLLCWTPFFVVHITQALCPA---------CSVPPRLVSAVTWLGYVNSALNPVIYTVFNAEFRNVFRKALRACC---------------------------------------------------------------------------------------------------------------------------------------------------------

DADR_HUMA -------------------------------------------------------------------------MRTLNTSAMDGTGLVVERDFSVRILTACFLSLLILSTLLGNTLVCAAVIRFRHLRSKVTNFFVISLAVSDLLVAVLVMPWKAVAEIAG-FWPFGS-FCNIWVAFDIMCSTASILNLCVISVDRYWAISSPFRYERKMTP---KAAFILISVAWTLSVLISFIPVQLSWHKAK--------------PTSPSDGNATSLAETIDNCDSSLSR--TYAISSSVISFYIPVAIMIVTYTRIYRIAQKQIRRIAALER-------------------------------------------------------------------------------------------------------------------------------AAVHAKNCQTTTGNGKPVECSQPESSFKMSFKRETKVLKTLSVIMGVFVCCWLPFFILNCILPFCGSGET----QPFCIDSNTFDVFVWFGWANSSLNPIIYA-FNADFRKAFSTLLG-CYRLCPATNNAIETVSINNNGAAMFSSHHEPRGSISKECNLVYLIPHAVGSSEDLKKEEAAGIARPLEKLSPALSV-----ILDYDTDVSLEKIQPITQNGQHPT--------------------------------------------------

DBDR_HUMA --------------------------------------------------------MLPPGSNGTAYPGQFALYQQLAQGNAVGGSAGAPPLGPSQVVTACLLTLLIIWTLLGNVLVCAAIVRSRHLRANMTNVFIVSLAVSDLFVALLVMPWKAVAEVAG-YWPFG-AFCDVWVAFDIMCSTASILNLCVISVDRYWAISRPFRYKRKMTQ---RMALVMVGLAWTLSILISFIPVQLNWHRDQAASWGGLDLPNNLANWTPWEEDFWEPDVNAENCDSSLNR--TYAISSSLISFYIPVAIMIVTYTRIYRIAQVQIRRISSLER--------------------------------------------------------------------------------------------------------------------------------------AAEHAQSCRSSAACAPDTSLRASIKKETKVLKTLSVIMGVFVCCWLPFFILNCMVPFCSGHPEGPPAGFPCVSETTFDVFVWFGWANSSLNPVIYA-FNADFQKVFAQLLG-CSHFCSRTPVETVNISNELISYNQDIVFHKEIAAAYIHMMPNAVTPGNREVDNDEEEGPFDRMFQIYQTSPDGDPVAESVWELDCEGEISLDKITPFTPNGFH----------------------------------------------------

GPR102 -----------------------------------------------------------------MTSNFSQPVVQLCYEDVNGSCIETPYSPGSRVILYTAFSFGSLLAVFGNLLVMTSVLHFKQLHS-PTNFLIASLACADFLVGVTVMLFSMVRTVES-CWYFGAKFCTLHSCCDVAFCYSSVLHLCFICIDRYIVVTDPLVYATKFTV---SVSGICISVSWILPLTYSGAVFYTGVNDDG-------------------LEELVSALNCVGGCQIIVSQ--GWVLIDFLL-FFIPTLVMIILYSKIFLIAKQQAIKIETTSS------------------------------------------------------------------------------------------------------------------------------------------------KVESSSESYKIRVAKRERKAAKTLGVTVLAFVISWLPYTVDILIDAFMG----------FLTPAYIYEICCWSAYYNSAMNPLIYALFYPWFRKAIKLILSGDVLKASSSTISLFLE--------------------------------------------------------------------------------------------------------------------------------------------

GPR57 -----------------------------------------------------------------MDLTYIPEDLSSCPKFVNKILSSHQPLFSCPGDNVFGYDWSHDYPLFGNLVIMVSISHFKQLHS-PTNFLILSMATTDFLLGFVIMPYSIMRSVES-CWYFGDGFCKFHTSFDMMLRLTSIFHLCSIAIDRFYAVCYPLHYTTKMTN---STIKQLLAFCWSVPALFSFGLVLSEADVSG-------------------MQSYKILVACFNFCALTFNK--FWGTILFTTCFFTPGSIMVGIYGKIFIVSKQHARVISHVP--------------------------------------------------------------------------------------------------------------------------------------------------ENTKGAVKKHLSKKKDRKAAKTLGIVMGVFLACWLPCFLAVLIDPYLD----------YSTPILILDLLVWLRYFNSTCNPLIHGFFNPWFQKAFKYIVSGKIFSSHSETANLFPEAH------------------------------------------------------------------------------------------------------------------------------------------

GPR58 --------------------------------------------------------------------------------------------------MYSFMAGSIFITIFGNLAMIISISYFKQLHT-PTNFLILSMAITDFLLGFTIMPYSMIRSVEN-CWYFGLTFCKIYYSFDLMLSITSIFHLCSVAIDRFYAICYPLLYSTKITI---PVIKRLLLLCWSVPGAFAFGAVFSEAYADG-------------------IEGYDILVACSSSCPVMFNK--LWGTTLFMAGFFTPGSMMVGIYGKIFAVSRKHAHAINN--------------------------------------------------------------------------------------------------------------------------------------------------------LRENQNNQVKKDKKAAKTLGIVIGVFLLCWFPCFFTILLDPFLN----------FSTPVVLFDALTWFGYFNSTCNPLIYGFFYPWFRRALKYILLGKIFSSCFHNTILCMQKESE----------------------------------------------------------------------------------------------------------------------------------------

HH2R_HUMA -----------------------------------------------------------------------------MAPNGTASSFCLDSTACKI-TITVVLAVLILITVAGNVVVCLAVGLNRRLRN-LTNCFIVSLAITDLLLGLLVLPFSAIYQLSC-KWSFGKVFCNIYTSLDVMLCTASILNLFMISLDRYCAVMDPLRYPVLVT---PVRVAISLVLIWVISITLSFLSIHLGWNSRN---------------------ETSKGNHTTSKCKVQVNE--VYGLVDGLVTFYLPLLIMCITYYRIFKVARDQAKRINH---------------------------------------------------------------------------------------------------------------------------------------------------------ISSWKAATIREHKATVTLAAVMGAFIICWFPYFTAFVYRGLRGD---------DAINEVLEAIVLWLGYANSALNPILYAALNRDFRTGYQQLFC-CRLANRNSHKTSLRSNASQLSRTQSREPRQQEEKPLKLQVWSGTEVTAPQGATDR----------------------------------------------------------------------------------------------------

PNR_HUMAN --------------------------------------------------------------MRAVFIQGAEEHPAAFCYQVNGSCPRTVHTLGIQLVIYLTCAAGMLIIVLGNVFVAFAVSYFKALHT-PTNFLLLSLALADMFLGLLVLPLSTIRSVES-CWFFGDFLCRLHTYLDTLFCLTSIFHLCFISIDRHCAICDPLLYPSKFTV---RVALRYILAGWGVPAAYTSLFLYTDVVETR-------------------LSQWLEEMPCVGSCQLLLNK--FWGWLN-FPLFFVPCLIMISLYVKIFVVATRQAQQITT--------------------------------------------------------------------------------------------------------------------------------------------------------LSKSLAGAAKHERKAAKTLGIVVGIYLLCWLPFTIDTMVDSLLH----------FITPPLVFDIFIWFAYFNSACNPIIYVFSYQWFRKALKLTLSQKVFSPQTRTVDLYQE--------------------------------------------------------------------------------------------------------------------------------------------

Group A18a

ACM1_HUMA -------------------------------------------MNTSAPPAVSPNITVLAPGK---GPWQVAFIGITTGLLSLATVTGNLLVLISFKVNTELKTVNNYFLLSLACADLIIGTFSMNLYTTYLLMGHWALGTLACDLWLALDYVASNASVMNLLLISFDRYFSVTRPLSYRAKRTPRRAALMIGLAWLVSFVLWAPAILFWQYLVGERTVL-AGQCYIQFLSQPIITFGTAMAAFYLPVTVMCTLYWRIYRETENRARELAALQGSETPGKGGGSSSSS----------------------------------------------------------------------------------------------ERSQPGAEGSPETPPGRCCRCCRAPRLLQAYSWKEEEEEDEGSMESLTSSEGEEPGSEVVIKMPMVDPEAQAPTKQPPRSSPNTVKRPTKKGRDRAGKGQKPRGKEQLAKRKTFSLVKEKKAARTLSAILLAFILTWTPYNIMVLVSTFCKDCVPETLWELGYWLCYVNSTINPMCYALCNKAFRDTFRLLLLCRWDKRRWRKIPKRPGSVHRTPSRQC----

ACM2_HUMA ---------------------------------------------MNNSTNSSNNSLALTSPY---KTFEVVFIVLVAGSLSLVTIIGNILVMVSIKVNRHLQTVNNYFLFSLACADLIIGVFSMNLYTLYTVIGYWPLGPVVCDLWLALDYVVSNASVMNLLIISFDRYFCVTKPLTYPVKRTTKMAGMMIAAAWVLSFILWAPAILFWQFIVGVRTVE-DGECYIQFFSNAAVTFGTAIAAFYLPVIIMTVLYWHISRASKSRIKKDKKEPVANQDPVSPSLVQGRIVKPNNNNMPSSDDGLEHNKIQNGKAP---------------------------------------------------------------------RDPVTENCVQGEEKESSNDSTSVSAVASNMRDDEITQDENTVSTSLGHSKDENSKQTCIRIGTKTPKSDSCTPTNTTVEVVGSSGQNGDEKQNIVARKIVKMTKQPA-KKKPPPSREKKVTRTILAILLAFIITWAPYNVMVLINTFCAPCIPNTVWTIGYWLCYINSTINPACYALCNATFKKTFKHLLMCHYKNIGATR--------------------

ACM3_HUMA MTLHNNSTTSPLFPNISSSWIHSPSDAGLPPGTVTHFGSYNVSRAAGNFSSPDGTTDDPLGGH---TVWQVVFIAFLTGILALVTIIGNILVIVSFKVNKQLKTVNNYFLLSLACADLIIGVISMNLFTTYIIMNRWALGNLACDLWLAIDYVASNASVMNLLVISFDRYFSITRPLTYRAKRTTKRAGVMIGLAWVISFVLWAPAILFWQYFVGKRTVP-PGECFIQFLSEPTITFGTAIAAFYMPVTIMTILYWRIYKETEKRTKELAGLQASGTEAETENFVHPTGSSRSCSSYELQQQSMKRSNRRKYGRCHFWFTTKSWKPSSEQMDQDHSSSDSWNNNDAAASLENSASSDEEDIGSETRAIYSIVLKLPGHSTILNSTKLPSSDNLQVPEEELGMVD-----LERKADKLQAQKSVDDGGSFPKSFSKLPIQLESAVDTAKTSDVNSSVGKSTATL------PLSFKEATLAKRFALKTRSQITKRKRMSLVKEKKAAQTLSAILLAFIITWTPYNIMVLVNTFCDSCIPKTFWNLGYWLCYINSTVNPVCYALCNKTFRTTFKMLLLCQCDKKKRRKQQYQQRQSVIFHKRAPEQAL

ACM4_HUMA ------------------------------------MANFTPVNGSSGNQSVRLVTSSSHNRY---ETVEMVFIATVTGSLSLVTVVGNILVMLSIKVNRQLQTVNNYFLFSLACADLIIGAFSMNLYTVYIIKGYWPLGAVVCDLWLALDYVVSNASVMNLLIISFDRYFCVTKPLTYPARRTTKMAGLMIAAAWVLSFVLWAPAILFWQFVVGKRTVP-DNQCFIQFLSNPAVTFGTAIAAFYLPVVIMTVLYIHISLASRSRVHKHRPEGPKEKKAKTLAFLKSPLMKQSVKKPPPGEAAREELRNGKLEEAPPPALPPPP------------------------------------------------------------RPVADKDTSNESSSGSATQNTKERPATELSTTEATTPAMPAPPLQPRALNPASRWSKIQIVT----KQTGNECVTAIEIVPAT--PAGMRPAANVARKFASIARNQVRKKRQMAARERKVTRTIFAILLAFILTWTPYNVMVLVNTFCQSCIPDTVWSIGYWLCYVNSTINPACYALCNATFKKTFRHLLLCQYRNIGTAR--------------------

ACM5_HUMA --------------------------------------MEGDSYHNATTVNGTPVNHQPLERH---RLWEVITIAAVTAVVSLITIVGNVLVMISFKVNSQLKTVNNYYLLSLACADLIIGIFSMNLYTTYILMGRWALGSLACDLWLALDYVASNASVMNLLVISFDRYFSITRPLTYRAKRTPKRAGIMIGLAWLISFILWAPAILCWQYLVGKRTVP-LDECQIQFLSEPTITFGTAIAAFYIPVSVMTILYCRIYRETEKRTKDLADLQGSDSVTKAEKRKPAHRALFRSCLRCPRPTLAQRERNQASWSSSRRSTSTTGKPSQATGPSANWAKAEQLTTCSSYPSSEDEDKPATD----------------------PVLQVVYKSQGKESPGEEFSAEETEETFVKAETEKSDYDTPNYLLSPAAAHRPKSQKCVAYKFRLVVKADGNQETNNGCHKVKIMPCPFPVAKEPSTKGLNPNPSHQMTKRKRVVLVKERKAAQTLSAILLAFIITWTPYNIMVLVSTFCDKCVPVTLWHLGYWLCYVNSTVNPICYALCNRTFRKTFKMLLLCRWKKKKVEEKLYWQGNSKLP---------

HH1R_HUMA --------------------------------------------MSLPNSSCLLEDKMCEGNKTTMASPQLMPLVVVLSTICLVTVGLNLLVLYAVRSERKLHTVGNLYIVSLSVADLIVGAVVMPMNILYLLMSKWSLGRPLCLFWLSMDYVASTASIFSVFILCIDRYRSVQQPLRYLKYRTKTRASATILGAWFLSF-LWVIPILGWNHFMQQTSVRREDKCETDFYDVTWFKVMTAIINFYLPTLLMLWFYAKIYKAVRQHCQHRELINRSLPSFSEIKLRPENPKGDAKKPGKESPWEVLKRKPKDAGGGSVLKSPSQTPKEMKSPVVFSQEDDREVDKLYCFPL-------------------------------------------DIVHMQAAAEGSSRDYVAVNRSHGQLKTDEQGLNTHGASEISEDQMLGDSQSFSRTDSDTTTETAPGKGKLRSGSNTGLDYIKFTWKRLRSHSRQYVSG---LHMNRERKAAKQLGFIMAAFILCWIPYFIFFMVIAFCKNCCNEHLHMFTIWLGYINSTLNPLIYPLCNENFKKTFKRILHIRS---------------------------

Group A18b

AA1R_HUMA ---MPPSISAFQAAYIGIEVLIALVSVPGNVLVIWAVKVNQALRDATFCFIVSLAVADVAVGALVIPLAILINIGPQTYFHTCLMVACPVLILTQSSILALLAIAVDRYLRVKIPLRYKMVVTPRRAAVAIAGCWILSFVVGLTPMFGWNNLSAVER----AWAANGSMGEPVIKCEFEKVISMEYMVYFNFFVWVLPPLLLMVLIYLEVFYLIRKQLNKKVSAS--SGDPQKYYGKELKIAKSLALILFLFALSWLPLHILNCITLFCP-SC-HKPSILTYIAIFLTHGNSAMNPIVYAFRIQKFRVTFLKIWNDHFRCQPAP-PIDEDL---------------------------------PEERPDD----------------------------------------------------

AA2A_HUMA ------MPIMGSSVYITVELAIAVLAILGNVLVCWAVWLNSNLQNVTNYFVVSLAAADIAVGVLAIPFAITISTGFCAACHGCLFIACFVLVLTQSSIFSLLAIAIDRYIAIRIPLRYNGLVTGTRAKGIIAICWVLSFAIGLTPMLGWNNCGQPKE----GKNHSQGCGEGQVACLFEDVVPMNYMVYFNFFACVLVPLLLMLGVYLRIFLAARRQLKQMESQPLPGERARSTLQKEVHAAKSLAIIVGLFALCWLPLHIINCFTFFCP-DCSHAPLWLMYLAIVLSHTNSVVNPFIYAYRIREFRQTFRKIIRSHVLRQQEPFKAAGTSARVLAAHGSDGEQVSLRLNGHPPGVWANGSAPHPERRPNGYALGLVSGGSAQESQGNTGLPDVELLSHELKGVCPEPPGLDDPLAQDGAGVS

AA2B_HUMA -----MLLETQDALYVALELVIAALSVAGNVLVCAAVGTANTLQTPTNYFLVSLAAADVAVGLFAIPFAITISLGFCTDFYGCLFLACFVLVLTQSSIFSLLAVAVDRYLAICVPLRYKSLVTGTRARGVIAVLWVLAFGIGLTPFLGWNSKDSATNNCTEPWDGTTNESCCLVKCLFENVVPMSYMVYFNFFGCVLPPLLIMLVIYIKIFLVACRQLQRTELM----DHSRTTLQREIHAAKSLAMIVGIFALCWLPVHAVNCVTLFQPAQGKNKPKWAMNMAILLSHANSVVNPIVYAYRNRDFRYTFHKIISRYLLCQAD-VKSGNGQAGVQPALGVGL---------------------------------------------------------------------------------

AA3R_HUMA MPNNSTALSLANVTYITMEIFIGLCAIVGNVLVICVVKLNPSLQTTTFYFIVSLALADIAVGVLVMPLAIVVSLGITIHFYSCLFMTCLLLIFTHASIMSLLAIAVDRYLRVKLTVRYKRVTTHRRIWLALGLCWLVSFLVGLTPMFGWNMKLTSEY----------HRNVTFLSCQFVSVMRMDYMVYFSFLTWIFIPLVVMCAIYLDIFYIIRNKLSLNLS-N--SKETGAFYGREFKTAKSLFLVLFLFALSWLPLSIINCIIYF---NG-EVPQLVLYMGILLSHANSMMNPIVYAYKIKKFKETYLLILKACVVCHPSD-SLDTSIEKNSE---------------------------------------------------------------------------------------

Group A18c

GP27_HUMA MANAS----EPGGSGGGEAAALGLKLATLSLLLCVSLAGNVLFALLIVRERSLHRAPYYLLLDLCLADGLRALACLPAVMLAARRAAAAAGAPPGALGCKLLAFLAALFCFHAAFLLLGVGVTRYLAIAHHRFYAERLAGWPCAAMLVCAAWALALAAAFPPVLDGGGDD---EDAPCALEQRPDGAPGALGFLLLLAVVVGATHLVYLRLLFFIHDRRKMRPARLVPAVSHDWTFHGPGATGQAAANWTAGFGRGPTPPALVGIRPAGPGRGARRLLVLEEFKTEKRLCKMFYAVTLLFLLLWGPYVVASYLRVLVRPGAVPQAYLTASVWLTFAQAGINPVVCFLFNRELRDCFRA--QFPCCQSPRTTQATHPCDLKGIGL

GP85_HUMA MANYSHAADNILQNLSP--LTAFLKLTSLGFIIGVSVVGNLLISILLVKDKTLHRAPYYFLLDLCCSDILRSAICFPFVFNSVKNGSTWTY---GTLTCKVIAFLGVLSCFHTAFMLFCISVTRYLAIAHHRFYTKRLTFWTCLAV-ICMVWTLSVAMAFPPVLDVGTYSFIREEDQCTFQHRSFRANDSLGFMLLLALILLATQLVYLKLIFFVHDRRKMKPVQFVAAVSQNWTFHGPGASGQAAANWLAGFGRGPTPPTLLGIRQNANTTGRRRLLVLDEFKMEKRISRMFYIMTFLFLTLWGPYLVACYWRVFARGPVVPGGFLTAAVWMSFAQAGINPFVCIFSNRELRRCFST--TLLYCRKSR--LPREPYCVI----

SREB3 MANTTGEPEEVSGALSPPSASAYVKLVLLGLIMCVSLAGNAILSLLVLKERALHKAPYYFLLDLCLADGIRSAVCFPFVLASVRHGSSWTF---SALSCKIVAFMAVLFCFHAAFMLFCISVTRYMAIAHHRFYAKRMTLWTCAAV-ICMAWTLSVAMAFPPVFDVGTYKFIREEDQCIFEHRYFKANDTLGFMLMLAVLMAATHAVYGKLLLFEYRHRKMKPVQMVPAISQNWTFHGPGATGQAAANWIAGFGRGPMPPTLLGIRQNGHAAS-RRLLGMDEVKGEKQLGRMFYAITLLFLLLWSPYIVACYWRVFVKACAVPHRYLATAVWMSFAQAAVNPIVCFLLNKDLKKCLRTHAPCWGTGGAP--APREPYCVM----

Group A18d

GPR101 MTSTCTNSTRESNSSHTCMPLSKMPISLAHGIIRSTVLVIFLAASFV--GNIVLALVLQRKPQLLQVTNRFIFNLLVTDLLQISLVAPWVVATSVPLFWPLNSHFCTALVSLTHLFAFASVNTIVVVSVDRYLSIIHPLSYPSKMTQRRGYLLLYGTWIVAILQSTPPLYGWGQAAFDERNALCSMIWGASPSYTILSVVSFIVIPLIVMIACYSVVFCAARRQHALLYNVKRHSLEVRVKDCVENEDEEGAEKKEEFQDESEFRRQHEGEVKAKEGRMEAKDGSLKAKEGSTGTSESSVEARGSEEVRESSTVASDGSMEGKEGSTKVEENSMKADKGRTEVNQCSIDLGEDDMEFGEDDINFSEDDVEAVNIPESLPPSRRNSNSNPPLPRCYQCKAAKVIFIIIFSYVLSLGPY-CFLAVLAVWVDVETQVPQWVITIIIWLFFLQCCIHPYVYGYMHKTIKKEIQDML-----------------KKFFCKEKPPKEDSHPDLPGTEGGTEGK----------IVPSYDSATFP--

RE2 MSLNSSLSCRKELSNLTEEEGGE------GGVIITQFIAIIVITIFVCLGNLVIVVTLYKKSYLLTLSNKFVFSLTLSNFLLSVLVLPFVVTSSIRREWIFGVVWCNFSALLYLLISSASMLTLGVIAIDRYYAVLYPMVYPMKITGNRAVMALVYIWLHSLIGCLPPLFGWSSVEFDEFKWMCVAAWHREPGYTAFWQIWCALFPFLVMLVCYGFIFRVAR--------VKARKVHCGTVVIVEEDAQ-------------------------RTGR----------KNSSTSTSSSGSRRNAFQGVVYSAN----------------------------------------------------------------------------------QCKALITILVVLGAFMVTWGPYMVVIASEALWG--KSSVSPSLETWATWLSFASAVCHPLIYGLWNKTVRKELLGMCFGDRYYREPFVQRQRTSRLFSISNRITDLGLSPHLTALMAGGQPLGHSSSTGDTGFSCSQDSGNLRAL

Group A18e

GP61_HUMA MESSPIPQSSGNSSTLGRVPQTPGPSTASGVPEVGLRDVASESVALFFMLLLDLTAVAGNAAVMAVIAKTPALRKFVFVFHLCLVDLLAALTLMPLAMLSSPA-LFDHALFGEVACRLYLFLSVCFVSLAILSVSAINVERYYYVVHPMRYEVRMTLGLVASVLVGVWVKALAMASVPVLGRVSWEEGAPSVPPHCS-LQWSHSAYCQLFVVVFAVLYFLLPLLLILLVYCSMFRVARVAAMPDGPLPTWMETPRQRSESLSSRSTMVTSSGAPQTTPHRTFGGGKAAVVLLAVGGQFLLCWLPYFSFHLYVALSAQPISTGQVE---SVVTWIGYFCFTSNPFFYGCLNRQIRGELSKQFVCFFKPAPEEELRLPSREGSIEENFLQFLQGTGCPSESWVSRPLPSPKQEPPAVDFRIQAR--

GP62_HUMA ----------------------------MANSTGLNASEVAGSLGLILAAVVEVGALLGNGALLVVVLRTPGLRDALYLAHLCVVDLLAAASIMPLGLLAAPPPGLGRVRLGPAPCRAARFLSAALLPACTLGVAALGLARYRLIVHPLRPGSRPPP---VLVLTAVWAAAGLLGALSLLGPPPAPPPAPA---RCSVLAGGLGP----FRPLWALLAFALPALLLLGAYGGIFVVARRAALRPPRPARGSRLRSDSL----------DSRLSILPPLRPRLPGGKAALAPALAVGQFAACWLPYGCACLAPA--------ARAAEAEAAVTWVAYSAFAAHPFLYGLLQRPVRLALGRLSRRALPGPVRACTPQAWHPRALLQCLQRPPEGPAVGPSEAPEQTPELAGGRSPAYQGPPESSLS

Group A18f

GP63_HUMA MVFSAVLTAFHTGTSNTTFVVYENTYMNITLPPPFQHPDLSPLLRYSFETMAPTGLSSLTVNSTAVPTTPAAFKSLNLPLQITLSAIMIFILFVSFLGNLVVCLMVYQKAAMRSAINILLASLAFADMLLAVLNMPFALVTILTTRWIFGKFFCRVSAMFFWLFVIEGVAILLIISIDRFLIIVQRQDKLNPYRAKVLIAVSW----ATSFCVAFPLAVGNPDLQIPSRAPQCVFGYTTNPGYQAYVILISLISFFIPFLVILYSFMGILNTLRHNALRIHSYPEGICLSQASKLGLMSLQRPFQMSIDMGFKTRAFTTILILFAVFIVCWAPFTTYSLVATFSKHFYYQHNFFEISTWLLWLCYLKSALNPLIYYWRIKKFHDACLDMMPKSFKFLPQLPGHTKRRIRPSAVYVCGEHRTVV

PSP24 -----------------------------------------------MACNSTSLEAYTYLLLNTSNASDSGSTQLPAPLRISLAIVMLLMTVVGFLGNTVVCIIVYQRPAMRSAINLLLATLAFSDIMLSLCCMPFTAVTLITVRWHFGDHFCRLSATLYWFFVLEGVAILLIISVDRFLIIVQRQDKLNPRRAKVIIAVSWVLSWVLSFCIAGPSLTGWTLVEVPARAPQCVLGYTELPADRAYVVTLVVAVFFAPFGVMLCAYMCILNTVRKNAVRVHNQSDSLDLRQLTRAGLRRLQRQQQVSVDLSFKTKAFTTILILFVGFSLCWLPHSVYSLLSVFSQRFYCGSSFYATSTCVLWLSYLKSVFNPIVYCWRIKKFREACIELLPQTFQILPKVPERIRRRIQPSTVYCCNENQSAV

Group A18g

GP52_HUMA MNESRWTEWRILNMSSGIVNASERHSCPLGFGHYSVVDVCIFETVVIVLLTFLIIAGNLTVIFAFHCAPLLHHYTTSYFIQTMAYADLFVGVSCLVPTLSLLH-YSTGVHESLTCRVFGYIISVLKSVSMACLACISVDRYLAITKPLSYNQLVTPCRLRICIILIWIYSCLIFLPSFFGWGKPGYHGDIFEWCATSWLTSAYFTGFIVCLLYAPAAFVVCFTYFHIFKICRQHTKEINDRRARFPSHEVDSSRETGHSPDRRYAMVLFRITSVFYMLWLPYIIYFLLESSR--VLDNPTLS-FLTTWLAVSNSFCNCVIYSLSNGVFRLGLRRLFETMCTSCMCVKDQEAQEPKPRKRANSCSI-

GPL_HUMAN ------------MNSTLDGNQSSHPFCLLAFGYLETVNFCLLEVLIIVFLTVLIISGNIIVIFVFHCAPLLNHHTTSYFIQTMAYADLFVGVSCVVPSLSLLHH-PLPVEESLTCQIFGFVVSVLKSVSMASLACISIDRYIAITKPLTYNTLVTPWRLRLCIFLIWLYSTLVFLPSFFHWGKPGYHGDVFQWCAESWHTDSYFTLFIVMMLYAPAALIVCFTYFNIFRICQQHTKDISERQARF-SSQSGETGEVQACPDKRYAMVLFRITSVFYILWLPYIIYFLLESSTGH---SNRFASFLTTWLAISNSFCNCVIYSLSNSVFQRGLKRLSGAMCTSCASQTTANDPYTVRSKGPLNGCHI

Group A18h

GPR84 -----------MWNSSDANFSCYHESVLGYRYVAVSWGVVVAVTGTVGNVLTLLALAIQPKLRTRFNLLIANLTLADLLYCTLLQPFSVDTYL---H-----LHWR-TGATFCRVF-GLLLFASNSVSILTLCLIALGRYLLIAH-PKLFPQVFSAKGIVLALVSTWVVGVASFAPL---WPIYILVPVV-CTCSFDRIRGRP--YTTILMGIYFVLGLSSVGIFYCLIHRQVKRAAQALDQYKLRQASIHSNHVARTDEAMPGRFQELDSRLASGGPSEGISSEPVSAATTQTLEGDSSEVGDQINSKRAKQMAEKSPPEASAKAQPIKGARRAPDSSSEFGKVTRMCFAVFLCFALSYIPFLLLNILDARVQ--APRVVHMLAANLTWLNGCINPVLYAAMNRQFRQAYGSILKRGPRSFHRLH----------------------

GPR88 MTNSSSTSTSTTTGGSLLLLCEEEESWAGRRIPVSLLYSGLAIGGTLANGMVIYLVSSFRKLQTTSNAFIVNGCAADLSVCALWMPQEAVLGLLPSGSAEPPGDWDG-GGGSYRLLRGGLLGLGLTVSLLSHCLVALNRYLLITRAPATYQVLYQRRHTVGMLALSWALALGLVLL-LPPWAPKPGAEPPQ------------VHYPALLAAGALLAQTALLLHCYLGIVRRVRVSVKRVSVLNFHLLHQLPGCAAAAAAFPAAPHAPGPGGAAHPAQPQP-------------------------------------------------LPAALQPRRAQRRLSGLSVLLLCCVFLLATQPLVWVSLASGFS-LPVPWGVQAASWLLCCALSALNPLLYTWRNEEFRRSVRSVLPGVGDAAAAAAAATAVPAMSQAQLGTRAAGQHW

Group A18i

GPR78 MGPGEALLAGLLVMVLAVALLSNALVLLCCAYSAELRTRASGVLLVNLSLGHLLLAALDMPFTLLGVMRGRTPSAPGACQVIGFLDTFLASNAALSVAALSADQWLAVGFPLRYAGRLRPRYAGLLLGCAWGQSLAFSGAALGCSWLGYSSAFASCSLRLPPEPERPRFAAFTATLHAVGFVLPLAVLCLTSLQVHRVARRHCQRMDTVTMKALAVLADLHPSVRHGCLIQQKRRRHRATRKIGIAIATFLICFAPYVMTRLAELVPFVTVNAQWGILSKCLTYSKAVADPFTYSLLRRPFRQVLAGMVHRLLKRTPRPASTHDSSLDVAGMVHQLLKRTPRPASTHNGSVDTENDSCLQQTH

Group A19

5H1A_HUMA ---------------------------------------------MDVLSPGQGNNTTSPPAPFETGGNTTGISDVTVSYQVITSLLLGTLIFCAVLGNACVVAAIALERSLQNVANYLIGSLAVTDLMVSVLVLPM-AALYQVL-NKWTLGQVTCDLFIALDVLCCTSSILHLCAIALDRYWAITDPIDYVNKRTPRRAAALISLTWLIGFLISIPPMLGWRT-PEDRSDP-DACTISKDHG-YTIYSTFGAFYIPLLLMLVLYGRIFRAARFRIRKTVKKVEKTGADTRHGASPAPQPKKSVNGESGSRNWRLG----VESKAGGALCANGAVRQGDDGAALEVIEVHRVGNSKEHLPLPSEAGPTPCAPASFERKNERNAEAKRKMA-LARERKTVKTLGIIMGTFILCWLPFFIVALVLP-FCESSCH-MPTLLGAIINWLGYSNSLLNPVIYAYFNKDFQNAFKKIIKCKFCRQ-------------------------------------------------------------------------

5H1B_HUMA --------------------------------MEEPGAQCAPPPPAGSETWVPQANLSSAPSQNCSAKDYIYQDSISLPWKVLLVMLLALITLATTLSNAFVIATVYRTRKLHTPANYLIASLAVTDLLVSILVMPI-STMYTVT-GRWTLGQVVCDFWLSSDITCCTASILHLCVIALDRYWAITDAVEYSAKRTPKRAAVMIALVWVFSISISLPPFF-WRQ-AKAEEEV-SECVVNTDHILYTVYSTVGAFYFPTLLLIALYGRIYVEARSRILKQ---------------TPNRTGKRLTRAQL-----ITDSPGST-SSVTSINSRVPDV------------------PSESGSPVYVNQVKV--------RVSDALLE-KKKLM-AARERKATKTLGIILGAFIVCWLPFFIISLVMP-ICKDACW-FHLAIFDFFTWLGYLNSLINPIIYTMSNEDFKQAFHKLIRFKCTS--------------------------------------------------------------------------

5H1D_HUMA -------------------------------------------MSPLNQSAEGLPQEASNRSLNATETSEAWDPRTLQALKISLAVVLSVITLATVLSNAFVLTTILLTRKLHTPANYLIGSLATTDLLVSILVMPI-SIAYTIT-HTWNFGQILCDIWLSSDITCCTASILHLCVIALDRYWAITDALEYSKRRTAGHAATMIAIVWAISICISIPPLF-WRQ-AKAQEEM-SDCLVNTSQISYTIYSTCGAFYIPSVLLIILYGRIYRAARNRILN----------------PPSLYGKRFTTAHL-----ITGSAG---SSLCSLNSSLHEGH-----------------SHSAGSPLFFNHVKIKLA--------DSALE-RKRIS-AARERKATKILGIILGAFIICWLPFFVVSLVLP-ICRDSCW-IHPALFDFFTWLGYLNSLINPIIYTVFNEEFRQAFQKIVPFRKAS--------------------------------------------------------------------------

5H1E_HUMA -----------------------------------------------------------MNITNCTTEASMAIRPKTITEKMLICMTLVVITTLTTLLNLAVIMAIGTTKKLHQPANYLICSLAVTDLLVAVLVMPL-SIIYIVM-DRWKLGYFLCEVWLSVDMTCCTCSILHLCVIALDRYWAITNAIEYARKRTAKRAALMILTVWTISIFISMPPLF-WRSHRRLSPPP-SQCTIQHDHVIYTIYSTLGAFYIPLTLILILYYRIYHAAKSLYQKR---------------GSSRHLSNRSTDSQNSFASCKLTQTFCVSDFS---------------------------TSDPTTEFEKFHASIRIPP--FDNDLDHPGE-RQQIS-STRERKAARILGLILGAFILSWLPFFIKELIVG-LS--IYT-VSSEVADFLTWLGYVNSLINPLLYTSFNEDFKLAFKKLIRCREHT--------------------------------------------------------------------------

5H1F_HUMA ----------------------------------------------------------MDFLNSSDQNLTSEELLNRMPSKILVSLTLSGLALMTTTINSLVIAAIIVTRKLHHPANYLICSLAVTDFLVAVLVMPF-SIVYIVR-ESWIMGQVVCDIWLSVDITCCTCSILHLSAIALDRYRAITDAVEYARKRTPKHAGIMITIVWIISVFISMPPLF-WR-HQGTSRD--DECIIKHDHIVSTIYSTFGAFYIPLALILILYYKIYRAAKTLYHKR---------------QASRIAKEEVNGQV-----LLESGEKSTKSVSTSYVLEKSL-------------------SDPSTDFDKIHSTVRSLRSEF--KHEKSWR-RQKIS-GTRERKAATTLGLILGAFVICWLPFFVKELVVN-VC-DKCK-ISEEMSNFLAWLGYLNSLINPLIYTIFNEDFKKAFQKLVRCRC----------------------------------------------------------------------------

5H5A_HUMA -----------------------------------------MDLPVNLTSFSLSTPSPLETNHSLGKDDLRPSSPLLSVFGVLILTLLGFLVAATFAWNLLVLATILRVRTFHRVPHNLVASMAVSDVLVAALVMPL-SLVHELSGRRWQLGRRLCQLWIACDVLCCTASIWNVTAIALDRYWSITRHMEYTLRTRKCVSNVMIALTWALSAVISLAPLL-FGW-GETYSEGSEECQVSREP-SYAVFSTVGAFYLPLCVVLFVYWKIYKAAKFRVGSRK-----------------------------------------TNSVSPI---------------------------SEAVEVKDSAKQPQMVFTVRHATVTFQPE-GDTWR-EQKEQRAALMVGILIGVFVLCWIPFFLTELISP-LC--SCD-IPAIWKSIFLWLGYSNSFFNPLIYTAFNKNYNSAFKNFFSRQH----------------------------------------------------------------------------

5H7_HUMAN MMDVNSSGRPDLYGHLRSFLLPEVGRGLPDLSPDGGADPVAGSWAPHLLSEVTASPAPTWDAPPDNASGCGEQINYGRVEKVVIGSILTLITLLTIAGNCLVVISVCFVKKLRQPSNYLIVSLALADLSVAVAVMPFVSVTDLIG-GKWIFGHFFCNVFIAMDVMCCTASIMTLCVISIDRYLGITRPLTYPVRQNGKCMAKMILSVWLLSASITLPPLF-GW-AQNVNDD--KVCLISQD-FGYTIYSTAVAFYIPMSVMLFMYYQIYKAARKSAAKHKFPGFPRVEPDSVIALNGIVKLQKEVEECANLSRLLK------------------------------------------------------------------HE-RKNISIFKREQKAATTLGIIVGAFTVCWLPFFLLSTARPFICGTSCSCIPLWVERTFLWLGYANSLINPFIYAFFNRDLRTTYRSLLQCQYRNINRKLSAAGMHEALKLAERPERPEFVLRACTRRVLLRPEKRPPVSVWVLQSPDHHNWLADKMLTTVEKKVMIHD
