# Supplementary material for: Phylogenomic Analysis Reveals Extensive Phylogenetic Mosaicism in the Human GPCR Superfamily
Source: Evol Bioinform Online. 2007 Sep 26;3:357–70. (PMC2684142)
Supplement: Supplementary Data [file ebo-03-357-s2.doc]

**Data 1. Ancestral GPCR sequences**

Amino acid sequences of ancestor sequences to each of the GPCR groups using the reconstruction methodology.

>A1anc

MEMNIQVITFYDIECKDQIAFPLSVVGLNLVILKKLSTILNASLFFLFAYRDWVGACLSMYIFYGFIMMIRLIHAFLATTGITSLTVVIFSLGIYCQENNCSYESQWHQLMILLLLIICVIRLRRERHKARIAMVFFWTPNVFNFEQLDESQDAMNTIQDDTCCINPIIYAFVGEKFRKYLLHFFQKVYRRMDYLLIDLLTPWNSPGRNMPRQSSQLSDSSRQSSSYARRLYVIHELETALFHNPLKNNVLFKTCKVFIFYILTFNMLIVELFHNYAEIGQPLFGVLRALCYGDYAFMLFGSILTDAVQSGWAFFEEHFEPYLRIIFRQTTYPAYLYVRVANGYGEPDNGILHQADLMQFKVAMLTILQTFCFFNCVK

>A2aanc

MDMMMQVSNVPMESEAFEDASPGNFSESHNNSNGLPPFNTDASMCPNEEGEHSQNINKYFLVIIYTIMFVIGLLGNSLVILVFIRARTKMSITDLYILNLAVADLLIVLTLPIWVVSMVQHNQWIFGAFLCKIMSLIYSVNMYSGIFFLTCMSVDRYLAIAHATRTSQRRPRKHMHARIVCALIWLLSLIVSLPDFVYNQTYHSNNNNATPICFFDYPNDNITKWRMVVQMLQHTFGFIVPFAIMALCYVIIVRTLIQAHQHQKHRALRMIFAVVLVFLLCWLPYNIALLLDTLQRTQIIAATCRLENRLYIALQVTDILAFLHCCLNPIIYAFIGQNFRNNLLKIIINHTSLSQLNTSSHSSSSAVSSESSEISTTSFTSAANAAPHPM

>A2banc

MGNCLHRAELSPSTENSSQLDFEDVWNSSYGVNDSFPDGDYDANLEAAAPCHSCNLLDDSALPFFILTSVLGILASSTVLFMLFRPLFRWQLCPGWPVLAQLAVGSALFSIVVPVLAPGLGSTRSSALCSLGYCVWYGSAFAQALLLGCHASLGHRLGAGQVPGLTLGLTVGIWGVAALLTLPVTLASGASGGLCTLIYSTELKALQATHTVACLAIFVLLPLGLFGAKGLKKALGMGPGPWMNILWAWFIFWWPHGVVLGLDFLVRSKLLLLSTCLAQQALDLLLNLAEALAILHCVATPLLLALFCHQATRTLLPSLPLPEGWSSHLDTLGSKS

>A3anc

MRENSTMAPTESWAPSEMQAGLDSENANNANNDNRCQAGSWKYTNVIIPTLYLIIFLLGLLGNALVVTVFHFHKGPRRVADIFILNLAVADLIFLLTLPLWATYTANQYDWPFGNYLCKIISYMISMNMYASVFLLTCMSIDRYLAIVRPMASRRMRRRRSAAVACVVIWVLAALLALPTMVFRTVQAIENTNITACAMNYPMEKNSAWEVGLGLLSNILGFLIPFLIILTCYFHIMRSLRKNYQVERKNNIRKRRRVLKIIMAIVAAFIICWLPFHIFKFLYILASLGLIRYSCQIADIIQLAMPIAICIAYINSCLNPFIYVFLDRRFRRNILSVLCGCTSRLQGRSNSSSSEKSLSYRSSNSIAPSANTPSPSWEVSQQ

>A4anc

MTSAQAISFPEPLPSRGPGSNAEPGLSVSADNGSAPNATALAPLPNPNGPPTSASAQGLAVLIPAIYSVICVVGLAGNSLVIYVILRYAKMKTVTNIYILNLAIADELFTLSLPIQATDILLRHWPFGELLCKLVLAIDHYNMFTSIYYLTVMSVDRYLVVVHPVRARHMAWRTYRAAKVVNLAVWVIATLVVLPIAVFAGVNSNQQGDRISCMLQFPNPASYWFTAFIIYTFLLGFVIPVSIICVCYSTMIRRLRAVRLRSGAKQRDRSKRRITIMVLVVLAVYLLCWTPFHLATIVNAVTDLPQTPLAVAMSYFITALSYANSCLNPFLYAFLDDNFRRSFRQILRARCSEWMGSAADQQVASEARQNTRRQAAACEEDETTEQQAAAAEEESNNLMPTSRITQI

>A5anc

MQMHDARIHQEMHKEAKRDKIANLFGRENDLREFMANSGNASAELPGPMETLVANLMNGNGSPAAPNASNAPAAGPISVENLVVPVVFAVIMLLGVVGNSLVITVIARSKPAVGKRRSTTNLFILNLAVTDLAFLLCCIPFMAVIYALDTWVFGDFMCKFVHYIIFVSMYASIFTLTAMSVDRYLAIVHPLRSQQRRTPRNALAVVLSIWALSALMSAPVLIYQRLVRFPGNQALCLEAWPDRAHQRAFHALCNFLAGYLLPLLVICLCYARMLRHLRRSLKVGPAGAAAAAGGMSQRARRRASKKVTRMVLAVVVLFAICWLPYHILQLWAAFGPHPLTPARSYLLRILAHCLAYSNSCLNPIIYAFLGRHFRKAFRRLCPCAARRSVRARRAKPSARARSHPSGGSLGHAPPSARHQSEGAAGPAPRAAAVQGPNAAPPPEPAW

>A6aanc

MWCCSLEWPRQHLGFHRRLTLIRQTNKSLWIRLHDRYCCCRHCWWNLRRLHSSAPMEREMMAMQSQGSQPEVSSSLLNPSNPTPNSEEAINLSYLSPYLHPPETRSQEALVLILAYVLIFVLSMVGNTLVCVVVMRNKHMRTVTNIFIVNLALSDLLVAIFCMPITLLQNIMDTWIFGNAICKMVSYLQSTSVSVSVFTLTCIALERYQAICHPFKAKWWLTIRRAFNIIVIIWLLALIIMVPMAVVMQVESEYYHVMPNARNRTSPHNSCQERWANDVHQKIYHTFIFLIIYLAPLALMVMAYFQISRKLYGAIRFDADRKKDAQSRKRPETTSVVQNNWSPLSQLPRSREPRQPTEPQMRSAPAPGSNSAASARQLRKKKRAVRMLMVVVLLFILCWLPIYALNMLIEYSGMSAQAAHRVTIYASFSFVHWLAYANSSVNPIIYSFMNENFRQEFLAAFSCCCPNKPPSPSQALPSSRTSAMSIKSLFQLRSSSDNATPHGELTSVNTVAPKPQQNQAGKMLKRNLARSEPECAHNPPLDPAW

>A6banc

MLMMNSAAASQGHPSPPAIASSESQMQPWDTRDPELAKVEIAVLSIVFVLAALSNASVLLALQRKGRKTSRMHLFIKHLSLADLAVALFQVLPQLMWNITYRFRGPDLLCRVVKYLQMFGMYASAYMMLVMTLDRHLAICHPLRSLRQNSGRRSHRMIAVAWVLSAVFSLPQLFIFSMRNVEDGSGVRDCWASFAQPWGRRAYVTWTALAIFVAPVIIMAACNALIIYEIHRNLRVKPQSSRQGKGAWRTGGRRRPSRLAAPSRNQSVNNISRARIRTVKMTLVIVTAYIVCWAPFFIVQMWSAWDPEAPNEESDNPAFVISMLLASLNSCYNPWIYAFFSSHLLPSPLRSLPCCANMRPRMSREPTDESSSSQSSTLNNRTSSNATLMWKDSPKLSKRIRFEEVPR

>A6canc

MCFSPILEINMQSESNITVRDDIDDINTNMYQPLSYPLSFQVSLTGFLMLEIVLGLGSNLTVLVLYCMKSNLINSVSNIITMNLHVLDVIICVGCIPLTIVILLLSLESNTALICCFHEACVSFASVSTAINVFAITLDRYDISVKPANRILTMGRAVMLMISIWIFSFFSFLIPFIEVNFFSLQSGNTWENKTLLCVSTNEYYTELGMYYHLLVQIPIFFFTVVVMLITYTKILQALNIRIGTRFSTGQKKKARKKKTISLTTQHEATDMSQSSGGRNVVFGVRTSVSVIIALRRAVKRHRERRERQKRVFRMSLLIISTFLLCWTPISVLNTTILCLGPSDLLVKLRLCFLVMAYGTTIFHPLLYAFTRQKFQKVLKSKMKKRVVSIVEADPLPNNAVIHNSWIDPKRNKKITFEDSEIREKRLVPQVVTD

>A7aanc

MRWLWPNPTALQLFLQIGEEREKGPRPLHMSPHPAETSEQQVPSARDTGYSETRGVQQSVPQDRAACLIPNNPEMYQPTKTLLMAQSQPSKASSTAFLVTNETERSASSEGSSISIKPPPGDTTTEIAFRCVIMVLYLLIFVVGIIGNATLMRIICQNKCMRNVPNIFIASLALGDLLIILICLPINVFNYLADRWLFGAIMCKLIPFIQMSSVGVTVFTLCALSIDRYRAVAKSMMKMQPIGIPLRTAVKIASIWVVSMLLAVPEAVFWDMAPFHAPNKNRSSFESCIMNPSPDLPVSKYAFMMFYQNIRMLWIFLIYFIIPLAIIAIYYTLITSTMIRKANKSLQVEENEHAKKQIQSRRQLAKTVLAFVVVFAFCWLPNHISNMLKSYNYNEVDPNMMDMISFLLLMNIISRILAFMNSCINPIALYLLSKRFSRCFMSCLCCCRSESIEQSTSTAMSNTSIKMLKNENNSSNMFTNSNVNSSHASVVKQCM

>A7banc

MTPLCLNCGTPPADMFQKAQSSPWEYNLSPEDAHSDPSPANSPLDEQRLQPEAAPALWILVPVIAVCLIIFVVGVAGNVLTVLVVSRYREKQSMRTTTNYYLGSMAVSDLLILLLGMPLDLYRFIWRSYPWVFGDLLCRLRQYLSEACTYATVLSITALSVERYIAICHPFRAKVLVTRRRVKMLIAVIWAAAMLYALPLFFLMGIEHEHESPDPSPAIVCNRSGPRHHEQPETNESRCRPFIVRRSLLNVIIWVTTIVFFFLPMICISVLYGLIARKLRRNRRPLKPSANGKTSTAARSRLNANVGQKCFIQGEHVNLSRHIESGRERGHRQTVRMLAVVVLAFIICWMPYHVNRIMFSYTSDPDWTDSMANIYQYFNLVSNIFFYLSAAINPILYNLMSQKYRAAVLKVLSARQSSHHKFHRLRDSSNSASTDNSANFFGNPEESATHQTSGNSSMENIHLTTLASEAEMQRTNHQLFHFNLE

>A8aanc

MRMENMGYNSSEATMNTNSSIPNRISESTLHVSAEITVLRILTLLIHAVAFVLGVLGNGLVIWVAGFRMKRTVTTICYLHLALADFMFTATLPFFMYSMAMGGHWPFGSSLCKLIFTIVFINLFASVFLITFIALDRCICVLHPVWAQNHRTVSLAHKVCIGIWVLALLLTLPHFIFLTTVQSTLNGHTLCYFNFSSSNDSNAEMNDWTAQERIDDDVVVAMHIVVTITRFIIGFLVPMAIIATCYALIAAKIQRQGLIKSSRPLRLLAVVVAAFFICWSPYHVVALLALVWIRAMLNNGMYPIMLIALNLTSALAFFNSCLNPILYVFMGQDFRERLIHSLPSVLERALSEVQDSTLQTSSSANNSTESSSETELQAMNECERPME

>A8banc

QNGTNEANVSTCHKQTLVLNILHWKTLIVSLCGLAGNALVLWLLGFRMRRNAFSIYILHLAAADFLFLSSRIVNSLLYFINFLVSIANSISKFLTTLMTFAYFVGLSMLAAISTERCLSVLWPIWYRCHRPPKHLSAVVCVLLWALSLLLNILEWMFCTFLFNDAEDSRALCRTVDMITVALWFIFLCPVMCGSSLTLLVRIQCCSRRMPRTRLYVTILLTVLVFLLCGLPLGIAWLLFYLFWIDLEMLFSHLHLISIFLSSINSSANPIIYFFVGSSRQRQNRQSLRLVLQRALQDEPELDAAKGSSNQETLELAASTLHQ

>A9aanc

MVPHLLMVAVHMMSPATNHQEGPASSSPMENALAVENASPSPTWTNMTLPDWQSSMSNSPANAYVQNPSEIALLAIAYGIIILFAIIGNVIVCWIIFRNRRMHTATNYFIVNLAIADLMMSVLNTVFTFVRFVNNIWIFGSAFCKFQRFFQYTSMHVSIYTMTAIAVDRYMAIVHPLQPRISITSAKIMIAVIWIMAAAFASPHAFYSTTNTMQDVRTLCLVEWPENPNGIFLKVYNLIVFVLIYILPLLIMFVAYARVAITLWRSEIDGDTAQRYHNIHPRTKKKIIKMMMIVVLLFAICWLPFHIYIILPSFNQDIYVYKTNNQVYLAIHWIAMSSTAYNPIIYCCLNDRFRIGMKQAFRMCQWVSMSSYDGNEYTTTTSLRMRQSNMYKISRMHTMATIFTVASTEESEDRSAPEKKNQSPINQSPSGSSISNSKEATETMSFISNPHTSVDEYS

>A9b

MTMGFMRDNMMSTSTSDLNMMNPNGANATSSHSNQTNARATPNGSEPVKLSDSQKLIALQIVLAMALVIIILVGVIGNLLVILAVVRNKKLRNATNIFIVNLAVADLLVAILCYPFILVYILIDQGWDLGELQCQMVNYLMGLSVIVSIFTITAIAINRYHVICHPLRSQMIYSIRNSALYIVLIWILTVLIVLPNMIFTIETNLQETYHVVTIEYDSRTYSCTQIWPGDQQLANTAYTVSILVIQFVLPLVIISFCYVRIWIKLWNRAQPACPNSMMPEMRRNKLIDRQLAEIRRNFLMMMCILVVFAVCWAPLHVITLVRDIDPQEMDPKHYNLVFLVSHLIAMFNSCLNAIIYGLLNENFRREYKKIFLALWNSRVIHPESSTDINAKQNEMPAASMRNNNRVKAATINRNHDCEKI

>A10anc

MPVPLGLPVFPAKALMAAAPPSLVLLPLAPSHCHPPRDCHSNRPFNCTEKGVTEIPSNLPASTQYLDISMNNITQLPENPFPNFRFLEELRLAENNLSYIPKGAFSGLNELEKIEMLQNNTLKQIESNAINNLSSLQEIRIQNANHITYIDPDSFENLHQLRYLSISNNAIKQIPDVHPIHSLETLFILDIADNINIHSIPGYAFTNLSSESVILRLHNNKIQEIQNHCFNGLHNLETLDLNYNNLDEFPQAIRTRPNLDELNFNDNNIRVIPDKAFDGNPLLRTIHFYDNHLEIMNNDAFHNLPDLHTLTIRGASMIQQFPNLTGTVHLESLTLTGTQISSIPNNLCNQLPNPRTLDLSYNNIRDLPSFNVCHKLQEIDLRHNQIYQIKEDTFQQLISLRILNLAWNLIHEIHPRAFATLPPIINLDISSNKLTSFPTYGLNNLNQLRLTGNYSLKELISSENFVNLMEISMPYPYHCCAFRGCENYEKWRRQYKEISNIHNKENNSSQNDSMQQESGMRNAANETNTEDFEEDFQMINSDYDYDICNEVEDVQCTPKPDAFNPCEHIMGSNMIRITIWFISILAITSNMIVILIVLTSHYNLSPPRFLMCLIAVANLFMGIYTAIIASVDAHTWGRYHNHAIWWQTGSGCNAAGFFSVFASELSVYLLTVITLERSFTVKYAMRLDRKIQLRQLRAIMLMAFLFALVAAAFPLFGRSEYSKVPICLPMDTDSPPSMVYIVTLIILNVLAFLMMAVCYIKIYINLENEDLMSNNSDSRMVKHIALLIFTNYIFMCPIAFFAFSAAINVTFITPEIMKFILLIFYPINACLNPLLYVIFNPNFRRDWIILRRRITVYSRQVQVYRSQQVSSINSDDINNQNCNSNQQVSLFTMSSIHSLIQASQNSSKHTEVTEECHMQSVLFCQCP

>A11anc

MLMAESLMLSRNSLQSSSPEMMNNNPCEMLSDFKSILILMVYILIFIVGLLANALALRVFLSRIRRRNPVATHIYLFNLALADLLLALALPFMMIYYLKGFHWPFPNVACPLTSFIFYSSMYVSILFLSAIAINRYLGVMHPLHSLHNRRRPRYAAIVSLAVWLLAFGLIIVVIIIQYLNITEHIRPQQGNETTCYNNYRDNELAVMISYRLALAVVQFVIPLIVIIFCYSRFVWALRRRPMATARGEHQQRRRRVAALLAVILINFIVCFLPYNIMRVIMHYIQRLDGQSPANSCQVLNYIIQTYNIATSLASLNSCVDPILYYFSSPVFRRSFHRLFRRLRGSWMQWNGESSKDTAEETNGDRNRADGPAEPQSSERTESSSAGSQGATANNH

>A12anc

MTFNMTLTKLPNWEYHGHRMQINTNHTQQPNNSFICPMDNELQSIVFPILYSIIFIVGIIGNGFALWVFIQIHSNRNSIKIYLINLVIADLLLIITLPFRIVNHANLGNWTLPVFLCKVAACLFYINMYISIIFLGFISIDRYLKITRPIKTSRINNPTRAIILSVVVWMLVLLAMLTMMILTNKNTKENNVIKCMEFRDEFGSKSHLIINFIIVVMFWIVFLIIIICYIMIIRNLYRNSKKRKSSNSSNKRKALRNIFIVVAVFIICFVPYHVVRIPYTLSQLNNVTDCNSREIIHKANEITLILAATNVCLDPIIYFFLSKNFREKLCEKFNRRRSSESIRSESRSEQTEVITPNDETPALKIQN

>A13anc

MQYQDIYLDMASAFAVFPNNFMVTILAGQPAAAMMTAAGSPQAPESGQPMSQCFYNETIDSHNNHINKQLGSQESDIACGPQLTAVAVLSLTLSLLIVLENLLVLAAIASNRSFHRRPMYYFIGNLAVADLLAGLAYVYNIINTHVINSGYLTARASLSQNQSFLIEGLLIMSFTASVASLLAIAIDRYISMMYPLAYYSITTRGRAVLLIGLVWTIALVLGLLPVLGWNCLSALDACSEMFPLYSKAYLLFWVLFFAMVFLIMVALYVHIFRLVRSRAQRMPQRPATRHRAGPGRPRRQSMNMRLLKTLVIVLGVFIVCWGPLFLLLLLDVFSPARQSAVLYLAQYSLLLALIMLNSMVNPIIYAFRSKELRRAFRQILCSWCSCSSMRDPNAERQRAVIASGSASSSDSSSNNIRDGRRTNEPDIRNTVKSASVTNSSSTDTSAEAAN

>A14anc

MNHCYPMSWSMSGSAMSSSNATTSATLSYDRLGVSPAFPAIMMVAGIVGNLLALALLARSSRVWCYRRRRPPRETAFYVFVCGLALTDLLGQLLVSPVVISAYASGQSWIAISPGGNRLCTYFAIVMTFFGLSSLLIASAMAVERYLAISHPFFYAQHVSARAARLALAAVWASSLLFCLLPLLGLGQYQLQYPGTWCFINLGSEEGGHANWGNLFYALLYAFLMALLITVTFLCNLLTILALCRMHRQRQQRRPSPGRRRWHAHAPRSVAARGHSAPASASAFAGDSRRRRSARRSGAEETEMVIQLMAMTVVMAVCWIPLVIMVLMTVFNQPSVMHCATHLERETEENELLQALRIASLNQILDPWVYILLRKAVLRRFIQIIYHRCSRVSSSTSESSQSSSSLSSRDSSAMEAPVSNFSASEQLEISAEGQTEPPDPTQQDLGENVLTGRNLEPGVPCMLC

>A15anc

MRMPAVAFALAAAIPMMDRRSIDFMSNDSNSNATPTPRIFLFRNPNDNDFEEFEFGMIEPMMGTNTNSSATPQAINCNFTSNDSFAYNLYAWLCVFLMAVYSLVFIVGLVANSVALWVFCCRMKKRNETVIYMINLAVADLLFVLTLPFRIYYYINHNHWPFGDLLCQISGTLFYLNMYASILFLTCISVDRFLAIVHPFRARSLRRRRYAKGVCAAVWILVLSASLPAPLLQSTNREYCRYVNNETTTCFENFSEKTWKAVLFRNIVIIAEVVGFIIPLIIIVFCTSKIIRTLRQQPATAHQGATERKKAIRMIFMCLVIFIVCFTPYHINLILYALLRSTISNIASCPARHAVRFMYHITLCLASLNCCLDPIIYYFMADSFRNSISRMFTQWKEPNRSVTRRRFMSKESLEASSSRIQHNSQNVKSQTLNNGGEASLESRFIPAFTQCNQPSAKMFPPAR

>A16anc

MAQQWSLQRLAGRHPQDSYMNSTEEPNFYLPMLRSNLVNRSDMENPDAHIASRSEFNMLAAYMILLVLIGIPLNALTLAVFIKYKELRTPTNYIILNLAVADIAISLIAYPMSITSSLYGSWPFGHAGCAVHGFLGILAGLVSISSLAVIAWDRYLVICTPMGNSRMASNSAITLVLFAWISALAWAAMPIIGWARYAPEPLQASCTIDWSKGNSEVNSTSYTMYMFVFHFIIPLIIMFYCYSQMLLSIKEATADCQESLNKDRSEQIDVTRMVVIMVIMFLVAWGPYAIVCMYAVINQPNSFNPRMAMIPAFFAKSATIYNPIIYVMANKMFRRAMLQMFSCQKNQMDPDSEISSMDKTQTPTVSSTQPA

>A17anc

MDILCEENTALGATPNMLAAMMVNASSAANNALVAEAGLQMAAANTSDADNSAIQTASASAASGGASESQAGKQNLAALLIVMLIIMTIAGNTLVCMAVALERKLQNNPTNYFIMSLAVADLLVALLVMPLSLYTEMMGARWPLPRVLCDAWIFLDVMMCTASIMHLCAISLDRYIAIRMPIQHNRRTGRSPTKAFLLITAVWLISIAIAIPVVIFGLQDEGNWTPSDEETDKKSPNNTTCRLTADRDYVLFGSLVAFFIPCAIMVITYFATFHVLQRQAIRVIHKQSNRPPRASMSPSFQSDNTPCPRMPQGPCGPECAPEAPGLSRGPEERRCAQAREEKAPDPSAPSCAAPASSAPPSRHPVSGPSPANEAPQGPPDPEAEEECEPQPQDPCPPDPSAKLAPASTAPSSNSKNATQSANARRSKPTSTRRRRSMQATSRERKATKVLAIVMFLFLLMWTPFFITHILSVLCDENCNQFCNVMEMLFNVFTWIGYVNSALNPLIYTLFNREFRNAFRRIIRRCRYRASRRPKALRRVSNIPASRNQSRELQKSQEKAGKRPASNNANANAPGVASNAQNLEASINAPVGANLKISSGDREEAQVGAFNADPAEPAAQPSPLSIQHHAGPAIKVQAISAPRSEEEAVGLGNPHCEAEADTCNAHELFDYNMPREPDQ

>A18aanc

MASYNPVNNSLNNSSCLLEDNACAGNKATFQLMFIVIVLGIICLVTVVLNILVMIAIRVNRQLQTVNNYYILSLACADLIIGVFVMNMYILYILMGHWALGSVVCDFWLAMDYVASNASIMNLFIICFDRYFSVQRPLRYPKYRTTKRAGLMIAAAWLLSFVLWVIPILFWNYIMQQRTVREDQCYIDFYDEPTIKFMTAIINFYLPTIIMLIFYWRIYRATRNHVQQHKAINPSEQEFKEEKFRPESIMSQASKNRPESPSAMQRNRNQDAGSSSPRSTPSPSPPEMETMPQANSANDSQLNNISSYPLENDEDKDEEDERSQPVSEDSADEPPMQAASANSSRRAVASNRTNEQAEQDENSLNTHPASENPENQMLTAIRIFMRTDEDTTSETTNTTSKLKATSSPASDEIKSTAKRLIAHTRQHMAKRKRMPMARERKAARQLFFIMLAFILCWIPYFIMFMVNTFCKNCINEHLWNITYWLCYINSTINPMCYPLCNENFKKTFKMILHCRWKNRKSTRKQYRQGNVHIPHKRQC

>A18banc

MPMMLEMANAVYITMEILIAVVAIVGNVLVCWAVRLNNSLQTATFYFIVSLAVADIAVGVLVIPFAIVISLGITTHFYSCLFMACLVLILTQSSIMSLLAIAVDRYLRIRIPLRYKSLVTPRRARVVIAVCWILSFVIGLTPMFGWNNKNAAKNAWDANQNRGESLIKCQFENVIPMNYMVYFNFFAWVLPPLLLMVVIYLKIFYVIRKQLNRKVSMSGDQSQTYYQREIHTAKSLAMILFLFALCWLPLHIINCITLFCPNCKHKPQLLMYMAILLSHANSMMNPIVYAYRIRDFRETFHKILKSHVLCQPEPFPADNSLEKNQAAHGVDLPERRPND

>A18canc

MANTSHEPDEVSGSLGPEAASLFLKLATLGLIMCVSLAGNVLIALLIVRERSLHRAPYYFLLDLCLADGLRALVCFPFVMNAVRHAASWTFGALSCKIIAFLAVLFCFHAAFMLFCIGVTRYLAIAHHRFYAERLTFWPCAAMICMAWTLALAMAFPPVLDVGTYNFIREDDQCTFEHRSFKANDSLGFMLLLAVIMAATHLVYLRLLFFIHDRRKMRPVRMVPAVSHNWTFHGPGATGQAAANWIAGFGRGPTPPTLLGIRQNGHATGRRRLLVLDEFKMEKRLSRMFYAITLLFLLLWGPYIVACYWRVFVRAPAVPQAYLTAAVWMTFAQAGINPIVCFLFNRELRKCFRTPFLCCQSARQPRHPCCLM

>A18danc

MTLNCTNSCREENSNHTCMELGEHGIIRTQFIVIFLITIFVGNIVIVLTLHRKPHLLQLTNRFIFNLLLTNFLQIVLVLPWVVTTSIRRFWIFNVHWCNFLVLLYHLFAFASMNTIVVIAIDRYYAIIYPMVYPMKMTQRRAYMLLYYIWIHAIIQCLPPLYGWGQVEFDEFNWMCVMIWHREPGYTIFWQIWYILFPFIVMIVCYGFIFCVARVKRHKLHCRTKDCVENDDQREGRKNGSTGTSESGVRRRAFQEVRYSANQCKALITIFIIIFAYMLTWGPYCFIAVEALWVETQVPQWLETWIIWLFFLQCCCHPYIYGYWHKTIRKEIQDMCRLFFCKNRIPDEDLHPHLPALMAGTQPLFVCSHDSANFR

>A18eanc

MANPEVLNRDEVAESLALFFMLLLDLTALLGNAALMVVILRTPALRDFLYLFHLCLVDLLAALTIMPLAMLAAPPLFDHVRFGEVPCRLYRFLSVCFLPLCILGVAAINLERYYYIVHPMRYEVRMPPVLVLTAVWVKALLMAALPLLGRPPWEEPAPAHCSLQWGHGPFRPLWALLYFLLPLLLILLVYCGMFRVARRAAMRDPRPPRWMRLRRDRLDSRLAIQPPPHRRFPGGKAALVPLLVVGQFLLCWLPYFCFCLYPAARVEAVVTWIAYFCFTAHPFFYGCLNRQIRLELGRQFRCFFPPPPRECLRQPWHERAIEQCFQRFPQGPACPPEEWPERPPELPKQRPPAYDFRIQAR

>A18fanc

MECMAPTLEAYLYLNLNTVNTTDAAFKQLNLPLRITLAIIMIFMLFVGFLGNLVVCIMVYQRPAMRSAINILLATLAFADMMLALCCMPFTLVTIITTRWHFGDHFCRLSAMFYWFFVIEGVAILLIISIDRFLIIVQRQDKLNPYRAKVIIAVSWVLSFCIAFPLLTGWPDLQIPARAPQCVFGYTENPAYRAYVILIVLIVFFIPFLVMLCAYMCILNTLRHNALRIHNHPDGICLRQLTRLGLMRLQRQFQMSIDMGFKTRAFTTILILFVVFILCWLPHTTYSLLATFSQHFYCQHNFYEISTWLLWLCYLKSVFNPIIYCWRIKKFHDACIDMMPQTFQFLPQLPEHIRRRIRPSTVYCCNEHRTVV

>A18ganc

MMSTLDVNQSEHHFCPLAFGYYETVNFCIFETLIIVFLTFLIIAGNIIVIFVFHCAPLLHHYTTSYFIQTMAYADLFVGVSCLVPTLSLLHPLPVHESLTCRIFGYIISVLKSVSMACLACISIDRYIAITKPLTYNQLVTPWRLRICIFLIWIYSCLIFLPSFFHWGKPGYHGDIFQWCAESWHTDAYFTLFIVMMLYAPAAFIVCFTYFHIFRICRQHTKDINDRRARFSHQVDETRETQHCPDRRYAMVLFRITSVFYMLWLPYIIYFLLESSRNNRFAFLTTWLAISNSFCNCVIYSLSNGVFRRGLRRLFETMCTSCMCQKDQNDQYPKRRKRPNNCCH

>A18hanc

MWNGSDLNFCCYHESWLGYRYPVVLWYVVLAITGTLANVMTIYLLAIFRKLRTRFNLFIVNLCLADLLYCTLWMPFEVDLYLHLHWRGATFCRLFGLLLFLGNTVSILTHCLIALNRYLLITHPKLYQQLYQRRHIVLMLVLTWVLALALFLPWPIYILVEPPYPTILMAIYFLLQLALLLHYYCLIHRRVRRAVQRLDQYNFHQLHQHPNCVARTDEFMPARHQELDGRLAHPAQPQPLPRRLQDRRAQFRLVTRMCFLLYCCFLLAYQPFLWLNILDAFVVPWVVHMLAWNLCWLNGCINPLLYTWMNRQFRRAYRSILPRVPRAFHRLH

>A18ianc

MGPGEALLAGLLVMVLAVALLSNALVLLCCAYSAELRTRASGVLLVNLSLGHLLLAALDMPFTLLGVMRGRTPSAPGACQVIGFLDTFLASNAALSVAALSADQWLAVGFPLRYAGRLRPRYAGLLLGCAWGQSLAFSGAALGCSWLGYSSAFASCSLRLPPEPERPRFAAFTATLHAVGFVLPLAVLCLTSLQVHRVARRHCQRMDTVTMKALAVLADLHPSVRHGCLIQQKRRRHRATRKIGIAIATFLICFAPYVMTRLAELVPFVTVNAQWGILSKCLTYSKAVADPFTYSLLRRPFRQVLAGMVHRLLKRTPRPASTHDSSLDVAGMVHQLLKRTPRPASTHNGSVDTENDSCLQQTH

>A19anc

MDEPADQCAMSLPPNNQSSVQASNASSNPAQNCTANDYSYDSPITMPLKILIAMLLSLITLLTTLINLFVIATILRTRKLHQPANYLIASLAVTDLLVAILVMPISIMYIVMGRWTLGQVLCDIWLSMDITCCTASILHLCVIALDRYWAITDAIEYANKRTPKRAAVMILIVWIISISISIPPLFWRSHQKTNPDMDECVINQDHISYTIYSTIGAFYIPTLLMIILYYRIYRAARSRIQKRKFPVFPRVEDDRHIASPSRVAKQSTNAQVNSFSWILGSAESSTSSVSSIYCSNPEVHSSSASTELNSNHASIRIAPSEFDRKNDRSSERKKISAARERKATKTLGIILGAFIICWLPFFIVELVLPICKDSCNIPLLISDFFTWLGYLNSLINPIIYTIFNEDFRNAFQKLIRYRCSSQ

**Data 2. GPCR tree**

Newick format tree of 257 GPCR sequences, using the ancestor reconstruction method outlined in the methods.

((((GPR78:183.85049,(GP61:134.735,GP62:134.735):162.81032):14.81521,(GPR101:122.825,RE2:122.825):180.46423):14.81521,((((RGR:153.55912,OPSX:103.13095):57.230155,((OPSR:5.74229,OPSG:1.56342):62.53967,(OPSD:70.06064,OPSB:83.54091):23.97372):57.230155):150.23597,((PE23:108.97344,(TA2R:99.86795,(PF2R:110.24989,PE21:83.19199):25.70419):36.33971):32.75526,(PE24:108.39969,(PI2R:85.36587,(PE22:68.11989,PD2R:96.88321):28.27757):44.52314):32.75526):159.2677):9.07202,(((SREB3:41.225,GP85:42.415):37.5175,GP27:37.5175):175.24678,((((((EDG4:65.3443,(EDG7:77.17748,EDG2:52.09727):12.82488):64.00293,(EDG6:113.35678,((EDG8:91.28959,EDG5:76.91483):19.86509,(EDG3:61.74545,EDG1:57.55148):15.88984):25.1591):42.57051):20.790505,((CB2R:122.93923,CB1R:59.04613):102.02863,(((MSHR:95.3627,(MC4R:43.77469,(MC5R:38.42923,MC3R:53.37691):17.87149):11.07461):23.12165,ACTR:73.41356):76.62572,((GPRC:55.25993,GPR6a:53.29154):19.62355,GPR3a:34.87473):112.28239):30.36636):20.790505):145.52297,((AA2A:41.48912,AA2B:57.21642):32.013305,(AA3R:86.03568,AA1R:45.98892):32.013305):122.20657):23.16962,(GP52:41.37,GPL:41.37):199.06772):17.59317,((((D4DR:117.66248,(D3DR:64.97992,D2DR:73.87492):50.94074):29.05275,(((((PNR:72.32608,(GPR58:40.0499,GPR57:56.57808):74.38692):22.35684,GPR102:77.15724):99.00934,(DBDR:63.81829,DADR:53.32235):88.01417):23.39284,(HH2R:110.1835,(5H6:128.30141,(B3AR:73.15126,(B2AR:102.35666,B1AR:42.05349):30.27245):59.3527):21.73068):10.78855):21.94741,((A2AB:67.94309,(A2AC:68.01722,A2AA:74.25455):14.5058):78.16926,((A1AD:85.81288,A1AB:82.69424):16.03422,A1AA:85.50123):76.53919):22.33024):24.39247):62.616455,(5H2C:60.08999,(5H2B:112.95151,5H2A:68.81478):15.9316):62.616455):121.90481,((((ACM3:63.31336,(ACM5:70.32379,ACM1:69.15941):20.25875):27.95682,(ACM4:59.02588,ACM2:53.14833):47.14845):108.892145,HH1R:108.892145):161.6826,((5H7:104.7647,5H5A:121.73667):21.25654,(((5H1B:36.90956,5H1D:49.59181):40.88358,(5H1F:46.4977,5H1E:62.47836):37.03171):27.7218,5H1A:91.68289):21.25654):83.91266):25.4821):17.59317):128.44577):9.07202):99.32502):64.167015,((GPR84:188.55,GPR88:188.55):174.68052,((GP63:63.505,PSP24:63.505):194.468,(((((GPR48:74.76364,GPR49:62.33904):90.361325,((TSHR:82.13777,LSHR:61.44195):28.41702,FSHR:45.56937):90.361325):241.86091,GP22:221.00066):47.42868,((((CCKR:57.42509,GASR:83.8232):103.27275,(FF2:81.93178,FF1:57.28869):70.41728):11.531215,(GPR103:170.945,(OX2R:44.5705,OX1R:42.69476):105.60704):11.531215):64.1925,(((L1X:70.62358,(L1B:61.86273,L1A:47.04058):39.37681):75.93712,((NY5R:133.14086,(NY4R:87.16732,NY1R:68.729):52.45764):22.93192,(GP10:120.86894,(NY2R:105.07323,PKR1:182.51447):21.77984):20.4582):75.93712):97.915,(((NK4R:32.07341,NK3R:7.13462):37.27049,(NK2R:94.9429,NK1R:44.77781):13.38484):56.64686,(GP19:201.13263,GP83:104.45146):56.64686):122.455):64.1925):102.89125):21.04261,(((((UR2R:168.30484,(SALPR:169.42449,(P2Y7:116.32636,GPO:144.11136):32.39077):18.47133):37.50945,GPR54:114.41579):10.881745,(GALR:102.26661,(GALS:61.16192,GALT:60.50658):44.57343):10.881745):105.71443,((GPR8a:35.12389,GPR7a:48.37246):33.26334,((((SSR5:49.20677,SSR3:66.08157):14.63704,SSR2:72.78239):18.14464,(SSR4:47.3169,SSR1:43.67938):37.81176):51.07906,((OPRM:53.53133,(OPRX:81.8717,OPRK:50.93849):15.07375):16.40741,OPRD:34.54116):67.90721):33.26334):132.40215):39.54429,((((V2R:114.54569,V1BR:59.22453):11.75585,(V1AR:64.30627,GRHR:197.05971):11.75585):89.85,((((TRFR:172.46193,(NMU2R:85.49031,NMU1R:81.87982):52.71081):24.03308,((NTR2:98.27594,NTR1:75.98765):30.78195,GP39:162.75526):47.27188):32.807485,(TLR:68.16583,GHSR:82.51509):32.807485):124.03,((NMBR:56.44623,(GRPR:55.4543,BRS3:79.91228):21.54556):51.983,((ET1R:65.00596,ETBR:48.36223):70.56857,(GP37:79.58788,ETB2:86.59444):149.55417):51.983):146.27):89.85):143.92736,((DUFFY:281.1842,(((((74hMRGpse:27.30278,73hMRGpse:43.13976):16.52427,((70hDRR3:1.95696,(69hMrgX1:1.15983,68hDRR4:1.0E-4):1.54082):9.12773,((72hMRGpse:25.34324,(64hMRGpse:26.46884,((67hDRR5:2.57603,(66hMrgX4:1.0E-4,65hDRR6:1.15828):2.10176):20.75497,(62hDRR2:7.12182,((60hDRR1:3.55657,63hMRGpse:21.31079):0.52562,61hMrgX3:0.0):0.55991):8.7154):3.39156):2.08646):4.69853,71hMRGpse:21.44102):4.42612):16.24584):13.30217,(78hMRGpse:97.50327,(76hMRGpse:44.09499,(77hMRGpse:50.66777,75hMrgX2:33.23445):8.99887):3.24939):2.86295):25.88267,(MRGhum:128.8974,(82hMrgG:126.80398,((80hMrgE:111.47679,8hMrgD:77.19859):20.44865,(5hMAS1:116.41327,3hMrgF:92.42831):25.53742):11.09232):16.23099):25.88267):177.01317,((FML2:29.80141,(FMLR:54.88975,FML1:13.05298):16.72755):31.41194,(GPRW:133.55598,(GPR44:124.42837,((GPR1a:123.77168,CML1:85.84976):16.99915,((C5AR:85.77456,C5L2:119.29569):39.03092,C3AR:92.07901):31.21561):15.02322):34.17871):31.41194):99.27464):27.37273):28.98219,((((HM74:96.40193,(GPR43:1.21387,GPR42:1.90267):80.30317):47.993525,(GPR41:159.14834,((((GPR81:125.62037,GPR91:104.69765):34.23953,GPR80:84.51352):29.34234,(P2YR:109.8517,(P2Y6:61.31863,P2Y11:68.95531):43.78178):26.09601):32.19253,(P2UR:213.85027,(P2Y4:105.24826,GPR82:106.91951):69.47792):20.53045):25.196):47.993525):57.95,(((H963:119.62132,((GPR87:94.89033,KI01:62.42921):15.55643,(GPR86:79.63163,P2Y12:60.52742):28.17752):37.95739):25.458125,(PAFR:139.36685,GPR34:111.70499):25.458125):124.24,((P2Y10:133.50254,(P2Y9:61.06541,P2Y5:61.36563):52.31937):10.37144,((GPRK:152.79994,(GPR92:113.79663,(GPR55:142.61391,GPR35:126.33077):28.17378):13.34348):22.23422,(((((PAR3:117.49887,PAR2:99.51218):16.572,PAR1:117.84677):48.92198,(GPRI:152.92509,GPRH:100.97169):22.6894):12.53257,EBI2:151.50612):10.62822,((GPR65:109.59987,(GPR68:83.39547,GPR4a:64.67916):30.30548):21.65233,G2A:131.85236):67.02371):20.83984):10.37144):88.33):57.95):63.8,((((GP15:124.97203,GP25:116.30447):38.59305,APJ:120.73022):23.44439,(((BRB2:110.80267,BRB1:103.45843):56.79692,AG22:113.08117):17.40167,(AG2R:1.0E-4,AG2S:4.43059):101.56945):23.44439):66.267,(((CML2:177.36146,(RDC1:104.07656,ADMR:145.80185):47.18895):23.926895,(((CXCR4b:117.8541,(CXCR5b:112.4188,(CKRA:125.75444,CXCR3b:74.45582):22.08971):23.9398):12.72183,(((CKR9b:93.10282,CKR7b:78.10782):16.63076,CKRB:121.98647):20.4368,(CKR6b:87.55657,CXCR6b:122.90997):21.71704):25.70677):23.07936,(CXCR2b:20.99209,CXCR1b:30.82583):66.56169):23.926895):52.114,((CKR4:69.68091,((CKRX:119.25041,(CKR3:55.2607,CKR1:31.97596):19.4358):16.23815,(CKR5:27.6287,CKR2:49.95098):28.61857):29.70368):16.73481,((CXC1:131.27627,CKR8:74.33114):10.85415,C3X1:102.1272):16.73481):77.182):21.445):63.8):97.09067):27.92567):19.23506):27.34303):25.85752):18.95292):64.167015);

**Data 3. Phylogenetic event types**

These tables represent the actual and standardised number of counts of outcome types for HTTP analysis with the real GPCR data and (null hypothesis) simulated data set. For an explanation of the phylogenetic outcome types 1-19, consult supplementary figure 2.The standardised data sets adjusted counts as a proportion of 200 counts per group in order to be able to compare the frequency distributions between the null hypothesis and real data. In this case groups in which there were less than four members were excluded because many of the phylogenetic outcomes were not possible and would skew the over all data set.

**a. GPCR real data**

Group Event types (1-19 in order) Σ

GroupA1 51 52 0 3 0 7 3 0 2 1 0 1 0 0 0 0 1 32 0 153

GroupA2a 64 48 6 6 0 12 9 0 10 6 3 12 10 11 3 3 3 60 0 266

GroupA2b 0 0 0 0 0 0 0 0 0 0 0 0 0 5 0 0 0 9 0 14

GroupA3 45 10 3 1 0 4 3 0 1 2 1 1 5 8 5 0 2 44 9 144

GroupA4 81 46 4 1 1 11 5 4 2 3 1 4 2 8 4 0 7 41 6 231

GroupA5 37 20 1 4 1 9 6 3 1 3 0 4 4 8 15 1 5 63 7 192

GroupA6a 74 1 1 0 1 4 0 1 0 5 0 3 6 24 21 0 15 29 11 196

GroupA6b 13 10 0 0 0 0 0 0 0 0 0 0 3 10 1 0 4 30 9 80

GroupA6c 0 0 0 0 0 0 0 0 0 0 0 0 0 11 0 0 0 8 0 19

GroupA7a 57 9 8 0 0 7 1 1 0 4 0 0 10 46 18 0 9 25 8 203

GroupA7b 46 14 2 3 0 6 1 2 1 7 5 4 18 15 17 1 9 48 1 200

GroupA8a 50 42 0 5 4 8 3 0 3 5 2 4 6 55 0 0 8 47 8 250

GroupA8b 140 168 1 11 11 12 6 3 18 0 2 2 0 49 0 0 0 27 0 450

GroupA9a 58 9 0 1 0 1 0 0 2 3 2 5 7 11 3 1 3 25 1 132

GroupA9b 59 17 3 0 2 12 2 1 0 15 0 5 19 102 18 1 15 51 2 324

GroupA10 91 20 5 3 0 4 0 0 0 4 0 1 21 13 18 0 12 27 26 245

GroupA11 78 29 4 8 0 20 16 2 8 16 0 5 7 7 10 1 8 45 9 273

GroupA12 26 28 1 2 0 3 3 1 1 3 1 1 0 5 5 0 0 38 1 119

GroupA13 117 78 4 12 1 39 26 8 8 17 3 26 6 20 26 0 0 23 0 414

GroupA14 38 26 1 2 0 12 1 0 2 0 0 1 6 24 28 0 11 37 11 200

GroupA15 66 33 3 13 0 14 31 5 17 13 4 21 3 17 36 0 10 87 1 374

GroupA16 44 3 1 2 0 4 3 0 2 2 2 0 2 0 3 1 1 20 6 96

GroupA17 183 57 11 14 14 32 22 13 17 12 12 44 26 51 170 0 24 69 11 782

GroupA18a 46 18 1 0 0 1 0 0 0 3 0 0 16 22 9 0 15 30 7 168

GroupA18b 24 4 0 0 0 0 0 0 0 0 0 0 3 1 4 0 2 30 8 76

GroupA18c 0 0 0 0 0 0 0 0 0 0 0 0 0 12 0 0 1 2 36 51

GroupA18d 0 0 0 0 0 0 0 0 0 0 0 0 4 6 0 0 1 21 18 50

GroupA18e 0 0 0 0 0 0 0 0 0 0 0 0 4 2 0 0 3 19 10 38

GroupA18f 0 0 0 0 0 0 0 0 0 0 0 0 4 4 0 0 4 8 18 38

GroupA18g 0 0 0 0 0 0 0 0 0 0 0 0 2 0 0 0 1 1 28 32

GroupA18h 0 0 0 0 0 0 0 0 0 0 0 0 12 2 0 0 3 17 6 40

GroupA18i 0 0 0 0 0 0 0 0 0 0 0 0 0 5 0 0 0 11 0 16

GroupA19 48 20 1 2 1 2 1 0 0 0 1 1 10 23 9 0 1 45 10 175

Σ 1536 762 61 93 36 224 142 44 95 124 39 145 216 577 423 9 178 1069 268

**Total 6041**

**b. Simulated GPCRs**

Group Phylogenetic event types (1-19 in order) Σ

GroupA1 54 16 4 3 0 16 8 0 3 6 0 4 0 2 0 2 1 19 6 144

GroupA2a 46 22 9 17 0 36 19 17 20 18 2 23 0 0 0 5 4 14 0 252

GroupA2b 0 0 0 0 0 0 0 0 0 0 0 0 0 11 0 0 0 7 0 18

GroupA3 38 5 2 8 0 6 3 3 5 13 2 18 0 0 0 2 8 21 10 144

GroupA4 71 18 15 8 0 31 20 8 9 3 0 2 0 0 0 0 0 13 0 198

GroupA5 19 8 0 5 0 3 2 1 2 13 0 11 1 7 0 1 12 44 15 144

GroupA6a 10 2 8 2 0 5 1 0 1 11 0 2 1 4 0 0 5 30 44 126

GroupA6b 4 2 0 0 0 0 0 0 0 0 0 0 2 18 0 0 11 28 7 72

GroupA6c 0 0 0 0 0 0 0 0 0 0 0 0 0 12 0 0 0 6 0 18

GroupA7a 15 3 3 1 0 2 0 0 1 12 0 3 1 6 0 0 7 39 33 126

GroupA7b 17 3 1 2 0 4 2 1 0 18 0 7 4 5 0 1 14 37 28 144

GroupA8a 27 19 9 4 0 33 18 9 12 9 1 4 0 0 0 0 5 30 0 180

GroupA8b 220 80 3 12 0 36 21 14 22 6 1 4 0 0 0 2 0 29 0 450

GroupA9a 39 7 0 4 0 0 0 0 1 8 3 7 0 1 0 9 3 22 4 108

GroupA9b 21 10 3 5 0 8 7 3 3 14 0 9 1 9 0 1 21 28 19 162

GroupA10 2 3 1 0 0 0 0 0 0 2 0 0 0 12 0 0 5 24 41 90

GroupA11 25 12 6 3 0 18 9 4 15 29 4 32 2 7 0 0 20 31 17 234

GroupA12 30 17 2 5 0 7 7 0 4 7 3 10 0 2 0 1 6 22 3 126

GroupA13 46 19 10 5 0 75 40 37 16 35 0 14 0 0 0 0 4 23 0 324

GroupA14 14 13 1 6 0 7 15 1 0 9 0 6 0 6 0 0 17 34 15 144

GroupA15 29 13 9 11 0 41 23 11 19 56 3 52 0 3 0 3 10 22 1 306

GroupA16 36 9 1 3 0 6 2 0 0 10 1 5 1 2 0 2 2 19 9 108

GroupA17 31 13 21 18 0 105 34 47 30 55 0 35 0 0 0 0 2 23 0 414

GroupA18a 19 7 2 10 0 5 2 0 0 2 2 5 1 8 0 1 11 19 14 108

GroupA18b 36 1 0 0 0 0 0 0 0 0 0 0 0 1 0 0 3 22 9 72

GroupA18c 0 0 0 0 0 0 0 0 0 0 0 0 0 4 0 0 1 15 34 54

GroupA18d 0 0 0 0 0 0 0 0 0 0 0 0 3 0 0 0 1 22 10 36

GroupA18e 0 0 0 0 0 0 0 0 0 0 0 0 8 0 0 0 4 16 8 36

GroupA18f 0 0 0 0 0 0 0 0 0 0 0 0 0 0 0 0 2 13 21 36

GroupA18g 0 0 0 0 0 0 0 0 0 0 0 0 0 0 0 0 1 12 23 36

GroupA18h 0 0 0 0 0 0 0 0 0 0 0 0 13 0 0 0 4 11 8 36

GroupA18i 0 0 0 0 0 0 0 0 0 0 0 0 0 12 0 0 0 6 0 18

GroupA19 48 8 7 5 0 10 3 3 4 6 2 5 0 0 0 1 2 22 0 126

Σ 897 310 117 137 0 454 236 159 167 342 24 258 38 132 0 31 186 723 379

# Total 4590

# c. Standardised real GPCR data

Group Phylogenetic event types (1-19 in order)

GroupA1 67 68 0 4 0 9 4 0 3 1 0 1 0 0 0 0 1 42 0

GroupA2a 48 36 5 5 0 9 7 0 8 5 2 9 8 8 2 2 2 45 0

GroupA3 63 14 4 1 0 6 4 0 1 3 1 1 7 11 7 0 3 61 13

GroupA4 70 40 3 1 1 10 4 3 2 3 1 3 2 7 3 0 6 35 5

GroupA5 39 21 1 4 1 9 6 3 1 3 0 4 4 8 16 1 5 66 7

GroupA6a 76 1 1 0 1 4 0 1 0 5 0 3 6 24 21 0 15 30 11

GroupA6b 33 25 0 0 0 0 0 0 0 0 0 0 8 25 3 0 10 75 23

GroupA7a 56 9 8 0 0 7 1 1 0 4 0 0 10 45 18 0 9 25 8

GroupA7b 46 14 2 3 0 6 1 2 1 7 5 4 18 15 17 1 9 48 1

GroupA8a 40 34 0 4 3 6 2 0 2 4 2 3 5 44 0 0 6 38 6

GroupA8b 62 75 0 5 5 5 3 1 8 0 1 1 0 22 0 0 0 12 0

GroupA9a 88 14 0 2 0 2 0 0 3 5 3 8 11 17 5 2 5 38 2

GroupA9b 36 10 2 0 1 7 1 1 0 9 0 3 12 63 11 1 9 31 1

GroupA10 74 16 4 2 0 3 0 0 0 3 0 1 17 11 15 0 10 22 21

GroupA11 57 21 3 6 0 15 12 1 6 12 0 4 5 5 7 1 6 33 7

GroupA12 44 47 2 3 0 5 5 2 2 5 2 2 0 8 8 0 0 64 2

GroupA13 57 38 2 6 0 19 13 4 4 8 1 13 3 10 13 0 0 11 0

GroupA14 38 26 1 2 0 12 1 0 2 0 0 1 6 24 28 0 11 37 11

GroupA15 35 18 2 7 0 7 17 3 9 7 2 11 2 9 19 0 5 47 1

GroupA16 92 6 2 4 0 8 6 0 4 4 4 0 4 0 6 2 2 42 13

GroupA17 47 15 3 4 4 8 6 3 4 3 3 11 7 13 43 0 6 18 3

GroupA18a 55 21 1 0 0 1 0 0 0 4 0 0 19 26 11 0 18 36 8

GroupA18b 63 11 0 0 0 0 0 0 0 0 0 0 8 3 11 0 5 79 21

GroupA19 55 23 1 2 1 2 1 0 0 0 1 1 11 26 10 0 1 51 11

Σ 1341 603 47 65 17 160 94 25 60 95 28 84 173 424 274 10 144 986 175

**d. Standardised simulated GPCR data**

Group Phylogenetic event types (1-19 in order)

GroupA1 75 22 6 4 0 22 11 0 4 8 0 6 0 3 0 3 1 26 8

GroupA2a 37 17 7 13 0 29 15 13 16 14 2 18 0 0 0 4 3 11 0

GroupA3 53 7 3 11 0 8 4 4 7 18 3 25 0 0 0 3 11 29 14

GroupA4 72 18 15 8 0 31 20 8 9 3 0 2 0 0 0 0 0 13 0

GroupA5 26 11 0 7 0 4 3 1 3 18 0 15 1 10 0 1 17 61 21

GroupA6a 16 3 13 3 0 8 2 0 2 17 0 3 2 6 0 0 8 48 70

GroupA6b 11 6 0 0 0 0 0 0 0 0 0 0 6 50 0 0 31 78 19

GroupA7a 24 5 5 2 0 3 0 0 2 19 0 5 2 10 0 0 11 62 52

GroupA7b 24 4 1 3 0 6 3 1 0 25 0 10 6 7 0 1 19 51 39

GroupA8a 30 21 10 4 0 37 20 10 13 10 1 4 0 0 0 0 6 33 0

GroupA8b 98 36 1 5 0 16 9 6 10 3 0 2 0 0 0 1 0 13 0

GroupA9a 72 13 0 7 0 0 0 0 2 15 6 13 0 2 0 17 6 41 7

GroupA9b 26 12 4 6 0 10 9 4 4 17 0 11 1 11 0 1 26 35 23

GroupA10 4 7 2 0 0 0 0 0 0 4 0 0 0 27 0 0 11 53 91

GroupA11 21 10 5 3 0 15 8 3 13 25 3 27 2 6 0 0 17 26 15

GroupA12 48 27 3 8 0 11 11 0 6 11 5 16 0 3 0 2 10 35 5

GroupA13 28 12 6 3 0 46 25 23 10 22 0 9 0 0 0 0 2 14 0

GroupA14 19 18 1 8 0 10 21 1 0 13 0 8 0 8 0 0 24 47 21

GroupA15 19 8 6 7 0 27 15 7 12 37 2 34 0 2 0 2 7 14 1

GroupA16 67 17 2 6 0 11 4 0 0 19 2 9 2 4 0 4 4 35 17

GroupA17 15 6 10 9 0 51 16 23 14 27 0 17 0 0 0 0 1 11 0

GroupA18a 35 13 4 19 0 9 4 0 0 4 4 9 2 15 0 2 20 35 26

GroupA18b 100 3 0 0 0 0 0 0 0 0 0 0 0 3 0 0 8 61 25

GroupA19 76 13 11 8 0 16 5 5 6 10 3 8 0 0 0 2 3 35 0

Σ 996 309 115 144 0 370 205 109 133 339 31 251 24 167 0 43 246 867 454

**Data 4. Supplementary Tables**event mean se  T crit sig

1 14.375 5.103 23 2.817 1.714 1

2 12.250 2.538 23 4.826 1.714 1

3 -2.833 0.904 23 -3.133 1.714 1

4 -3.292 0.994 23 -3.313 1.714 1

5 0.708 0.279 23 2.539 1.714 1

6 -8.750 2.562 23 -3.415 1.714 1

7 -4.625 1.368 23 -3.371 1.714 1

8 -3.500 1.254 23 -2.791 1.714 1

9 -3.042 0.795 23 -3.826 1.714 1

10 -10.167 1.577 23 -6.446 1.714 1

11 -0.125 0.401 23 -0.305 1.714 0

12 -6.958 1.570 23 -4.432 1.714 1

13 6.208 1.016 23 6.101 1.714 1

14 10.708 3.497 23 3.062 1.714 1

15 11.417 2.031 23 5.620 1.714 1

16 -1.375 0.636 23 -2.160 1.714 1

17 -4.250 1.437 23 -2.957 1.714 1

18 4.958 3.761 23 1.315 1.714 0

19 -11.625 4.261 23 -2.728 1.714 1

**Supplementary Table 1**.

A 2 pair T- test was carried out for each of the nineteen phylogenetic outcomes comparing standardised real and simulated data. Critical values of T are at the 5% level, significant differences are given as 1 in the right hand column, or zero if there is no significant difference.

jumps/window null fragments/window

real simulated real simulated

groupa1 0.2407 0.1111 0 0.0117

groupa2a 0.2453 0.0451 0.0491 0.0263

groupa3 0.2993 0.1382 0.0408 0.0263

groupa4 0.1794 0.0622 0.009 0

groupa5 0.3462 0.2698 0.0165 0.0132

groupa6a 0.1714 0.2256 0.0343 0.0075

groupa6b 0.25 0.3421 0.0682 0.0263

groupa7a 0.1595 0.2932 0.0982 0

groupa7b 0.2552 0.2434 0.0781 0.026

groupa8a 0.2328 0.1579 0.0212 0.0053

groupa8b 0.0652 0.0611 0.0073 0.0042

groupa9a 0.2031 0.1929 0.0703 0.1053

groupa9b 0.2367 0.1579 0.0966 0.0117

groupa10 0.1353 0.2526 0.0821 0

groupa11 0.1774 0.1538 0.0264 0.0526

groupa12 0.2975 0.1654 0.0083 0.0301

groupa13 0.0627 0.0673 0.035 0

groupa14 0.2242 0.2237 0.0242 0

groupa15 0.2537 0.0681 0.0177 0.0186

groupa16 0.1959 0.1667 0.0515 0.0351

groupa17 0.1223 0.0503 0.055 0

groupa18a 0.1895 0.1667 0.0784 0.0351

groupa18b 0.4202 0.2763 0.029 0

groupa19 0.3061 0.1654 0.068 0.0226

**Supplementary Table 2.**

Average number of events per window for each GPCR group, real and simulated. Columns two and three represent jump events and columns four and five represent null fragments. The null fragments in this case are due only to high distances and not missing data to gaps in alignments.

frequency rank frequency rank

groupA1 0.83 4 0 1.5

groupA2a 10.76 38.5 1.09 7.5

groupA3 14.9 42 6.54 30

groupA4 6.62 32.5 3.27 16

groupA5 15.73 43 6.54 30

groupA6a 11.59 40.5 5.45 24.5

groupA6b 5.79 27.5 2.18 12

groupA7a 0.83 4 6.54 30

groupA7b 3.31 18.5 2.18 12

groupA8a 2.48 14 5.45 24.5

groupA8b 4.14 20 3.27 16

groupA9a 6.62 32.5 2.18 12

groupA9b 10.76 38.5 1.09 7.5

groupA10 1.66 10 5.45 24.5

groupA11 25.66 47 7.63 34

groupA12 18.21 44 5.45 24.5

groupA13 5.79 27.5 8.71 36.5

groupA14 3.31 18.5 1.09 7.5

groupA15 43.04 48 4.36 21.5

groupA16 0.83 4 0 1.5

groupA17 24.83 46 8.71 36.5

groupA18a 8.28 35 1.09 7.5

groupA18b 11.59 40.5 4.36 21.5

groupA19 23.17 45 3.27 16

Σ 721 455

U 131 397

**Supplementary Table 3**

Mann Whitney Test for standardised frequencies of jump events that cross 1-4 nodes in the GPCR tree (figure 1). The value for U_crit_ at the 0.01 level is 175. The value U_1_ is 131, which is lower than U_crit_ so the null hypothesis is rejected, the real data has a significantly higher frequency of jump events across 1-4 nodes than the null data.

frequency rank frequency rank

groupA1 0 4.5 1.17 15.5

groupA2a 4.91 36 2.63 26

groupA3 4.08 35 2.63 26

groupA4 0.9 14 0 4.5

groupA5 1.65 18 1.32 17

groupA6a 3.43 31 0.75 12

groupA6b 6.82 41 2.63 26

groupA7a 9.82 47 0 4.5

groupA7b 7.81 43 2.6 24

groupA8a 2.12 21 0.53 10

groupA8b 0.73 11 0.42 9

groupA9a 7.03 42 10.53 48

groupA9b 9.66 46 1.17 15.5

groupA10 8.21 45 0 4.5

groupA11 2.64 28 5.26 38

groupA12 0.83 13 3.01 30

groupA13 3.5 32 0 4.5

groupA14 2.42 23 0 4.5

groupA15 1.77 19 1.86 20

groupA16 5.15 37 3.51 33.5

groupA17 5.5 39 0 4.5

groupA18a 7.84 44 3.51 33.5

groupA18b 2.9 29 0 4.5

groupA19 6.8 40 2.26 22

Σ 738.5 437.5

U 113.5 414.5

**Supplementary Table 4**

Mann Whitney Test for standardised frequencies of null events. The value for U_crit_ at the 0.01 level is 175. The value U_1_ is 113.5, which is lower than U_crit_ so the null hypothesis is rejected, the real data has a significantly higher frequency of null events than the simulated data.
