# Supplementary material for: Phylogenomic Analysis Reveals Extensive Phylogenetic Mosaicism in the Human GPCR Superfamily
Source: Evol Bioinform Online. 2007 Sep 26;3:357–70. (PMC2684142)
Supplement: Supplementary Tables [file ebo-03-357-s5.doc]

**Data 4. Supplementary Tables**event mean se  T crit sig

1 14.375 5.103 23 2.817 1.714 1

2 12.250 2.538 23 4.826 1.714 1

3 -2.833 0.904 23 -3.133 1.714 1

4 -3.292 0.994 23 -3.313 1.714 1

5 0.708 0.279 23 2.539 1.714 1

6 -8.750 2.562 23 -3.415 1.714 1

7 -4.625 1.368 23 -3.371 1.714 1

8 -3.500 1.254 23 -2.791 1.714 1

9 -3.042 0.795 23 -3.826 1.714 1

10 -10.167 1.577 23 -6.446 1.714 1

11 -0.125 0.401 23 -0.305 1.714 0

12 -6.958 1.570 23 -4.432 1.714 1

13 6.208 1.016 23 6.101 1.714 1

14 10.708 3.497 23 3.062 1.714 1

15 11.417 2.031 23 5.620 1.714 1

16 -1.375 0.636 23 -2.160 1.714 1

17 -4.250 1.437 23 -2.957 1.714 1

18 4.958 3.761 23 1.315 1.714 0

19 -11.625 4.261 23 -2.728 1.714 1

**Supplementary Table 1**.

A 2 pair T- test was carried out for each of the nineteen phylogenetic outcomes comparing standardised real and simulated data. Critical values of T are at the 5% level, significant differences are given as 1 in the right hand column, or zero if there is no significant difference.

jumps/window null fragments/window

real simulated real simulated

groupa1 0.2407 0.1111 0 0.0117

groupa2a 0.2453 0.0451 0.0491 0.0263

groupa3 0.2993 0.1382 0.0408 0.0263

groupa4 0.1794 0.0622 0.009 0

groupa5 0.3462 0.2698 0.0165 0.0132

groupa6a 0.1714 0.2256 0.0343 0.0075

groupa6b 0.25 0.3421 0.0682 0.0263

groupa7a 0.1595 0.2932 0.0982 0

groupa7b 0.2552 0.2434 0.0781 0.026

groupa8a 0.2328 0.1579 0.0212 0.0053

groupa8b 0.0652 0.0611 0.0073 0.0042

groupa9a 0.2031 0.1929 0.0703 0.1053

groupa9b 0.2367 0.1579 0.0966 0.0117

groupa10 0.1353 0.2526 0.0821 0

groupa11 0.1774 0.1538 0.0264 0.0526

groupa12 0.2975 0.1654 0.0083 0.0301

groupa13 0.0627 0.0673 0.035 0

groupa14 0.2242 0.2237 0.0242 0

groupa15 0.2537 0.0681 0.0177 0.0186

groupa16 0.1959 0.1667 0.0515 0.0351

groupa17 0.1223 0.0503 0.055 0

groupa18a 0.1895 0.1667 0.0784 0.0351

groupa18b 0.4202 0.2763 0.029 0

groupa19 0.3061 0.1654 0.068 0.0226

**Supplementary Table 2.**

Average number of events per window for each GPCR group, real and simulated. Columns two and three represent jump events and columns four and five represent null fragments. The null fragments in this case are due only to high distances and not missing data to gaps in alignments.

frequency rank frequency rank

groupA1 0.83 4 0 1.5

groupA2a 10.76 38.5 1.09 7.5

groupA3 14.9 42 6.54 30

groupA4 6.62 32.5 3.27 16

groupA5 15.73 43 6.54 30

groupA6a 11.59 40.5 5.45 24.5

groupA6b 5.79 27.5 2.18 12

groupA7a 0.83 4 6.54 30

groupA7b 3.31 18.5 2.18 12

groupA8a 2.48 14 5.45 24.5

groupA8b 4.14 20 3.27 16

groupA9a 6.62 32.5 2.18 12

groupA9b 10.76 38.5 1.09 7.5

groupA10 1.66 10 5.45 24.5

groupA11 25.66 47 7.63 34

groupA12 18.21 44 5.45 24.5

groupA13 5.79 27.5 8.71 36.5

groupA14 3.31 18.5 1.09 7.5

groupA15 43.04 48 4.36 21.5

groupA16 0.83 4 0 1.5

groupA17 24.83 46 8.71 36.5

groupA18a 8.28 35 1.09 7.5

groupA18b 11.59 40.5 4.36 21.5

groupA19 23.17 45 3.27 16

Σ 721 455

U 131 397

**Supplementary Table 3**

Mann Whitney Test for standardised frequencies of jump events that cross 1-4 nodes in the GPCR tree (figure 1). The value for U_crit_ at the 0.01 level is 175. The value U_1_ is 131, which is lower than U_crit_ so the null hypothesis is rejected, the real data has a significantly higher frequency of jump events across 1-4 nodes than the null data.

frequency rank frequency rank

groupA1 0 4.5 1.17 15.5

groupA2a 4.91 36 2.63 26

groupA3 4.08 35 2.63 26

groupA4 0.9 14 0 4.5

groupA5 1.65 18 1.32 17

groupA6a 3.43 31 0.75 12

groupA6b 6.82 41 2.63 26

groupA7a 9.82 47 0 4.5

groupA7b 7.81 43 2.6 24

groupA8a 2.12 21 0.53 10

groupA8b 0.73 11 0.42 9

groupA9a 7.03 42 10.53 48

groupA9b 9.66 46 1.17 15.5

groupA10 8.21 45 0 4.5

groupA11 2.64 28 5.26 38

groupA12 0.83 13 3.01 30

groupA13 3.5 32 0 4.5

groupA14 2.42 23 0 4.5

groupA15 1.77 19 1.86 20

groupA16 5.15 37 3.51 33.5

groupA17 5.5 39 0 4.5

groupA18a 7.84 44 3.51 33.5

groupA18b 2.9 29 0 4.5

groupA19 6.8 40 2.26 22

Σ 738.5 437.5

U 113.5 414.5

**Supplementary Table 4**

Mann Whitney Test for standardised frequencies of null events. The value for U_crit_ at the 0.01 level is 175. The value U_1_ is 113.5, which is lower than U_crit_ so the null hypothesis is rejected, the real data has a significantly higher frequency of null events than the simulated data.
